# Adamantane-based inhibitors of the influenza A M2 proton channel: structure-based design, biological evaluation, and synthetic approaches

**DOI:** 10.1039/d5md01027f

**Published:** 2026-02-23

**Authors:** Marianna Stampolaki, Maria-Eleni Kouridaki, Kyriakos Georgiou, Antonios Kolocouris

**Affiliations:** a Laboratory of Medicinal Chemistry, Section of Pharmaceutical Chemistry, Department of Pharmacy, National and Kapodistrian University of Athens Panepistimiopolis-Zografou Athens 15771 Greece ankol@pharm.uoa.gr; b Department of NMR Based Structural Biology, Max Planck Institute for Multidisciplinary Sciences Am Fassberg 11 Göttingen Germany

## Abstract

The influenza A matrix 2 (AM2) protein, a prototype of viroporins, conducts protons along a chain of water molecules and ionizable side chains, including histidine-37. Solid-state NMR (ssNMR) and high-resolution X-ray crystal structures have been obtained for AM2 wild-type (WT) constructs in complex with adamantanamines, as well as for mutant AM2 channels that confer resistance to amantadine and adamantanamines across different pH levels. For the structure of AM2 S31N channels in complex with second-generation adamantane derivatives, consisting of an amantadine analog linked to an aryl group *via* a methylene bridge, X-ray crystal structures are still unavailable. These complexes have been studied in some detail to date, primarily using solution NMR spectroscopy in micelles or ssNMR in lipid bilayers, providing insights into the inhibition mechanisms of these drugs. These findings, when combined with advances in computational methods, can inform the design and synthesis of adamantane-based blockers targeting WT and mutant AM2 channels. The most popular testing assays were presented. Selected synthetic chemistry routes leading to complex adamantanamines, other saturated polycyclic amines, and second-generation adamantane-based inhibitors were provided. Extensive and long-term research on the druggable M2 channel has provided the scientific society with fundamental tools of structure-based drug design, a synthetic chemistry toolbox, and a library of adamantane-based compounds that can be useful antivirals due to the frequent viral AM2 mutations.

## Abstract 2

Experimental structures and accurate simulations can guide the design and synthesis of adamantanamines that can block AM2 wild-type and mutant AM2-mediated proton conductance.

## Graphical abstract

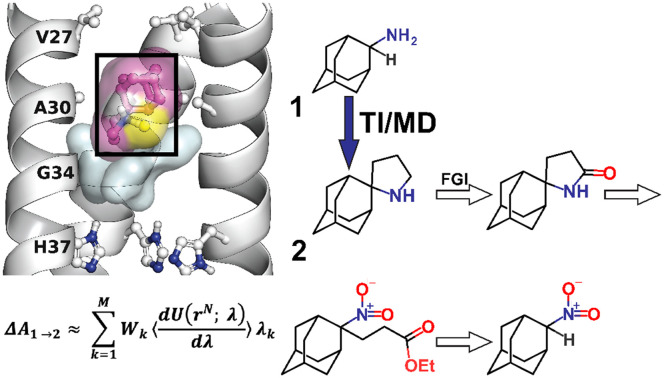

## Introduction

### Purpose of the review article

Previous review articles have been published, including: (a) The structure and function of the influenza A M2 (AM2) proton channel^1–7^ and its role in virus assembly and budding.^8^ (b) The molecular basis of inhibition of AM2 viroporin function by adamantanamines.^4,6,7,9,10^ (c) The structure of adamantanamines and other saturated polycyclic amines that block AM2 wild-type (WT) (with serine at position 31), as well as adamantane-based drugs, that block mutant AM2-mediated proton currents studied through electrophysiology (EP).^11–17^ (d) The synthetic routes for selected adamantanamines.^18,19^ (e) The structures of other compounds that inhibit influenza A virus replication.^20–22^ We reviewed the development of adamantane- and other polycyclic amine-based blockers targeting either AM2 WT or mutant AM2 proteins and their associated viruses based on key experimental structures of AM2 constructs. Our discussion included the assays used to measure potency and the structure-based drug design methods. Finally, we reported selected synthetic routes for adamantanamines, saturated polycyclic amines, and second-generation adamantane-based derivatives, that consist of an amantadine analog linked to an aryl group *via* a methylene bridge.

### Functional role of influenza AM2 protein

**Scheme 1:**
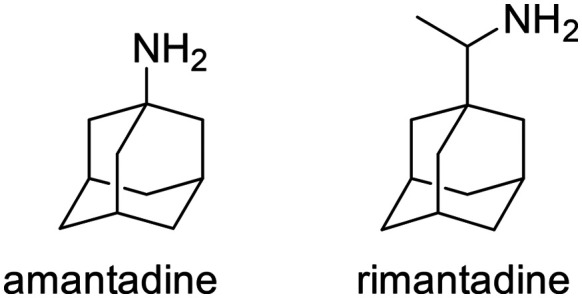
Chemical structures of amantadine and racemic rimantadine.

The AM2 proton channel plays a crucial role in the life cycle of the influenza A virus. During the early phase of infection, the virus enters the host cell through endocytosis. The AM2 channel is activated in response to the lowered pH within the endosome, which leads to the transport of protons into the viral interior. This acidification of the interior of the virus triggers the dissociation of the viral RNA from the M1 protein,^23,24^ resulting in virus uncoating. This processs, faciltated by a conformational change in hemagglutinin, leads to the fusion of the membranes of the virus and the host cell endosome.^25,26^ During the later stages of virus replication, during virus cellular exit, the AM2 protein maintains the integrity of the hemagglutinin during virus assembly in the *trans*-Golgi network. This function is inhibited by amantadine or rimantadine (see Scheme 1).^25^ The AM2 protein helps to maintain the high pH of the *trans*-Golgi network, thereby preventing the premature conformational change of hemagglutinin that occurs at low pH levels and triggers hemagglutinin-induced fusion.^27^ The AM2 facilitates viral exit by curving the host cell membrane, enabling membrane scission and virus budding.^28,29^

The AM2 is a 97-residue membrane protein that assembles and functions as a homo-tetramer, allowing it to conduct protons when activated by low pH, a process essential for the viral life cycle.^30–35^ It features a short unstructured N-terminal extracellular ectodomain, made up of residues 1–21,^36,37^ followed by a single transmembrane (TM) domain, made up of residues residues 22–46, known as AM2TM. Finally, it contains a long C-terminal intracellular tail, made us of residues 47–97, which is in contact with the M1 protein.^38^

The proton-selective ion channel of AM2 is located within the AM2TM domain.^39^ This domain is the minimal structure required for tetramerization, selective proton transport,^30,32,33^ and amantadine binding.^40–44^ The conductance rate of the AM2TM domain and the blocking of the proton current by amantadine, as measured with EP,^45,46^ correspond to those of the full-length AM2 protein (AM2FL).^39,47^ Near the C-terminal of the TM bundle, there is a region that features an interruption by the H37xxxW41 sequence in the C-terminal half of the TM helix. In this region, polar and single charged residues face the channel, while and hydrophobic residues are oriented towards the lipid bilayer.^48^ In the AM2TM domain, residue H37 plays a key role in activation as the proton-conducting residue of the channel.^49,50^ At low pH in the viral endosome, the imidazole rings in the four H37 residues causes the channel to open due to electrostatic repulsion and destabilization of helix–helix packing. W41 is the residues that controls, the gate blocking, and proton flow when the channel is in the alkaline pH state.^51,52^ Besides the well known gating role of W41, V27 has been suggested to create a secondary gate.^51,52^ The cavity between V27 and H37 contains a binding site for amantadine.^53^ Water molecules within the channel pore create a hydrogen-bonded water network along the 17 Å stretch between the V27 valve and H37 tetrad.^54,55^ Functional^56^ and structural studies^54,55^ have demonstrated that the pore is formed by the TM-helix bundle and is lined by V27, S31, G34, H37, W41, D44, and R45, *i.e.*, includes all the polar residues of the TM sequence.

The amphipathic helix (AH), consisting of residues 47–62, is positioned between the TM helix and the M1-binding segment of AM2. The AHs are located at the inner membrane interface and are essential for viral budding.^8^ It is believed that AHs create the membrane curvature necessary for viral budding.^57,58^ The TM and AH bundle comprises the AM2TM + AH, collectively known as the conductance domain (CD) AM2CD. In synthetic lipid bilayers, AM2CD appears to contain all the necessary residues and environmental interactions to achieve the detailed conductance properties of the native protein found in oocytes.^39^

## Experimental structures of AM2 bound with adamantane-based derivatives

### Wild-type AM2 protein

The structure of the apo-AM2TM^48^ or apo-AM2CD^59^ was studied using oriented sample (OS) ssNMR in phospholipid bilayers at pH 7, showing a fourfold symmetric homotetramer; the structure with PDB ID 2L0J^59^ was deposited for AM2CD. The charge of H37 tetrad was +2 charged between pH 6–7.5, and the charges may be extensively delocalized over a dimer-of-dimers structure. The kink in the TM helix occurs around G34, like the kinked TM-domain structure in the AM2TM protein, with the presence of amantadine.^60^ At pH 8, the imidazole rings of the four H37 residues are unprotonated. The first protonation of H37 forms an imidazole–imidazolium dimer with a strong hydrogen bond between bridged imidazole rings. The second protonation forms two imidazole–imidazolium dimers.^48,59^ At pH 7.5, most of the H37 side chains existed as imidazole–imidazolium dimer. At pH 5.0, all the H37 are protonated.

The first X-ray crystal structures of samples of apo-AM2TM WT or AMTM WT with amantadine,^61^ showed a tetrameric left-handed bundle, which was consistent with a previous solid-state NMR (ssNMR) structure of the apo-AM2TM (PDB ID 1NYJ^62^) in phospholipid bilayers. While the apo-AM2TM WT crystal structure had a 2.05 Å resolution and an I33M mutation (PDB ID 3BKD^61^), the latter crystal structure (PDB ID 3C9J^61^), which was suggested to correspond to a complex with amantadine inside the TM pore, had a 3.5 Å resolution and the stabilizing mutation G34A.^61,63^ However, the 3.5 Å resolution of this structure did not ensure the presence of amantadine (having a diameter of ∼3.4 Å). Additionally, the 3.5 Å resolution of PDB ID 3C9J^61^ did not allow for examining the critical role of water in drug binding. One of the proposed proton conduction mechanisms is mediated by ordered water molecules arranged in wires inside the channel, which play a role in the conduction of protons through the AM2 pore.^64,65^ In another work, a potential allosteric binding outside the pore was suggested in the complex of rimantadine with AM2CD in micelles (PDB ID 2RLF^66^) using solution NMR in micelles. In this site, the ammonium group of rimantadine in the secondary binding site is in contact with the polar sidechain of D44, whereas the adamantyl group of the drug forms hydrophobic interactions with I42 from one of the TM helices and with L40 and L43 from the adjacent helix. However, this structure was solved with a 200-fold excess of rimantadine, while the micelles did not allow proper folding, narrowing the tetrameric bundle in the N-end and disturbing the rimantadine entrance to the channel. Nevertheless, in the later ssNMR structure of a sample of AM2TM with a perdeuterated amantadine in phospholipid bilayers (PDB ID 2KQT^67^) obtained using experimental restraints from a previous structure (PDB ID 2H95^60^), amantadine was found inside the pore between V27 and H37, as previously suggested.^42,52,60^ Perdeuterated amantadine^68^ was used in complex with AM2AH in dimyristoyl-*sn-glycero*-3-phosphocholine (DMPC) bilayers, and it was shown with OS ssNMR that amantadine binds close to S31 inside the pore, as was shown in the PDB ID 2KQT structure.^67^ Additionally, it was also shown using ssNMR and CD_3_-labeled rimantadine that rimantadine has its amino group towards the C-end.^69^ Thus, the pharmacologically relevant drug-binding site is in the TM pore of the fully functional AM2CD. Other studies with AM2CD and rimantadine using ssNMR in phospholipid bilayers^70^ or solution NMR in micelles (PDB ID 2LJC^71^) also showed the presence of rimantadine inside the AM2 pore.

**Fig. 1:**
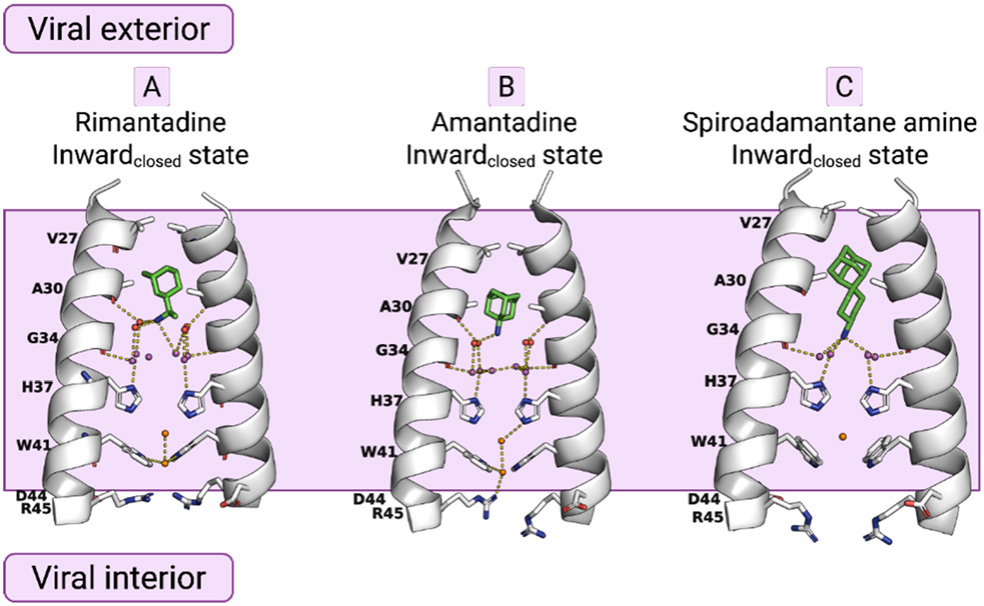
X-ray crystal structures of the AM2TM proton channel bound to drugs and inhibitors.^72^ Top, left to right: (A) AM2TM bound to rimantadine (PDB ID 6BKL^72^). (B) AM2TM bound to amantadine (PDB ID 6BKK^72^). (C) AM2TM bound to a spiramic adamantanamine (PDB ID 6BMZ^72^). This figure has been adapted from ref. 6 with permission from ELSEVIER, copyright 2025.

Eventually, the most direct evidence of the pore binding site and water wire role for the stabilization of channel blockers by EP inside the pore came from higher resolution crystallographic studies of the AM2TM in the presence of adamantanamine. Indeed, water-mediated interactions in receptor binding sites can play key roles in drug binding and inhibitor design. Experimental structures with high resolution made the detection of crystallographically important waters possible. The detection of crystallographically important waters demonstrated the channel inhibition by inhibitors targeting these key waters. This was shown in structures of AM2TM WT bound to amantadine (PDB ID 6BKK;^72^ 2.00 Å), rimantadine (PDB ID 6BKL;^72^ 2.00 Å), or a spiro-adamantanamine inhibitor (PDB ID 6BMZ;^72^ 2.63 Å), see Fig. 1. These X-ray structures of AM2TM ion channel with bound inhibitors reveal that the ammonium group of the drugs is directed toward H37 and binds to water-lined sites that are hypothesized to stabilize transient hydronium ions formed in the proton-conduction mechanism. As supported by molecular dynamics (MD) simulations, the ammonium group is stabilized through three hydrogen bonds with proximal waters, creating a layer at the level of the A30 layer, followed by the G34-water layer and the H37 tetrad. The placement of the adamantanamine molecules within the pore requires a displacement of waters near V27 by the hydrophobic adamantane ring of the above adamantanamine molecules, thus disturbing the AM2 water network.^72^ The MD simulations^72^ of the AM2TM WT, with the three adamantanamine drugs predicted with accuracy the position of the ligands and waters inside the pore in the X-ray crystal structure of the AM2TM WT complexes. In another work, the X-ray structures of AM2TM WT pore bound to (*R*)-rimantadine (PDB ID 6US9^73^) and (*S*)-rimantadine (PDB ID 6US8^73^) were solved with a 2.00 Å resolution, showing only slight differences in the hydration of each enantiomer.

### Mutant AM2 proteins

Amantadine resistance mutations are found close to the drug-binding site located at the pore-facing positions (V27A, A30T, S31N, and G34E), at the interhelical interfaces at the N-terminal half of the channel (L26F), and mutations outside the drug-binding site lying at the interhelical interfaces (L38F, D44). It was suggested^74^ that a mutated, drug-resistant protein has a larger binding pocket for the drug, as shown with MD simulations.^75^ Hence, despite binding to the channel, the drug remains sufficiently mobile so as not to exert a proton-blocking positive electrostatic hindrance as a simple mechanism for proton flow blocking in AM2 WT by amantadine.^75^

**Fig. 2:**
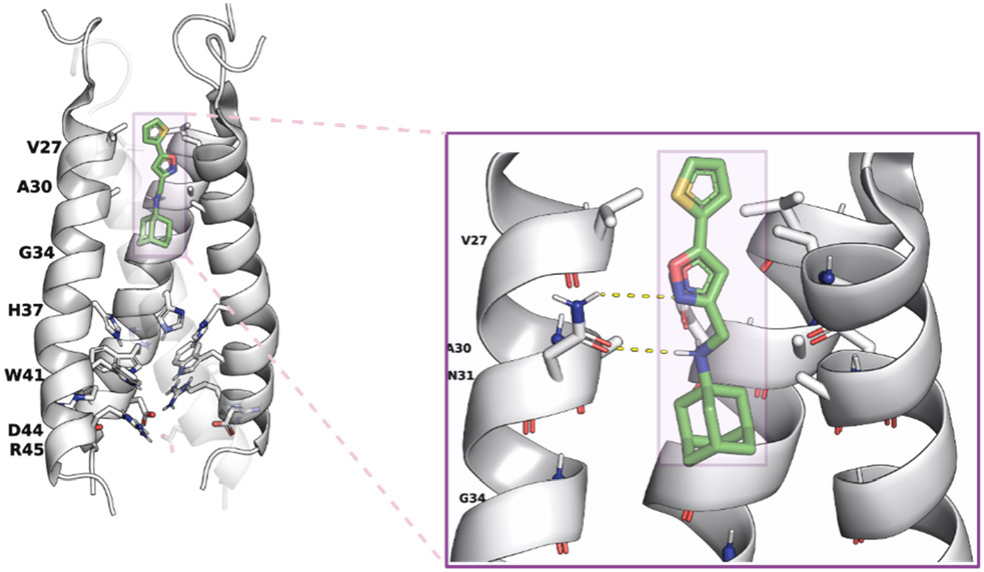
AM2 (19–49) S31N drug inhibition mechanism.^76^ Left part: binding site of M2WJ332 from the solution NMR structure (PDB ID 2LY0^76^): the side chains V27, A30, H37, W41, and the molecule are shown as colored sticks; other side chains are shown as transparent space filling; the backbone of the protein (one monomer was omitted for clarity) is shown as opaque ribbons. Right part: zoom in showing the drug–protein interactions: one of the N31 side chains forms bidentate interactions with the drug (the carbonyls from another two N31 residues can form a water-mediated hydrogen bond with the ammonium from the drug, which is not shown). This figure has been adapted from ref. 6 with permission from ELSEVIER, copyright 2025.

A solution NMR structure of AM2 (19–49) S31N in complex with M2J332^76^ in micelles (PDB ID 2LY0^76^ was reported. M2J332^76^ is a representative second-generation adamantane-based inhibitor that blocks M2 S31N-mediated current and replication of M2 S31N virus (Fig. 2); it can be considered a second-generation adamantane-based compound. Such compounds have been developed to inhibit influenza A M2 S31N which became the major epidemic virus after 2008. Indeed, in 2009, the emergence of the novel pandemic H1N1 influenza virus (A/H1N1/pdm09) led to a dramatic spike in hospitalizations and deaths in the United States, particularly among young adults.^77^ These drugs consist of amantadine connected through a methylene linker with aryl groups, *e.g.*, the amantadine-5-(thiophenyl)oxazolyl (M2J332^76^), amantadine-5-phenyloxazolyl (M2J352^76^), or amantadine-5-cyclopropyloxazolyl^78^ conjugates. With the structure PDB ID 2LY0,^76^ it was shown that these amantadine–aryl conjugates blocked AM2AH S31N with an orientation of the amantadine amino and aryl groups towards the N-end, as also demonstrated in the ssNMR study of M2J352,^79^ compared to adamantanamines, which point their amino groups towards the C-end of the AM2 WT channel according to the observations from ssNMR^80^ and X-ray structures.^72,73^ M2J332,^76^ and M2J352^76^ were able to block AM2 S31N by EP, and virus replication but did not block AM2 WT by EP and virus replication. Interestingly, the amantadine–3-bromo-thiophenyl conjugate (cmp11 in ref. 81) was a double AM2 S31N and WT blocker by EP. The solution NMR structure of the amantadine–3-bromo-thiophenyl conjugate with AM2 (19–49) WT (PDB ID MUW^81^) or AM2 (19–49) S31N (PDB ID 2MUV^81^) in micelles showed an orientation of the amantadine amino and aryl groups towards the N-end in AM2 (19–49) S31N and towards the C-end in AM2 WT.^81^

**Fig. 3:**
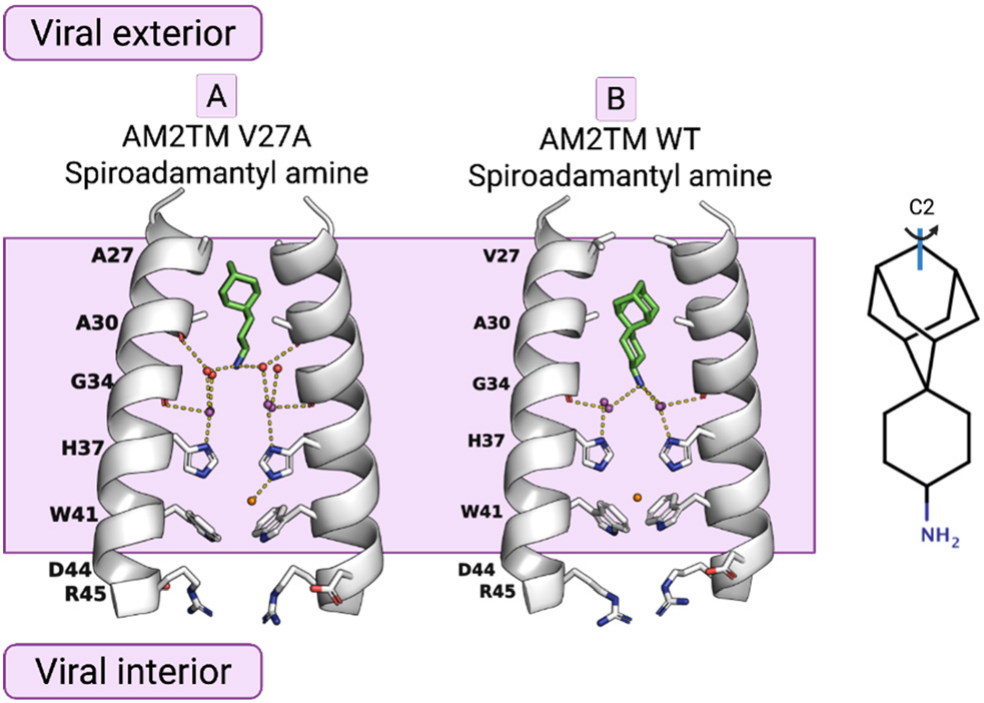
The mechanism of amantadine drug resistance in the AM2 V27A mutant channel and the mechanism of dual inhibition by the shown spiranic adamantanamine can be rationalized through the crystal structures of this adamantanamine with (A) AM2TM WT (PDB ID 6BMZ^72^) and (B) AM2TM V27A (PDB ID 6NV1^82^). This figure has been adapted from ref. 6 with permission from ELSEVIER, copyright 2025.

X-ray crystallography was used to solve the structures of a spiranic adamantanamine inhibitor bound to AM2TM V27A (PDB ID 6NV1^82^), shown in Fig. 3. This spiranic adamantanamine inhibitor was a triple blocker by EP of AM2 WT, V27A, and L26F proteins.^83^ The X-ray structure of the spiranic adamantanamine with AM2TM WT (PDB ID 6BMZ^72^) is shown in Fig. 1. The X-ray structures with PDB IDs 6BMZ^72^ and 6NV1^82^ revealed the mechanism of amantadine resistance. As shown in the crystal structure, compared to the AM2TM WT (Fig. 3B), the AM2TM V27A channel pore was wider at its N-terminus because of the V27A mutation, which removed V27 side chain hydrophobic interactions that are important for binding of amantadine and rimantadine (Fig. 3A). The spiranic adamantanamine inhibitor, which is longer than amantadine, shifted its binding site in the pore depending on which residue is present at position 27, thus blocking effective proton conductance in the AM2 WT and V27A mutant channels. The MD simulations of the AM2TM V27A–spiranic adamantanamine complex agreed with the experimental structures.^82^ The same argument can be applied to explain the EP-based blocking of the wide-diameter AM2TM L26F mutant by this spiranic adamantanamine.

## Resistance of influenza A virus to adamantane-based drugs

The most common AM2 inhibitors are amantadine derivatives. In 1966, amantadine was first approved as a prophylactic drug against Asian influenza.^84,85^ After a few years, it was announced^86^ that dyskinesia symptoms of a Parkinson's disease patient were remarkably improved while amantadine was administered for the prevention of flu. The mechanism of action of amantadine against these targets results from the blockage of ion channels either by direct interaction with the channel's pore (influenza AM2) or by stabilization of closed states of the channel (NMDA receptor).^87^ Since 1985, AM2 has been identified as the primary target of amantadine.^40^ Resistance to AM2 WT proton channel drugs, amantadine and rimantadine, is associated with mutations in the TM domain of the AM2 WT protein.^103^ The homo-tetrameric structure of the AM2 WT channel places constraints on the types of drug-resistant mutations that can be accommodated.^74,88^ A few amantadine- or rimantadine-resistant mutations have been observed in transmissible viruses,^89–91^ which are L26F, V27A, A30T, G34E, and S31N, located in the TM pore of AM2 WT,^89–94^ although other mutations can easily be observed *in vitro*. The mutations that cause the greatest decrease in inhibition are S31N and V27A, which increase the polarity of the pore. The vast majority, 95% of resistant viruses bear the S31N substitution in AM2, 1% have V27A, and L26F, A30T, and G34E are rare.^95,96^ The AM2 S31N mutant is one of the most conserved in viral proteins among currently circulating influenza A viruses, which happens to maintain nearly identical channel function as the AM2 WT but is resistant to amantadine, rimantadine, and adamantanamines. The presence of L26F, V27A, and particularly S31N in influenza A viruses circulating worldwide pushes the search for novel ion channel blockers with stronger, preferably resistance-overcoming activity.

Recently, the World Health Organization reported that influenza activity has globally increased since October 2025, with influenza A virus being predominant among the viruses (WHO, 10 December 2025, Disease Outbreak News; Seasonal influenza-Global situation). Currently circulating in humans are subtype A(H1N1) and A(H3N2) influenza viruses. Both subtypes include the S31N mutation in the M2 sequence. A recent work,^97^ showed that prolonged treatment with the neuraminidase (NA) inhibitors like the commonly used for influenza treatment, oseltamivir, can cause resistant mutations at hemagglutinin (HA) alone or in combination with resistant mutants at NA as a compensatory mechanism. In that way, the virus reduces receptor binding and restores functional balance between HA and NA. It was shown^97^ that these mutations can also confer cross-resistance to other NA inhibitors, including zanamivir and peramivir, in the A(H1N1)pdm09 virus. Considering (a) the prevalence of M2 S31N strains and (b) the ability of the virus to develop mutations in both HA and NA and become resistant to the currently used NA inhibitors, the design of new second-generation M2 inhibitors is becoming increasingly relevant. In another work,^98^ the second-generation drug resistance mechanism of the virus towards second-generation M2 channel blockers was profiled *in vitro*. Serial viral passage experiments were performed^98^ in the A/California/07/2009 (H1N1) viral strain under persistent drug selection pressure to identify resistant mutants against such blockers. The results suggested^98^ that these S31N inhibitors have a higher genetic barrier to drug resistance than amantadine in cell culture. It was shown that the evolved mutations are all located at the N-terminal drug binding site of AM2 S31N, and they have either a direct or indirect effect on the channel pore, which leads to either reduced or complete loss of drug sensitivity. More specifically, those mutations introduced either a structural hindrance for the new agents as their aryl groups lie in this region or change the polarity of that site of the tetramer, affecting their entrance to the pore.^99,100^ This information is quite important for optimizing the structure of new selective S31N inhibitors.

## Methods to detect inhibition of wild-type and mutant AM2 viruses by adamantane-based drugs

### Cell antiviral assays

**Fig. 4:**
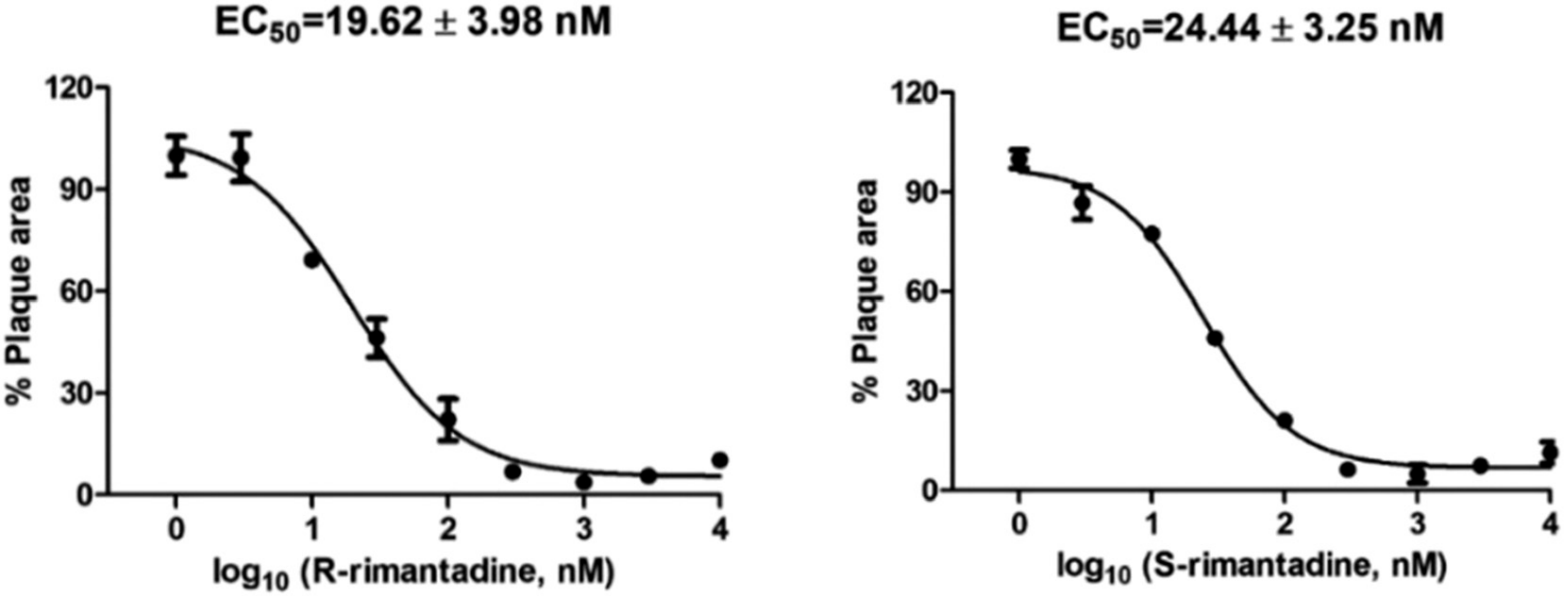
Cellular antiviral assay results of (*R*)- and (*S*)-rimantadine against the amantadine-sensitive A/Solomon Island/3/2006 (H1N1) strain. The antiviral potency was determined in a plaque assay. The EC_50_ values are the mean ± standard deviation of two independent repeats. This figure has been reproduced from ref. 73 with permission from the American Chemical Society, copyright 2021.

Compounds have been tested as inhibitors of viral replication using cell assays. These are most often the miniplaque assay,^101–104^ and the cytopathic effect (CPE) inhibition assay,^105^ performed in Madin-Darby canine kidney (MDCK) cells against influenza virus strains. Mutant AM2 viruses can be produced using reverse genetics.^106^ For example, using a miniplaque assay, the (*R*)- and (*S*)-enantiomers of rimantadine showed indistinguishable potency against the amantadine-sensitive A/Solomon Island/3/2006 (H1N1) strain. Both (*R*)- and (*S*)-rimantadine inhibit viral replication with similar EC_50_ values of 19.62 and 24.44 nM, respectively (Fig. 4).^73^

An *Escherichia coli*-based assay was used to measure the H^+^ conductivity of AM2 WT^107^ or mutants M2^108,109^ in the presence of a potential inhibitor, since bacteria that constitutively express a pH-sensitive green fluorescent protein—pHluorin, can be used to analyze the membrane permeation to H^+^. Specifically, the emission at 520 nm of pHluorin has two excitation maxima: 390 nm and 466 nm, whose ratio changes as a function of pH. The fluorescence obtained when exciting at 390 nm and 466 nm upon injection of a concentrated acid solution into the buffer was monitored. Any bacteria that express an H^+^-conducting channel will exhibit a dramatic change in the fluorescence ratio, in contrast to control bacteria that contain an empty vector or when an inhibitor is present.

### Assays that measure direct binding

#### Experiments that measure the thermodynamics of binding and proton flux blocking experiments

**Fig. 5:**
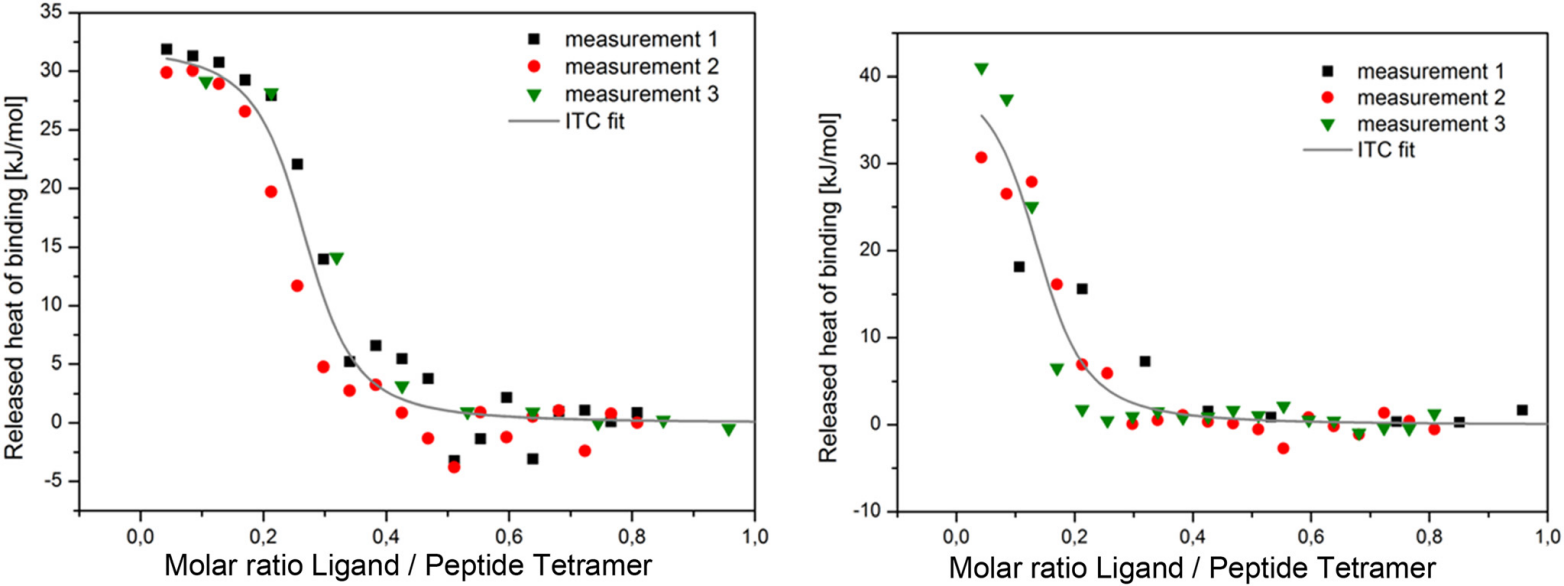
Representative ITC plots for enantiomers 2-*R*, 2-*S*.

Assays using various biophysical methods have been applied to test the binding of drug molecules to the AM2 channel at pH 7.5–8 in dodecyl phosphocholine (DPC) micelles or phospholipid bilayers, where tetramers mainly exist in an AM2 monomer/lipid ratio of ≤1 : 25. The AM2 construct system is titrated using a drug dissolved in the same lipid environment. The developed methods mostly explore the thermodynamics of direct ligand binding to AM2 binding. For example, they have applied circular dichroism (CD) spectroscopy, which monitors the ellipticity ratio at 223 *vs.* 209 nm (*θ*_223_/*θ*_209_),^110^ or analytic ultracentrifugation (AUC),^110^ or isothermal titration calorimetry (ITC) to AM2TM peptides in DPC micelles (Fig. 5).^39,111^

An assay^112^ was developed that uses the quenching of W41 fluorescence by H37 protonation below pH 6 in the AM2FL channel reconstituted in LDAO (*N*,*N*-dimethyldodecylamine-*N*-oxide) detergent. The quenching of W41 fluorescence was reversed by amantadine and analogs, and was used in testing adamantanamines against AM2 WT.^113^ Another assay measured the thiol-disulfide equilibria of AM2TM or AM2FL in phospholipid bilayers by CD^114^ or surface plasmon resonance (SPR).^115^ A series of SPR experiments was used to accurately measure the affinity of amantadine and rimantadine to AM2TM WT embedded in DMPC liposomes. It was found that this class of drugs can bind to AM2 with two different binding dissociation constants (*K*_d_) in the order of 10^−4^ (*K*_d_ = 0.91 μM) and 10^−7^ M (*K*_d_ = 0.40 mM).^115^ It was shown that a high-affinity binding site corresponds to the AM2 ion channel pore-binding site, which is responsible for the pharmacological activity elicited by amantadine drug and its analogs, in agreement with the ssNMR study of amantadine bound to AM2TM WT (PDB ID 2KQT^67^). It was also found that the low-affinity site corresponds to the secondary binding site, which can be attributed to the lipid face of the pore, in agreement with the solution NMR structures of AM2CD WT in micelles (PDB IDs 2RLF^66^ and 2LJC^71^).

In another assay, the proton flux mediated by AM2TM or AM2CD was measured in small liposomes composed of unilamellar 2 : 1 1-palmitoyl-2-oleoyl-*sn-glycero*-3-phosphocholine (POPC)/1-palmitoyl-2-oleoyl-*sn-glycero*-3-phosphoglycine (POPG) vesicles.^39^ An *in vitro* functional assay, which is an indirect liposome dye release assay for viroporin activity, was also reported.^103^ However, the most straightforward way to determine the blocking effect of M2-mediated proton current is electrophysiology with AM2FL expressed in oocytes, published early for amantadine^116^ and further performed for amantadine analogs.^117^ The compounds are tested with a two-electrode voltage-clamp (TEVC) assay using *X. laevis* frog oocytes microinjected with RNA expressing the AM2 protein.^118–120^ Numerous studies have been performed.^62,80,100,101,121–138^ It was shown that drugs can block AM2-mediated proton current when the *k*_off_/*k*_on_ are, correspondingly, slow off/fast on or slow off/slow on, thus leading to favorable *K*_d_ = *k*_off_/*k*_on_ values.^101,105,106,139^ Only when *K*_d_ (TEVC) = *k*_off_/*k*_on_ is small enough, an *in vitro* antiviral activity is observed.^101,106^ If the *k*_off_ is too high, then while the compound can block the M2 channel, it does not inhibit the virus *in vitro*, and this was the case for compound 8 (see Table 2) against AM2 V27A.^101^ The potency of the inhibitors was usually expressed as the inhibition percentage of the AM2 current observed after 2 min. However, studies performed independently with second-generation amantadine-based drugs against AM2 S31N,^106,139^ rimantadine analogs against AM2 WT,^105^ or spiranic adamantanamines against V27A^101^ showed that the 2-minute measurements of the %-AM2 blocking effect (usually applied for testing a large number of compounds^76,81,139–141^) and filtering the most important for subsequent antiviral assays were not efficient. This is due to the presence of compounds with, *e.g.*, very slow on- and slow off-rates for entry (measured as association and dissociation rate constants, *k*_on_ and *k*_off_, respectively), for which characterization of the blocking effect needs more than 2 min, *e.g.*, 5 min. For example, in ref. 105 for a rimantadine analog, only 27% blocking was observed at 2 min against Udorn AM2 WT, but low micromolar EC_50_ against M2 WT viruses. Despite the very slow binding *k*_on_ rate, the ligand exhibited high antiviral potency, which requires, as a biological effect, much longer exposure times than EP experiments. For such compounds, after 2 min or 5 min, the percentage of proton current inhibition was progressively increased. Thus, in ref. 105 it was striking to observe that the ligand against Udorn AM2 WT exhibited 27% blocking at 2 min, 38% at 5 min, and 61% at 10 min. It must be clarified that the measurements at 2, 5, or 10 min correspond to measurements before the establishment of equilibrium due to very slow on- and off-rates for entry, which characterizes bulky ligands, and the period can't be extended considerably due to the difficulty of maintaining oocytes at low pH for extended periods. Hence, the %-blocking/IC_50_ values determined by the TEVC procedure can be significantly lower than those measured with *in vitro* antiviral assays, where the system can reach the equilibrium block state, or compared with *K*_d_ values at equilibrium measured with ITC^105^ or SPR.^115^ ssNMR studies have also been performed to investigate the binding interactions of rimantadine and of amantadine and other adamantanamines with AM2 (22–62) WT.^83,142–146^

**Fig. 6:**
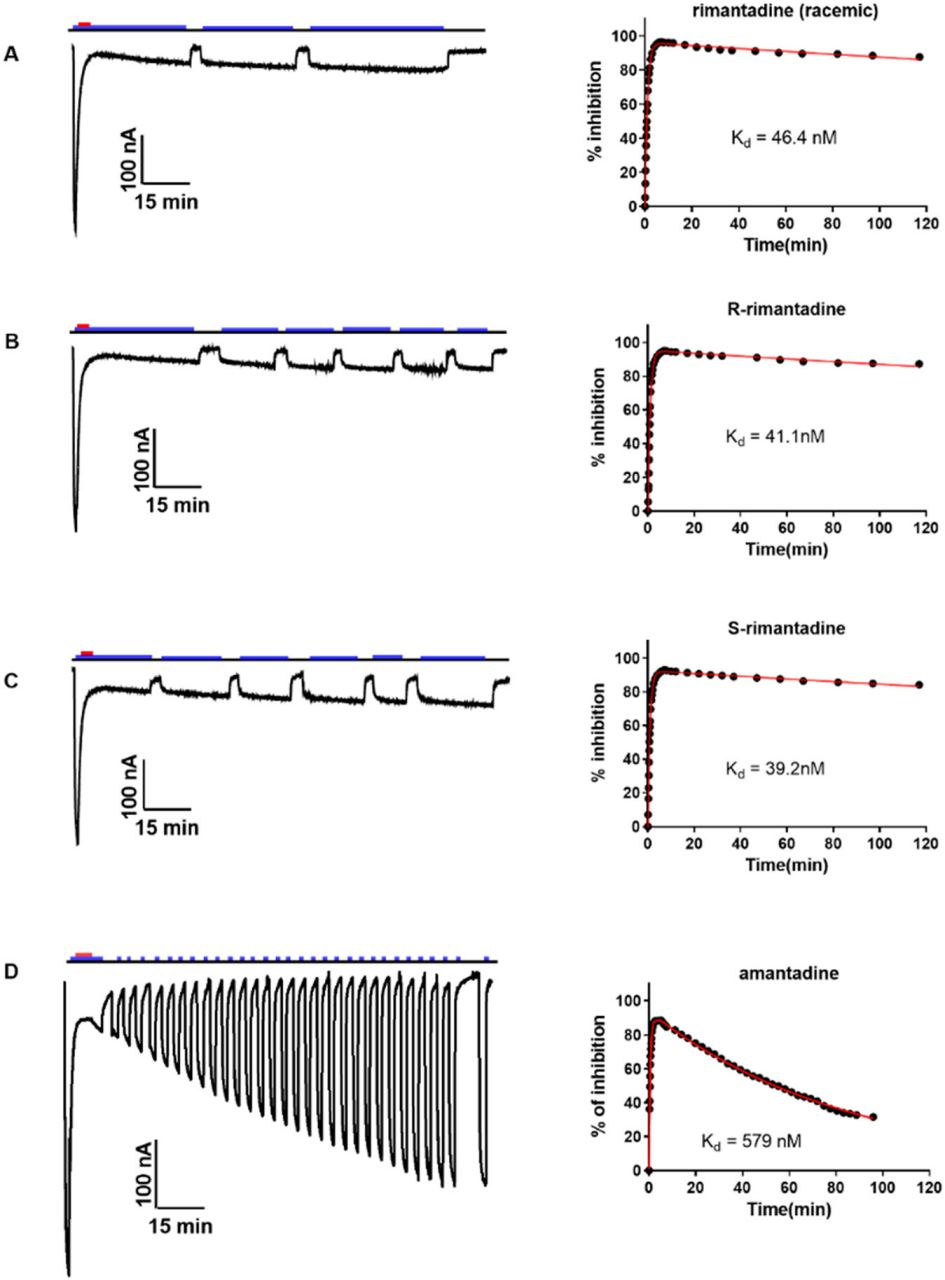
Rimantadine enantiomers and amantadine binding kinetics against Udorn M2 WT were determined using a combined application of inward current and washout procedure TEVC assay.^73^ (A) Racemic rimantadine, (B) (*R*)-rimantadine, (C) (*S*)-rimantadine, or (D) amantadine was applied to oocytes for 5 to 7 min, and after the inward current reached its maximum, a washout protocol was applied to the oocytes. During the washout, pH 8.5, pulses were applied to make sure the current went to baseline to ensure the oocyte quality. The blue bar above the recording trace indicates the period in which the pH 5.5 Barth solution was applied; the red bar indicates the period in which compounds in the pH 5.5 Barth solution were applied. Representative recording traces are shown on the left side of each panel. The best-fit values are shown in Table 1. This figure has been reproduced from ref. 73 with permission from the American Chemical Society, copyright 2021.

An interesting work refers to cell-based antiviral assays and EP, which were applied to evaluate the biological potency of rimantadine enantiomers.^105,147^ It was observed that both enantiomers have similar %-channel blockage, binding *k*_on_ and *k*_off_, *K*_d_, and antiviral potency (EC_50_) against AM2 WT.^105^ These results showed no significant difference between the two rimantadine enantiomers compared to what has been previously suggested with ssNMR at −10 °C, *i.e.*, that the two enantiomers have different affinity against AM2CD WT.^148^ In a subsequent work,^73^ much more accurate binding kinetics were performed compared to previous ones^105^ measurements (Table 1, Fig. 6).

**Table 1:** Summary of the binding affinity of amantadine and rimantadine enantiomers against Udorn M2 WT^73^

	*rac*-Rimantadine	(*R*)-Rimantadine	(*S*)-Rimantadine	Amantadine
Concentration tested	50 μM	50 μM	50 μM	100 μM
*k*_on_ (min^−1^ M^−1^)	19 600 ± 300	20 800 ± 700	22 500 ± 300	20 500 ± 300
*k*_off_ (min^−1^)	(9.1 ± 0.8) × 10^−4^	(9 ± 2) × 10^−4^	(8.8 ± 0.8) × 10^−4^	(119 ± 2) × 10^−4^
*K*_d_ = *k*_off_/*k*_on_ (nM)	46 ± 4	41 ± 9	39 ± 4	580 ± 20

It was concluded that the slight differences in hydration for the (*R*)- and (*S*)-rimantadine enantiomers observed in structures of M2TM WT with PDB ID 6US9^73^ and PDB ID 6US8^73^ are not relevant to drug binding or channel inhibition. Additionally, it did not result in a difference in potency or binding kinetics as was shown by similar values of *k*_on_/*k*_off_, and *K*_d_ for (*R*)- and (*S*)-rimantadine in the TEVC assay (Table 1, Fig. 6).^139,149^

#### Solid-state NMR experiments

Solid-state NMR was applied^144,150^ in AM2CD WT (residues 18–60), in the apo-form or in complex with rimantadine (drug : tetramer ratio ∼ 3 : 1 mol), in DPhPC lipid bilayers at pH 7.8. In this alkaline pH, the His37 tetrad has a neutral charge. Proton resonances at 14.3, 11.7 ppm for the His37 Nδ1, Nε2 of apo-AM2CD WT were recorded in the (H)NH spectra due to His-His^+^ dimer-of-dimers formation (Nδ1–H⋯Nε2 and Nδ1⋯H–Nε2 forms) and showed an intermolecular coupling (*J*_NN_ = 8.9 ± 0.3 Hz) due to hydrogen bonding between Nε and Nδ of adjacent His37.^144,150^ When rimantadine was added to AM2CD WT in DPhPC bilayers or in VM bilayers or in DPhPC-30% cholesterol bilayers at 4 °C, the spectra did not show pore binding at all, as observed previously with AM2CD WT (residues 21–61) reconstituted in VM at 30 °C using a drug : tetramer ratio 5 : 1.^83^ In the presence of rimantadine with AM2CD WT pore at 40 °C, compared to the apo-AM2CD WT signals, was observed intensity reduction of the side chain His37 proton peaks at 14.3 and 11.7 ppm in the (H)NH spectra and the His37-Nε2 shifted upfield, while disruption of the *J*_NN_ hydrogen bond between His37 Nε and Nδ was observed, in accordance with its pore-blocking mode of AM2CD WT inhibition. While binding took place over three days at 40 °C, complete binding was seen in just a few hours at 55 °C, offering a novel NMR assay for the detection of inhibition by adamantanamines. The slow kinetics reported for rimantadine by NMR are opposed to the fast blocking of AM2CD WT (residues 18–60)-induced proton currents by either rimantadine or amantadine observed in liposomal proton flux assays,^116,151^ respectively. At pH 6, however, *i.e.*, around the pH found in the Golgi lumen (pH 6.0–6.7),^152^ where the AM2 channel adopts its activated +3 charge state, the rimantadine pore-binding kinetics were faster.

**Fig. 7:**
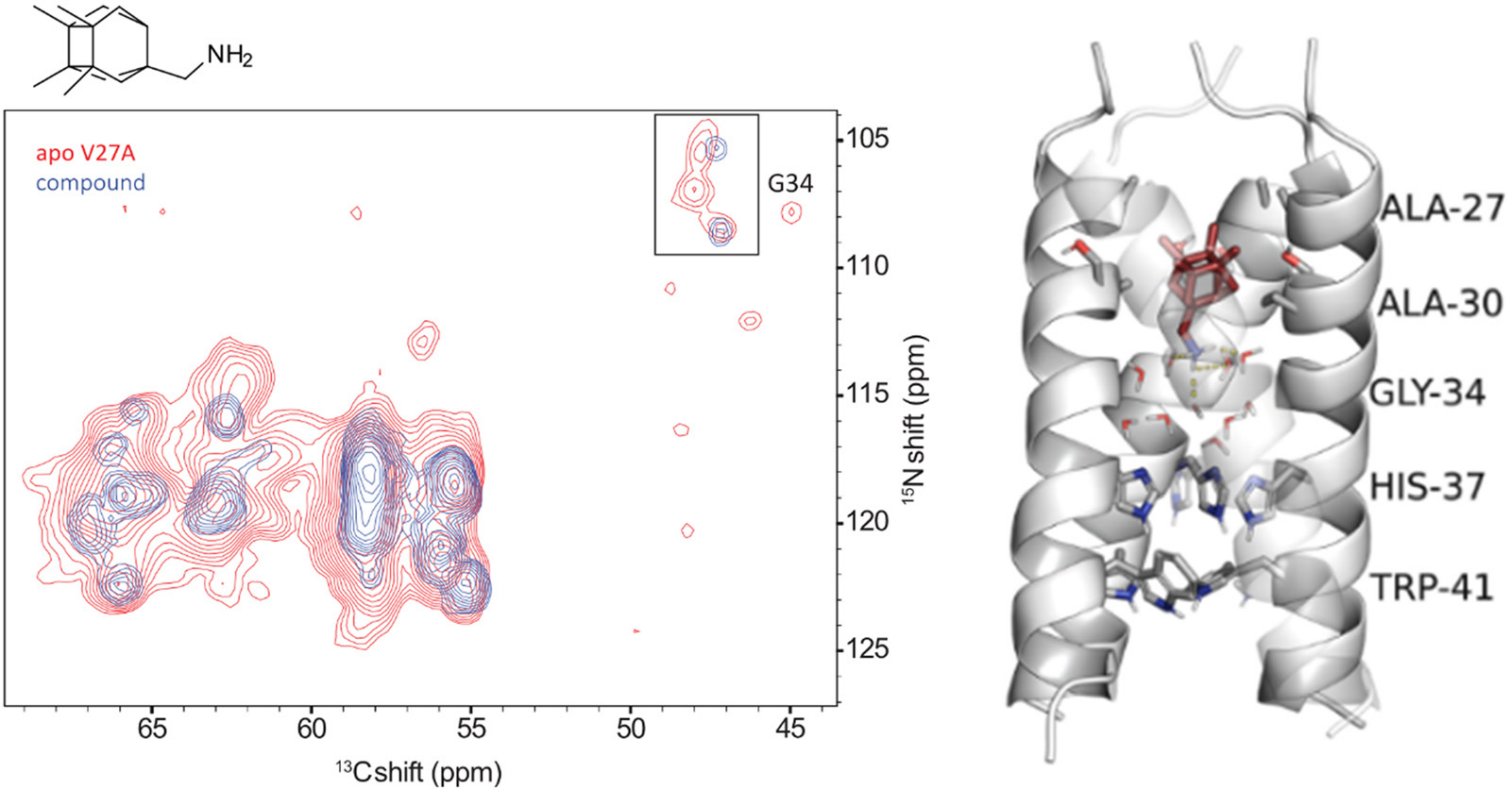
Slices from 3D (H)CαNH spectra (^15^N–^13^Cα projection) of AM2CD V27A and mutant proteins in complex with the cage amine triple blocker (protein was embedded in DPhPC bilayers with a lipid to protein tetramer molar ratio of 24 : 1 at pH 7.8) and results from 500 ns MD simulations for AM2TM-cage amine complexes embedded in POPC bilayers utilizing the CHARMM36m force field.^154^ Panel on the left side: the NMR signals of the apo-protein are shown in red, and the signals of the complexes of AM2CD with the respective compound are shown in blue (at the top left side, in the inset, is shown clearly the pair of signals of G34, which suggests the presence of two conformational states: either due to a slow equilibrium between a kink and non-kink state at G34 or a dimer-of-dimers structure of M2CD). Panel on the right side: average structure of AM2TM V27A from MD simulations; grey cartoon is used to display the AM2TM tetramer, stick representations were used for ligands, waters, and amino acid side chains indicated and cartoon representations was used for the protein backbone (carbons are depicted with grey color for amino acids and ruby color for the ligand, red for oxygen, blue for nitrogen) while the yellow dotted line represents hydrogen bonding between the drug and water molecules. This figure has been adapted from ref. 6 with permission from ELSEVIER, copyright 2025.

3D (H)CαNH NMR was applied to study the potencies against influenza A with the M2 WT channel of several adamantanamines^142^ and some cage amine analogs, and the AM2 L26F, V27A, A30T, G34E mutants. For example, the compound shown in Fig. 7 exhibited a blockage of AM2 WT, L26F, and V27A channels by EP assays, while some adamantanamine analogs blocked AM2 WT and AM2 L26F channels by EP (see structures in Table 2).^153^

Chemical structures of representative adamantanamines or saturated polycyclic amines 1–12 and *in vitro* potencies (from whole cell or TEVC assays) against viruses with AM2 WT or the amantadine-resistant AM2 V27A, AM2 L26F, and AM2 S31N mutant channels

**Table:** 

Virus		Compound			
		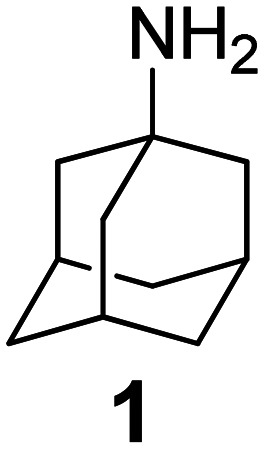	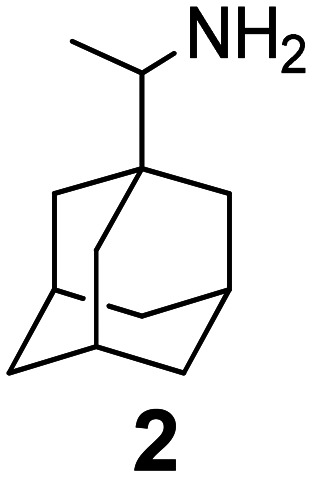	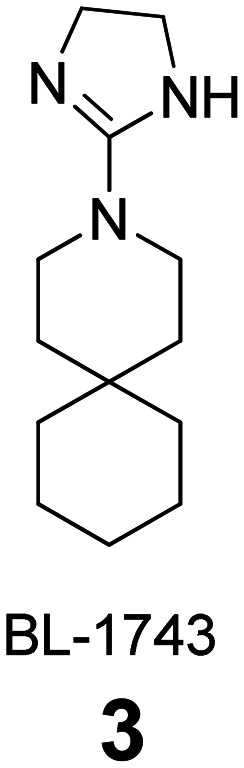	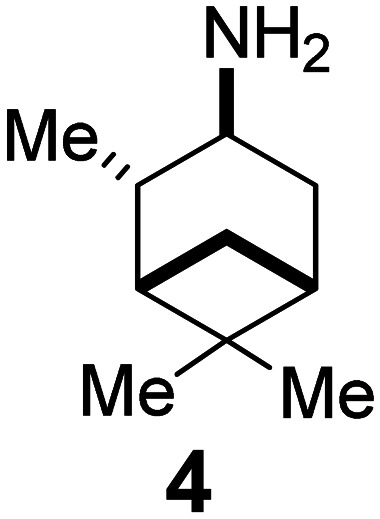
A/M2 WT (S31)	EC_50_ (or IC_50_ in TEVC)	0.78,^a^^,^^105^ 0.48, μM^b^^,^^105^, 5.96,^c^^,^^165^ 12.5 μM,^e^^,^^105^ 8.8 μM^f^^,^^165^	0.48,^a^^,^^105^ 0.04 μM^b^^,^^105^ 10.8 μM,^e^^,^^105^	2 μM^a^^,^^111,117,156^	1.36 μM^c^^,^^165^; 6.8 μM^f^^,^^165^
A/M2 V27A		n.a.	n.a.	n.a.	n.a.
A/M2 S31N		n.a.	n.a.	n.a.	n.a.
A/M2 L26F		n.a.	n.a.	n.a.	n.a.

**Table:** 

Compound		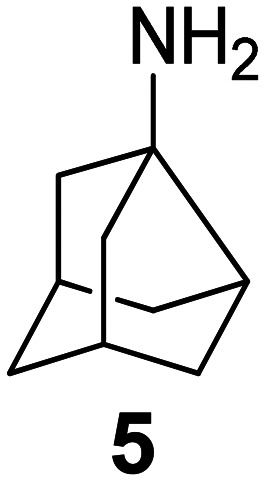	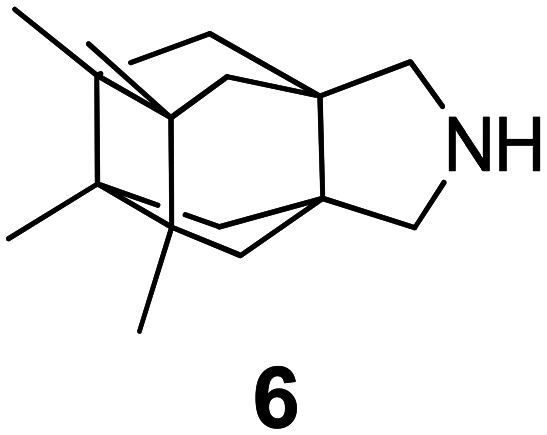	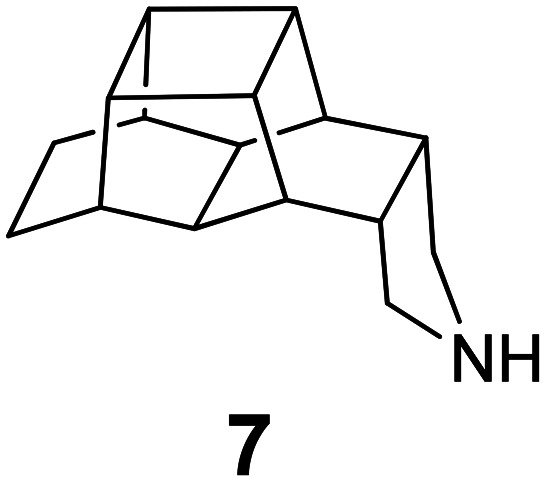	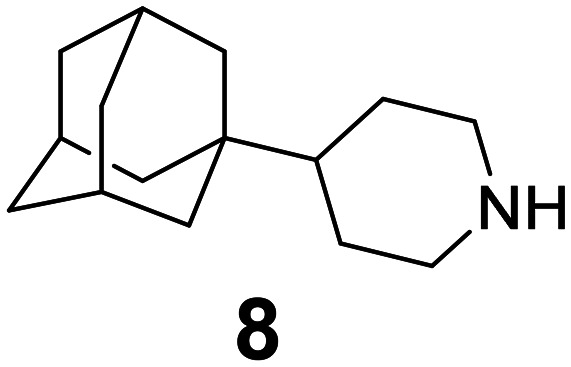
A/M2 S31 (WT)	EC_50_ (or IC_50_ in TEVC)	38.21 μM,^c^^,^^165^ 13.5 μM^f^^,^^165^	18.0 μM^e^^,^^158^	6.7 μM,^d^^,^^166^ 0.54 μM^e^^,^^166^	0.14 μM,^d^^,^^101^ 4.1 μM^f^^,^^101^
A/M2 V27A		n.a.	0.70 μM^e^^,^^158^	17.2 μM^e^^,^^166^	n.a.,^a^^,^^101^ 3.6 μM^e^^,^^101^
A/M2 S31N		n.a.	n.a.	n.a.	n.a.
A/M2 L26F		n.a.	8.6 μM^e^^,^^158^	n.a.	n.a.

**Table:** 

		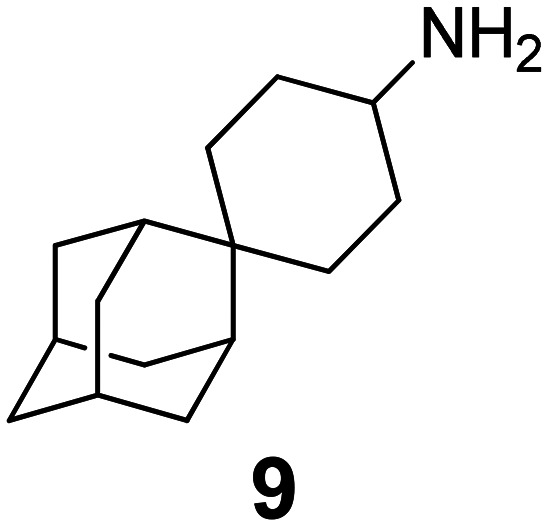	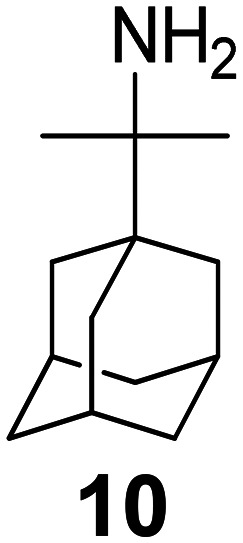	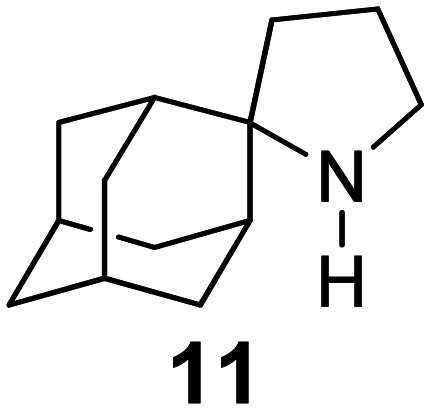	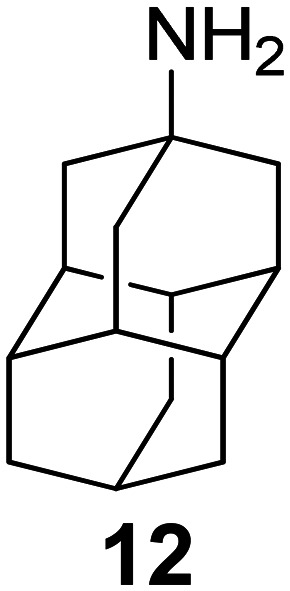
A/M2 S31 (M2 WT)	EC_50_ (or IC_50_ in TEVC)	18.7 μM^a^	0.03 μM,^b^^,^^105^ 0.01 μM,^a^^,^^105^ 0.012 μM,^d^^,^^105^ 9.3 μM^e^^,^^105^	0.34 μM,^b^ 10.2^g^^,^^167^	0.07 μM,^b^ 30.8%-blocking^e^
A/M2 V27A		0.3 μM^a^	n.a.	n.a.	n.a.
A/M2 S31N		n.a.	n.a.	n.a.	n.a.
A/M2 L26F		5.6 μM^a^	0.79 μM^b^ 0.55 μM	8.75 μM^b^	0.74 μM^b^

a Plaque reduction assay against influenza A Udorn virus.

b Cytophathic effect assay against WSN/33 S31 virus.

c Plaque reduction assay against A/Hong Kong/8/68 (H3N2).

d Plaque reduction assay against influenza M2 WT A/HK/7/87 virus.

e Isochronic (2 min) from TEVC studies in oocytes for IC_50_ values.

f Isochronic (2 min) from TEVC studies against M2 from Udorn virus in HEK 293 T cells for IC_50_ values.

g 10.2 μM (3 min) from TEVC studies against M2 from A/California/09/2009 (H1N1) M2: S31 virus; n.a., not active.

The cage amine triple blocker in Fig. 7,^153^ while having length equal to rimantadine, binds and blocks the AM2 V27A channel due to its larger volume, as well as AM2 L26F and AM2 WT, as was shown by 500 ns-MD simulations with the CHARMM36m force field^154^ of AM2CD V27A, AM2CD L26F, and AM2CD WT embedded in a 150 POPC lipid bilayer. This compound's ammonium group was oriented steadily towards the C-terminus in AM2CD WT, V27A, and L26F channels during the MD simulations, and the ligand was stabilized within the AM2 pore by forming an average of three hydrogen bonds with the water that was present between the ligand's ammonium group and His37. Furthermore, by displacing loosely bound waters close to the top of the pore (N-terminal side), the large cage alkyl group stabilized the complex through interactions between the tetramethyl-cyclobutane ring and residue 27 side chains,^44,155^ and successfully blocked the passage of water molecules, preventing protons from entering the pore and generating conduction. The MAS ssNMR provided information on the contacts of the triple blocker in Fig. 7 with AM2CD V27A (residues 18–60), AM2CD L26F, and AM2CD WT. When the drug was added to the mutant channels, there was a noticeable increase in peak resolution as opposed to the relatively wide peaks in the apo-AM2 V27A and apo-AM2 L26F. This suggests that the protein's conformational flexibility was decreased during the formation of the drug–protein complex. Fig. 7 shows some representative results for the AM2CD V27A complex.

### Selected reports of adamantanamines and assays used to measure AM2 inhibition

In Table 2, selected compounds and activities against AM2 WT and its AM2 mutant channels were measured with EP, and the *in vitro* cell-based activities against the corresponding viruses; the activities of amantadine (1) and rimantadine (2), BL-1743 (3),^111,117,156^ pinanamine (4),^157^ and noramantadine 5, which all block the AM2 WT channel by EP and inhibit *in vitro* the replication of the influenza AM2 WT virus.

They also showed the activities of the tetramethyl-3-azapentacyclo[7.2.1.1^5,8^.0^1,5^.0^7,10^] tridecane (6),^158^ which blocks both the AM2 WT- and AM2 V27A-proton mediated current and inhibits *in vitro* AM2 WT, V27A, and L26F viruses. The piperidine derivative 8 blocks the AM2 WT and V27A by EP and inhibits *in vitro* the corresponding viruses.^101^ The azatetracyclo[5.2.1.1^5,8^.0^1,5^]undecane (7)^125^ blocks AM2 V27A by EP, while the spiranic amine 9^159^ and tetramethyl-3-azapentacyclo[7.2.1.1^5,8^.0^1,5^.0^7,10^]tridecane (6)^158^ are blockers of the AM2 WT, V27A, L26F channels and inhibited *in vitro* the corresponding viruses.

The rimantadine analog 10, the spiropyrrolidine[2,2′]adamantane (11), and the diamantanamine 12 block the AM2 WT and AM2 L26F channels by EP.^142^ The adamantanamines and saturated polycyclic amines 1–12 did not block the M2 S31N channel.

These adamantanamines are important agents since they can block AM2 WT, V27A, and L26F viruses. Other lipophilic and saturated polycyclic amines that block these AM2 channels have been reported.^138,160–164^ These compounds can be repurposed if the predominant influenza A M2 S31N, causing the current epidemics, can be changed to one of these channels that are sensitive to adamantanamines.

## Structure-based drug design methods targeting wild-type and mutant AM2 channels

Protein channels and water-filled pores present particularly challenging targets for drug design.^168^ MD simulations for adamantanamine binding to the AM2 protein have been performed, and some of them were applied for structure-based drug design purposes. For example, MD simulations of ligand–AM2TM complexes were used to design and synthesize spiro-piperidine inhibitors (*e.g.*, compound 9 in Table 2) that block the M2 WT, M2 V27A, and M2 L26F channels.^159^ The design of AM2 blockers would benefit from the application of methods and the generation of models that can accurately calculate the relative binding energies of ligands targeting the AM2 channel.

Generally, the molecular mechanics-Poisson–Boltzmann surface area (MM-PBSA) method cannot calculate accurate binding free energies and can only provide a good correlation between calculated and experimental binding free energies for a set of ligands with a *K*_d_ value range of 1000.^169,170^ In contrast, binding free energy methods based on statistical mechanics, *e.g.*, free energy perturbation or thermodynamic integration coupled with MD simulations (FEP/MD or TI/MD, respectively),^171,172^ have proven capable of predicting relative binding free energy with an accuracy of ∼1 kcal mol^−1^ in optimal cases. ITC was used to measure *K*_d_ values of several adamantanamines against the closed state of AM2TM Weybridge strain (with V28I, L38F mutations compared to Udorn) at pH 8; ∼1 : 57 peptide/DPC lipid was used to maintain tetramers.^170^ It was found *K*_d_ = 0.74 μM for amantadine.^170^ It was shown that the application of MM-PBSA provided a fair assessment of the relative importance of the binding free energy components of adamantanamines in their complexes with AM2TM WT after calculating binding free energy decomposition.^173^ In this work, it was revealed that the calculated enthalpy components can be used to prioritize adamantanamines in agreement with their experimental binding affinities measured with ITC.

For a set of rimantadine analogs,^134^ binding affinities were measured using ITC in DPC micelles against the closed state of AM2TM WT and S31N protein channel at pH 8, blocking potency by TEVC against the AM2FL WT and S31N protein channel, and antiviral potency using cell-based assays against virus replication. The rimantadine analogs bear progressively larger alkyl groups (with dimethyl, diethyl, and dipropyl in the carbon bridge, see structures of compounds 10, 13, 14, in Table 4). The results showed that compared to amantadine (1) with *K*_d_ = 2.7 μM by ITC and rimantadine with *K*_d_ ∼ 0.33 μM by ITC, the dimethyl analog 10 had enhanced affinity against AM2TM WT (Udorn sequence) by ITC (*K*_d_ = 0.13 μM), and against AM2FL according to the kinetic binding measurements by EP. This was also confirmed by alchemical relative binding free energy calculations with the FEP/MD method, with OPLS2005 provided with the Desmond software. It was found that the diethyl and dipropyl rimantadine analogs showed reduced affinity and potency (Table 3).^134^

**Table 3:** Measurement of the potency of rimantadine enantiomers 2 and analogs 10, 13, 14, which include progressively larger alkyl groups. ITC measured the binding affinity against AM2TM, the proton blockage against the AM2FL WT and S31N protein channels by EP, and cell-based antiviral potencies (CPE assay)^134^

Complex	ITC	TEVC	CPE
	*K*_d_ (μM)	*k*_on_, *k*_off_ (M^−1^ s^−1^), *K*_d_ (μM)	EC_50_ (μM)
2-*R* + M2 WT	0.32	412, 0.0013, 3.2	0.05
2-*S* + M2 WT	0.34	407, 0.0016, 3.9	0.06
10 + M2 WT	0.13	230, 0.003, 13	0.01
13 + M2 WT	4.59	—	0.46
14 + M2 WT	3.43	34, 0.003, 13 μM	1.07

**Table 4:** Binding constant, free energy, enthalpy, and entropy of binding derived from ITC measurements for AM2TM WT

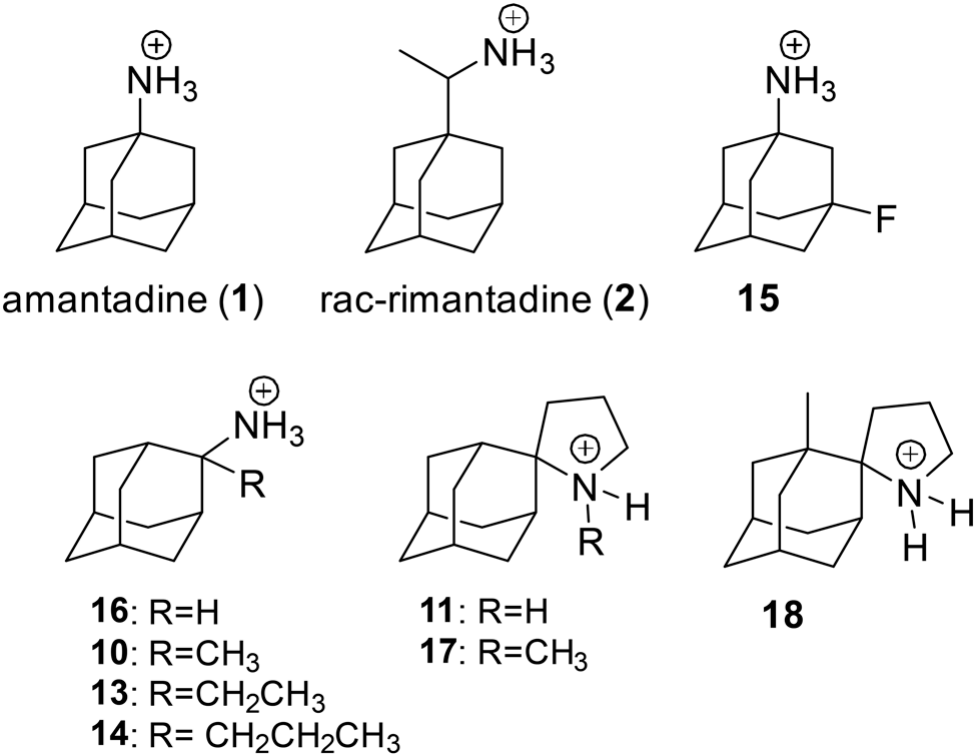				
Ligand	*K*_d_	Δ*G*	Δ*H*	*T*Δ*S*
1	2.17 ± 0.52	−7.77 ± 0.14	−6.66 ± 0.50	1.11 ± 0.52
2^a^	0.51 ± 0.26	−8.64 ± 0.30	−7.60 ± 0.28	1.04 ± 0.41
2-*R*	0.34 ± 0.12	−8.88 ± 0.21	−7.73 ± 0.28	1.15 ± 0.35
2-*S*	0.32 ± 0.16	−8.97 ± 0.26	−7.54 ± 0.34	1.42 ± 0.43
15	6.33 ± 1.53	−7.14 ± 0.14	−3.60 ± 0.31	3.54 ± 0.34
16	1.60 ± 0.34	−7.96 ± 0.13	−7.03 ± 0.42	0.93 ± 0.44
10	0.89 ± 0.19	−8.31 ± 0.13	−6.79 ± 0.26	1.51 ± 0.29
13	0.62 ± 0.14	−8.52 ± 0.13	−7.14 ± 0.21	1.38 ± 0.25
14	0.63 ± 0.17	−8.53 ± 0.16	−7.62 ± 0.30	0.91 ± 0.34
11	0.36 ± 0.22	−8.90 ± 0.43	−5.02 ± 0.41	3.88 ± 0.60
17^b^	0.93 ± 0.36	−8.28 ± 0.23	−3.82 ± 0.28	4.46 ± 0.36
18^a^	1.30 ± 0.43	−8.08 ± 0.20	−5.08 ± 0.31	3.00 ± 0.37

a Racemic mixture.

b Racemic mixtures resulted from the protonation of *N*-methyl spiro[pyrrolidine-2,2′-adamantane].

Similarly, the antiviral potency (EC_50_) evaluation showed the same ranking. In ref. 137 was also shown that the calculated relative binding free energies with FEP/MD calculations was ΔΔ*G*_FEP/MD_ (3 → 2**-*R***) = 0.62 ± 0.14 kcal mol^−1^ with ΔΔ*G*_ITC_ (3 → 2**-*R***) = 0.33 ± 0.50 kcal mol^−1^, while ΔΔ*G*_FEP/MD_ (3 → 2**-*S***) = 0.68 ± 0.15 kcal mol^−1^ with ΔΔ*G*_ITC_ (3 → 2**-*R***) = 0.42 ± 0.48 kcal mol^−1^. These FEP/MD calculation results supported that rimantadine enantiomers, although chiral molecules, have equal binding affinities against a chiral receptor, *i.e.*, the AM2TM WT protein. The MD simulations in ref. 134 showed that in diethyl and propyl analogs (13 and 14, respectively, see Table 3), the alkyl groups appear to better occupy the space between the ligand and the pore walls. For these compounds' binding, the ITC data indicated that the constrained motion and the resulting entropy cost of binding are important quantities and reduced the binding affinities of 13 and 14 compared to 10 (see Table 1 in ref. 134). The proton blocking activity of the adamantanamines is consistent with the fact that no water molecules were detected in the area above the adamantane core (*i.e.*, toward the N-terminus) in the MD simulations.

A few adamantanamines (1, 2-*R*, 2-*S*, 3–10; Table 4) in complex with AM2TM WT were studied at acidic pH^174^ and alkaline pH^175^ with alchemical calculations using the FEP/MD method with OPLS2005 force field provided with the Desmond software, and relative free energies were calculated using the Bennett acceptance ratio (BAR).^171,172^ The *K*_d_ values were used as experimental probes for the FEP/MD calculations of alchemical relative binding free energies.

While the set of 12 compounds in Table 4 had a very narrow range of binding free energies of 8 kJ mol^−1^ (1.9 kcal mol^−1^), *i.e.*, *K*_d_ values for the close state of AM2TM WT (Udorn strain) at pH 8 that differed by a factor of 20 (Table 4), the FEP/MD method performed with high correlation found (*r* = 0.94, *p* < 0.001, PI = 0.74) between calculated and experimental binding free energies.^175^ The correlation was also good in FEP/MD simulations of AM2TM at acidic pH using experimental *K*_d_ values measured at acidic pH against the open conformation of AM2FL.^119^ As mentioned previously, the experimental assay was based on inhibition by adamantanamines of the quenching of W41 fluorescence by H37 protonation below pH 6 in AM2FL in a detergent environment.^118^

**Fig. 8:**
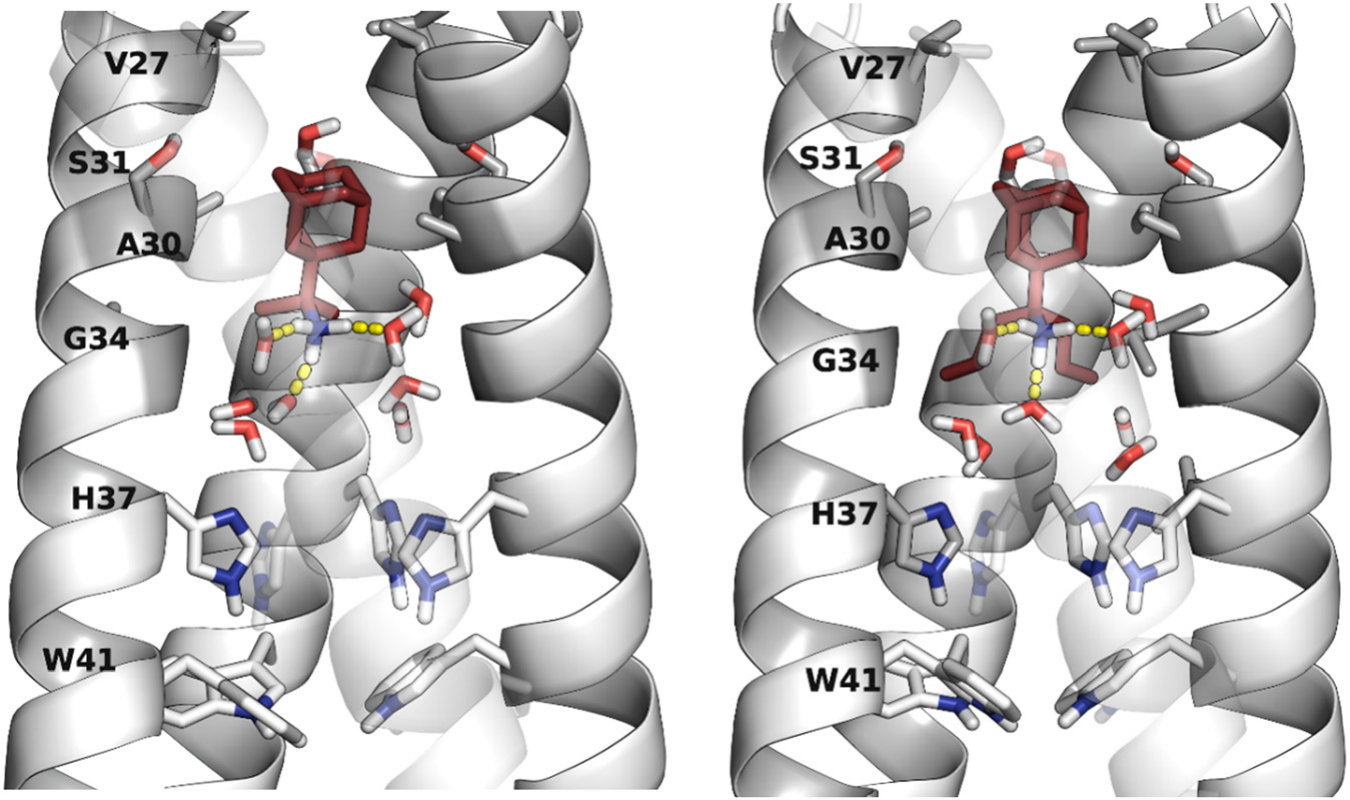
Representative structures from MD simulations with ff19sb^176^ of compounds (*R*)-rimantadine, its dimethyl and dipropyl adduct (see compounds 10, 14 in Table 4) in complex with AM2TM WT in DMPC bilayers. In the figure, the protein is shown with a white cartoon and the ligands with sticks and red color. The water molecules are shown with sticks. White is used for hydrogen, red for oxygen, and blue for nitrogen; yellow dotted lines show the hydrogen bonds.

Subsequently, the experimental binding free energies computed based on the *K*_d_ values measured with ITC were measured against the closed form of AM2TM tetramer in DPC micelles for a larger set of 27 adamantanamines, and the antiviral potencies (IC_50_) with whole cell assays were also measured.^169^ The range of the experimental *K*_d_ values was ∼44, and the range of the antiviral potencies (IC_50_) values was ∼750. A good correlation (*r* = 0.76) was found between their corresponding binding free energy, computed using *K*_d_ or IC_50_ values. MD simulations with Amber19sb force field (ff19sb)^176^ or CHARMM36m force field^154^ and different experimental starting structures of AM2TM were used to investigate the binding mode of adamantanamines that bind strongly, moderately, or tightly to AM2TM embedded in DMPC, DPPC (dipalmitoyl-*sn-glycero*-3-phosphocholine), POPC, or a virus (VM) membrane (Fig. 8).

FEP/MD NPT simulations with the OPLS2005 force field provided with the Desmond software and the BAR estimator,^171,172^ TI/MD NVT simulations with ff19sb^176^ and the multistate BAR (MBAR) estimator^177^ were applied for the AM2TM WT–adamantanamine complexes embedded in DMPC, DPPC, and POPC, to precisely predict experimental relative binding free energies measured with ITC. Dual topology and single topology alchemical transformations were used with TI/MD and FEP/MD, respectively. The pair of ligands considered bears subtle changes in the ligands' structures. With all lipids, it was found that both approaches exhibited a very good correlation between the calculated and experimental relative binding free energies (*r* = 0.77–0.87, MUE = 0.36–0.92 kcal mol^−1^), with FEP/BAR performing the worst in DMPC bilayers and TI/MBAR performing the best. The experimental binding free energies were also calculated using antiviral potencies, compared to *K*_d_ values, and both FEP/BAR (*r* = 0.83, MUE = 0.75 kcal mol^−1^) and TI/MBAR (*r* = 0.69, MUE = 0.77 kcal mol^−1^) performed well also in this case.^169^

It should be noted that FEP/MD accuracy depends heavily on force fields and sampling, and that these methods are not a substitute for experimental validation, especially given the complexities of protonation states and water networks in AM2.

## Synthetic routes to selected adamantane-based and saturated polycyclic amines targeting AM2 channels

### General

Adamantane derivatives have been extensively used in medicinal chemistry and can be found in several drugs.^19,178–181^ After the original publication of amantadine's antiviral activity by du Pont de Nemours in 1964,^182^ early work in the synthesis of amantadine analogs was reported by the same company in 1970 and 1971^183,184^ and Philips-Duphar Research Laboratories in 1971.^185,186^ One of the most significant contributions was the synthesis of over 300 compounds from a lab in Greece, synthesized between 1994 and 2003, see, for example, ref. 113, 137, 142, 145 and 187–199. Most were tested initially for their *in vitro* potency against influenza A. We mentioned here a few works, including selected synthesis of adamantanamines, saturated polycyclic amines, including pinanamine or camphor analogs.^111,134,146,153,159,160,162,163,200–208^ We started with the presentation of the simpler adamantanamines synthesized with a more scalable route, followed by bulkier cage alkyl amines and saturated polycyclic amines to explore modification of the common adamantane ring. We presented in a distinct section the second-generation AM2 S31N inhibitors that can become available through simple synthetic procedures. Elegant syntheses, including radical functionalization methods, have been developed to access various substituted adamantanes, 1,2-didubstituted adamantanes, and diamondoids.^209,210^ Selected syntheses of various adamantanamines and other adamantane derivatives have been reviewed.^18,19^

### Rimantadine analogs

Extensive structure–activity relationships (SARs) about the *in vitro* potency have been performed for over 40 years, as mentioned previously in numerous publications, see for example, ref. 208 and 211. These references include selected examples from the literature regarding the synthesis of amantadine and rimantadine analogs, which have been tested and found potent against influenza A. Adamantane derivatives have been extensively used in medicinal chemistry and can be found in several drugs.^19,178–181^ Elegant syntheses, including radical functionalization methods, have been developed to access various substituted adamantanes, 1,2-didubstituted adamantanes, and diamondoids.^209,210^ Nevertheless, selected syntheses of various adamantane derivatives have been recently reviewed in ref. 19.

**Scheme 2:**
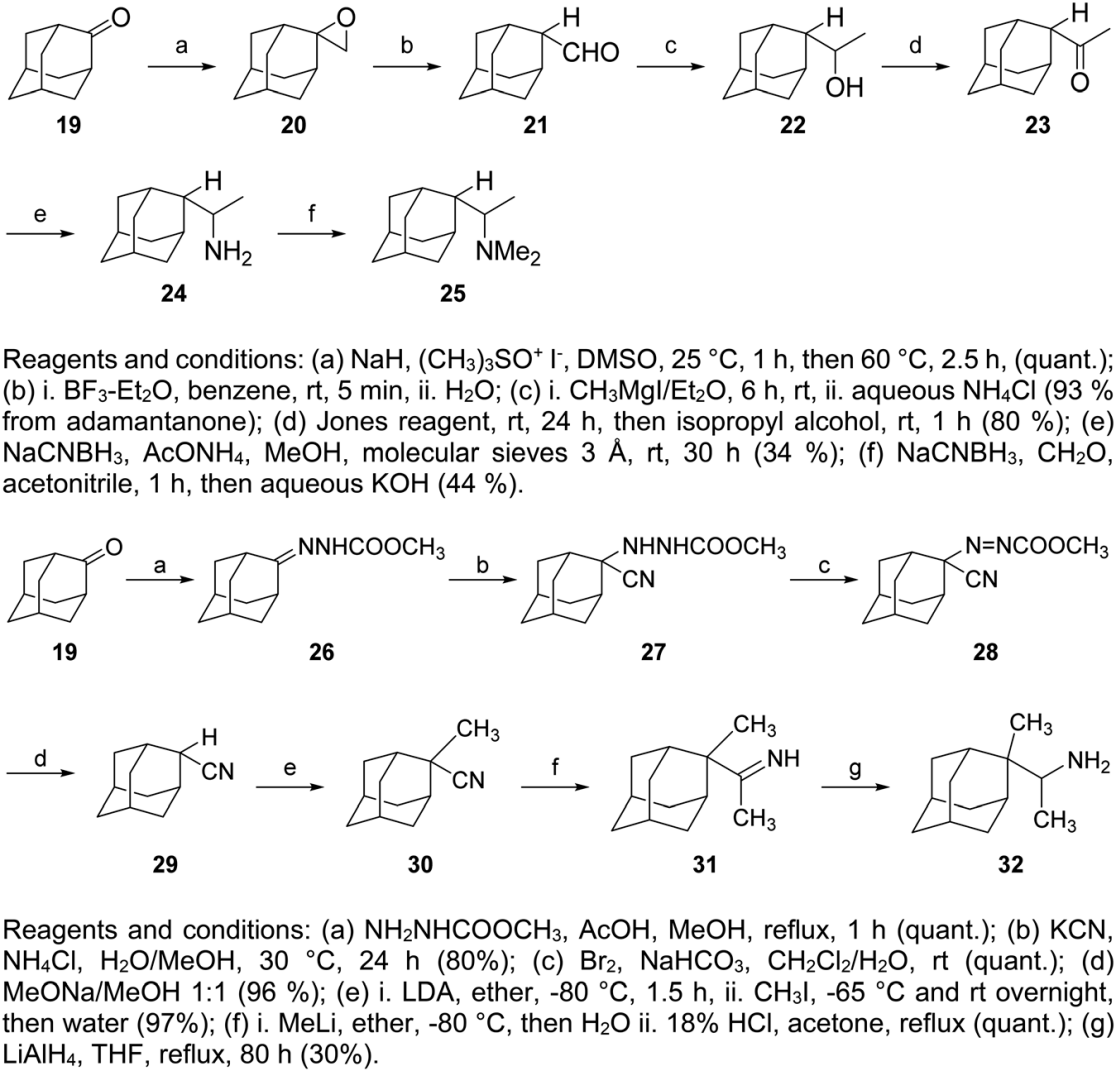
Synthesis of acyclic 2-rimantadine analogs 24, 25, 32.

For the preparation of 2-rimantadine analogs 24, 25, 32, key intermediates were the 2-adamantyl methyl ketone 19 or the 2-cyanoadamantane 29, as is described in Scheme 2.^212^

**Scheme 3:**
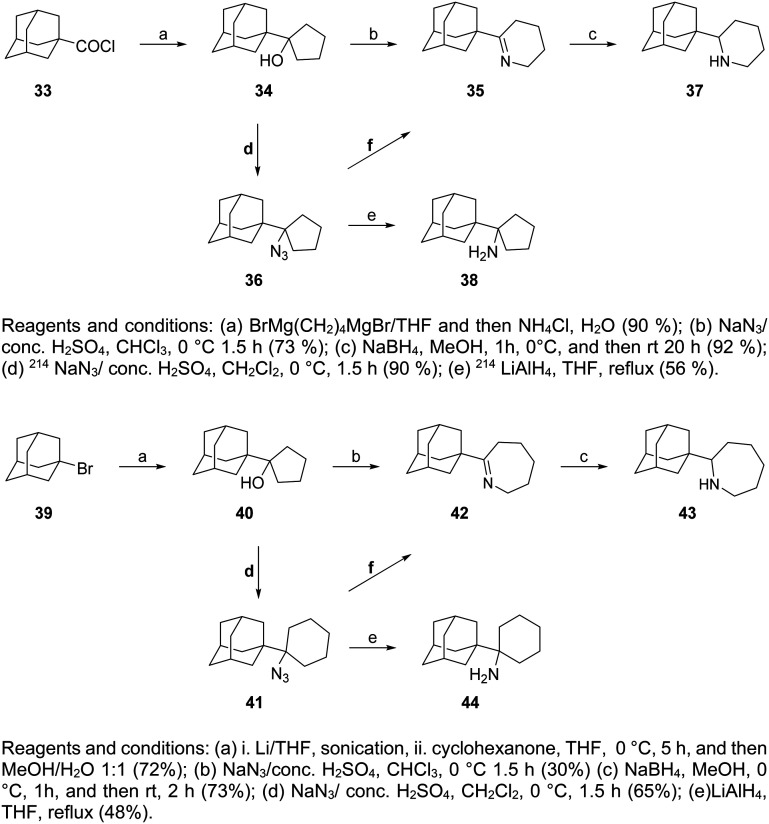
Synthesis of cyclic rimantadine analogs 37, 38, 43, 44.

The cyclic rimantadine analogs 37, 38, and 43, 44 (Scheme 3) were also synthesized with key intermediates the cyclic alcohols 34 and 40, obtained from the reaction of the acid chloride 33 with the dimagnesium reagent BrMg(CH_2_)_4_MgBr^167,213,214^ and with 1-adamantyl-lithium (generated from 1-bromoadamantane 39 with lithium) with cyclohexanone, respectively.^18,215^ Primary *tert*-alkyl amines 38, 44 were obtained from azides 36, 41, respectively, and a work was published that provided routes to produce primary *tert*-alkyl amines from corresponding alcohols, improving steps d for the preparation of 36 from 41.^214^

**Scheme 4:**
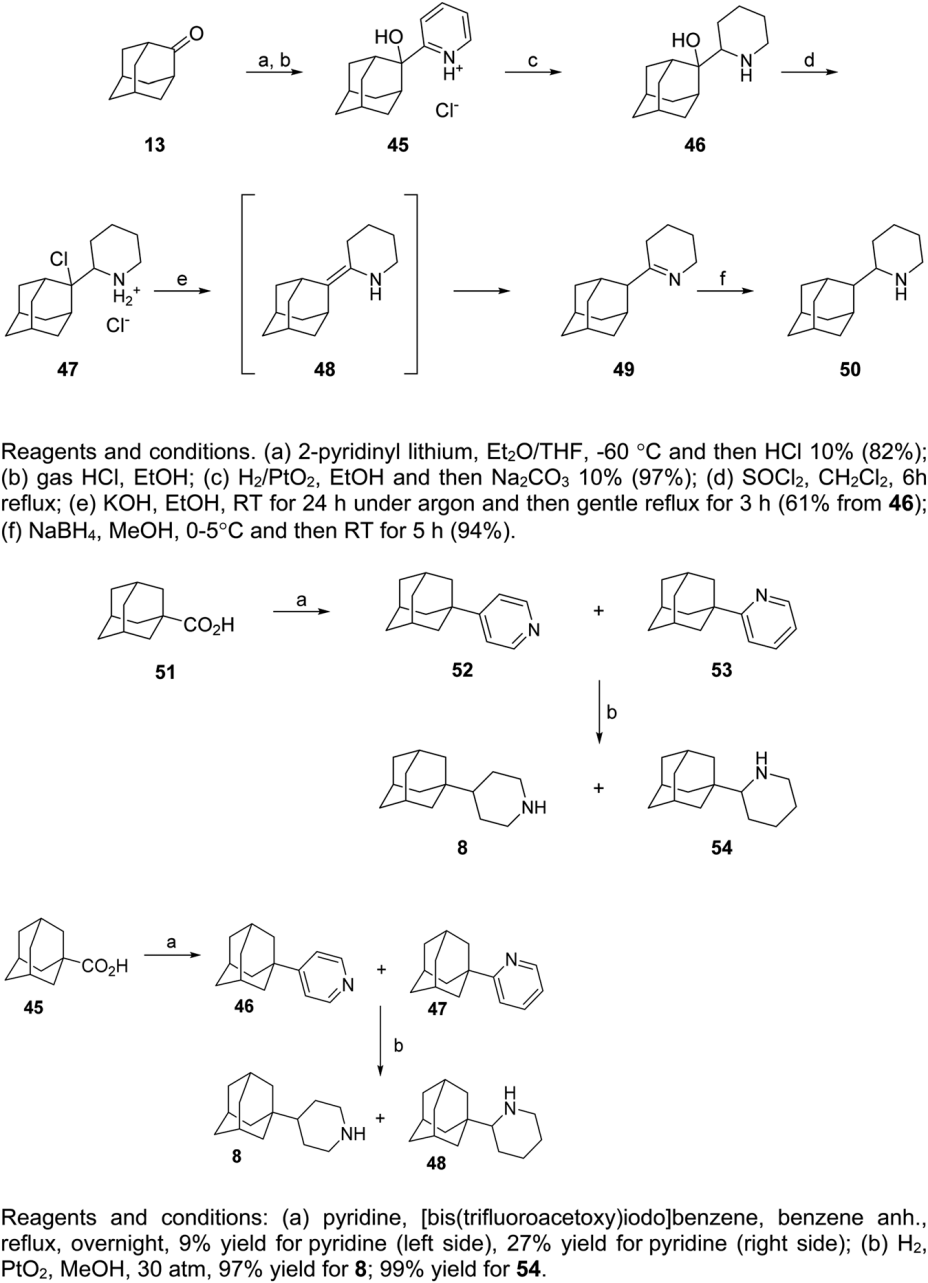
Synthesis of cyclic rimantadine analogs 8 and analogs 48, 50, 54.

Important intermediates for the preparation of 50 were the aminoalcohols 45 and 46 (Scheme 4). Compound 45 was obtained from the reaction of 2-pyridinyl lithium with 2-adamantanone 13, and its protonated form was subjected to catalytic hydrogenation over PtO_2_ catalyst to provide compound 46.^216^ Compounds 8 and 54 were prepared from adamantane carboxylic acid 51 by treatment with [bis(trifluoroacetoxy)iodo]benzene, followed by catalytic hydrogenation over H_2_, PtO_2_ (Scheme 4).

### Spiranic adamantanamines

Synthesis of BL-1743 (3) was accomplished with the reduction of commercially available 3,3-pentamethylene glutarimide (55) with LiAlH_4_ in refluxing THF to give 3-azaspiro[5,5]undecane (51), which was subjected to a nucleophilic substitution with 2-methylthio-2-imidazoline to furnish BL-1743 (3).

The rigidity and crowding around the 2-position of the adamantane nucleus often make chemical transformations difficult and the synthesis of rigid adamantane derivatives challenging. A few synthetic pathways are described below.

**Scheme 5:**
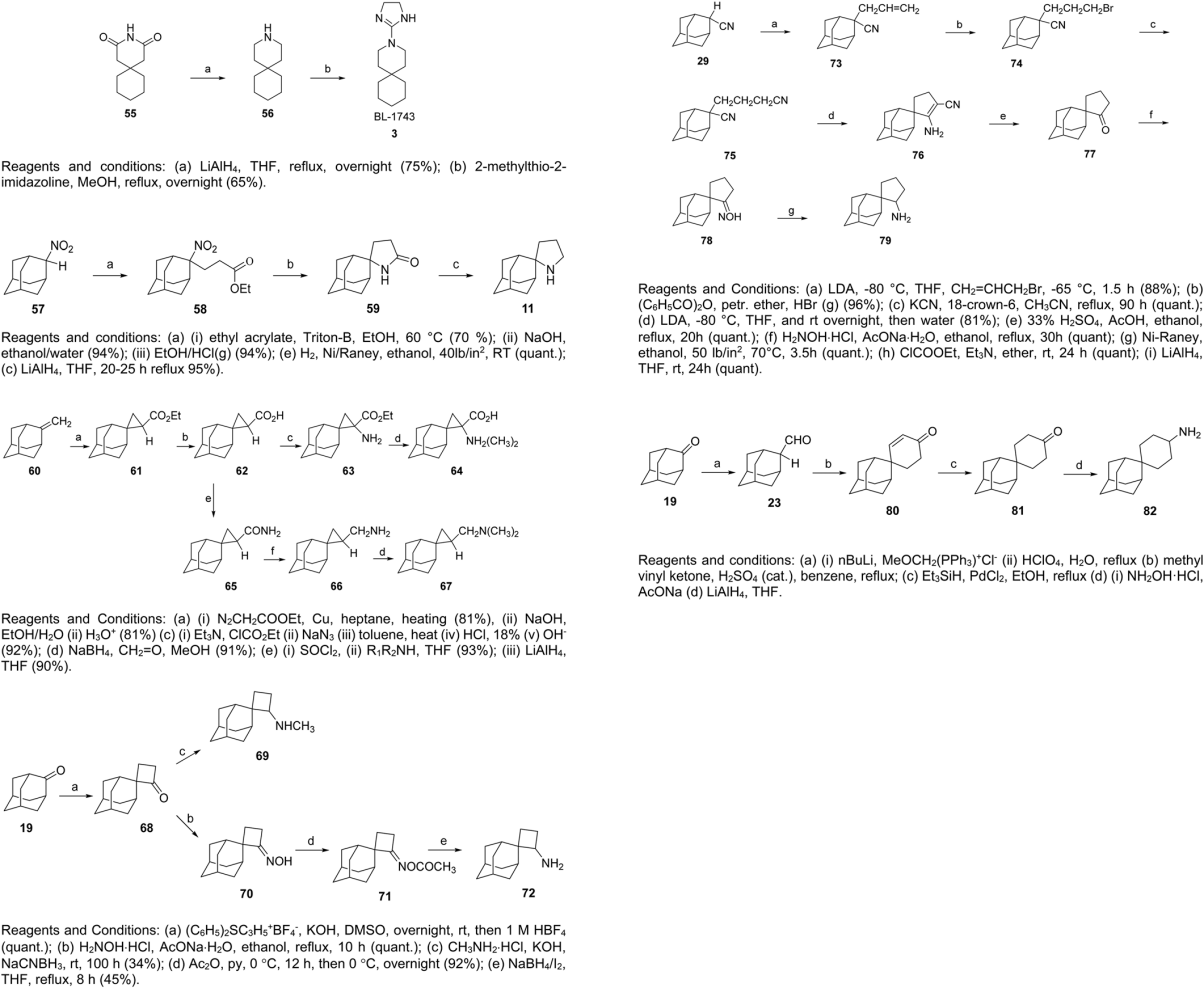
Synthesis of 3-azaspiro[5,5]undecane (5) and its 4,5-dihydro-1*H*-imidazol-2-yl derivative (BL-1743; 3), spiropyrrolidine 11, spiro[cyclopropane-1,2′-adamantan]-2-amines 63, 64 and -methanamines 66, 67, spiro[cyclobutane-2,2′-adamantan]-2-amine 73, 69 and spiro[cyclopentane-3,2′-adamantan]-2-amine 79, and its and spiro[adamantane-2,1′-cyclohexan]-4′-amine (82).

2-Nitroadamantane 57, which was prepared originally as described in ref. 131, was used for the synthesis of spiropyrrolidine 11; the latter was prepared through a Michael condensation of 57 with ethyl acrylate to afford nitroester 58, which was hydrogenated over Ni to afford spiro[pyrrolidine-2,2′-adamantan]-5-one (59) that was reduced with LiALH_4_ to compound 11^217,218^ (Scheme 5).

The key intermediate for the synthesis of the spiro[cyclopropane-1,2′-adamantan]-2-amines 63, 64 and methanamines 66, 67 (Scheme 5) was ethyl spiro[cyclopropane-1,2′-adamantane]-2-carboxylate 61, obtained from the [2 + 1]cycloaddition reaction of ethyl diazoacetate with 2-methyleneadamantane 60 in the presence of copper-bronze and purified through saponification and conversion to acid 62. Routine transformations were performed to prepare 63, 64, 66, 67.

The synthesis of spiro[cyclobutane-2,2′-adamantan]-2-amines 69 or 72 and spiro[cyclopentane-3,2′-adamantan]-2-amines 79 was also accomplished. For the synthesis of these amines, the spirocyclobutanone 68 or spirocyclopentanone 77 were used, respectively, the latter obtained from the dinitrile 75, which was transformed to the cyanoenamine 76 through the Thorpe–Ziegler reaction (Scheme 5).^212^ Key reaction for the synthesis of cyclohexanamine 82 was a Diels–Alder reaction between 2-adamantanecarboxaldehyde and methyl vinyl ketone to afford cyclohexenone 80.

### 1,2-Annulated heterocycles

**Scheme 6:**
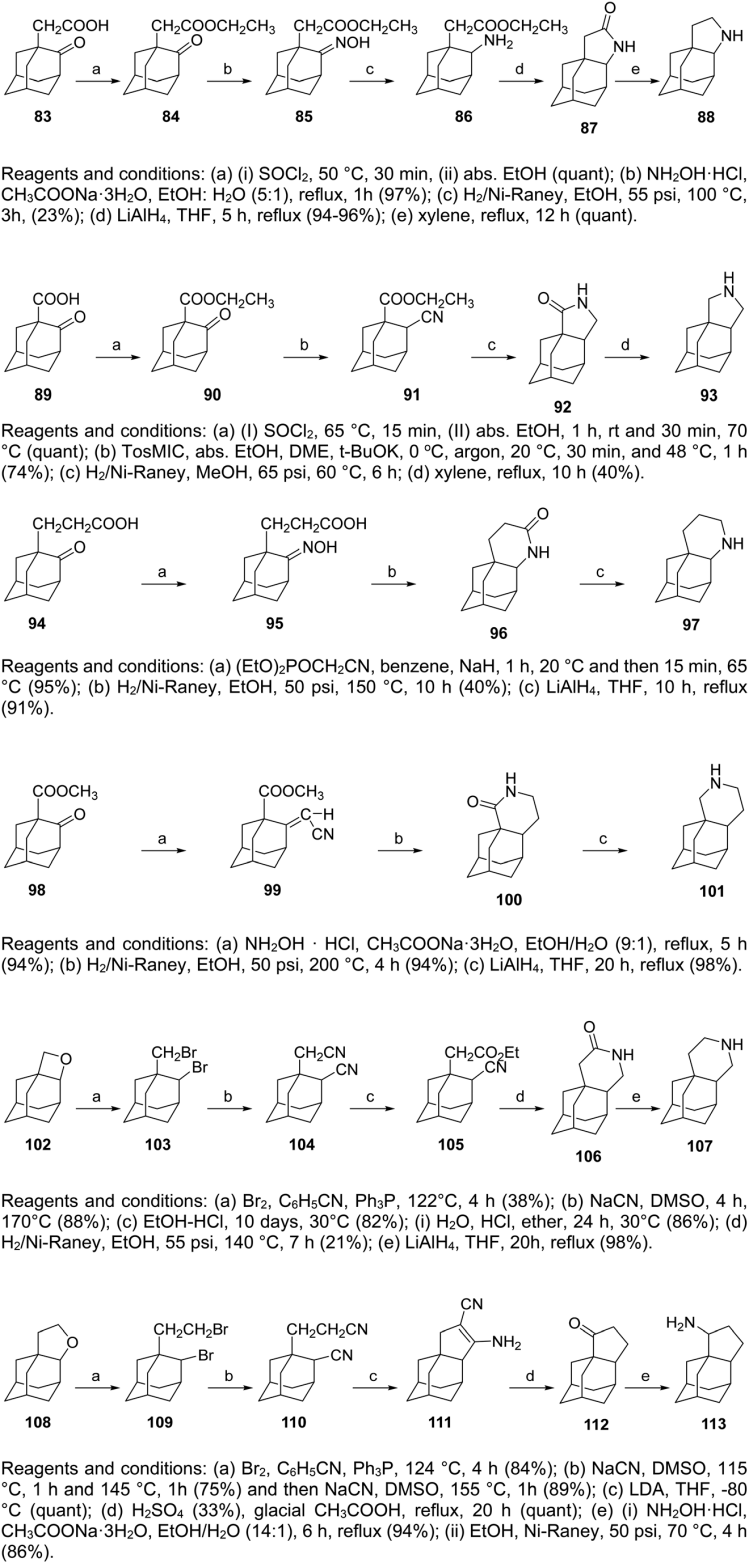
Synthetic procedures for the preparation of the 1,2-annulated adamantanepyrroldines and piperidines 88 or 93 and 97, 101, 107, and 113.

So far, we have presented adamantane derivatives with substitutions at the 1- or 2- position of the adamantane ring. Here are examples of 1,2-annulated adamantano-pyrrolidines and adamantano-piperidines that have been synthesized but not yet tested against influenza A strains. These synthetic ways show how to access disubstituted adamantane derivatives, which might be important for drug design, as different substitutions of the adamantane cage can affect the potency of M2 channel blockers. Synthetic approaches towards the 1,2-substitution pattern on the adamantane framework have been presented.^210,219^ Selected works containing a directed C–H functionalization step have been reviewed in ref. 210. Additionally, the synthesis of various 1,2-annulated derivatives with antiviral potency has been reviewed.^220^ As illustrated in Scheme 6, the 2-oxo-1-adamantane acetic acid 83 or 2-oxo-1-adamantane carboxylic acid 89 or 2-oxo-1-adamantanepropanoic acid 94 were the key starting materials to afford the 1,2-annulated adamantano-pyrrolidines 88 or 93 or the adamantano-piperidine 97 or 101 (Scheme 6). For the synthesis of 107, the oxetane derivative 102 first reacted with the triphenyldibromophosphorane, which was prepared *in situ* by the addition of Br_2_ to a solution of triphenylphosphine. The resulting dibromide derivative 103 was treated with sodium cyanide in DMSO to obtain the dinitrile 104, which was then converted to lactam 106 and amine 107 (Scheme 6).

The synthesis of cyclopentanamine 113 in Scheme 6 starts from the tetrahydrofuran 108, which was converted to dinitrile 110. By employment of the Thorpe–Ziegler reaction, dinitrile 110 underwent an intramolecular condensation catalyzed by LDA to form the enamine 111. Subsequent acid-promoted hydrolysis of the latter gave rise to the racemic cyclic ketone 112, which was converted to the corresponding oxime, which was hydrogenated over RANEY® Ni to provide the cyclopentanamine 113.

### Saturated polycyclic amines

**Scheme 7:**
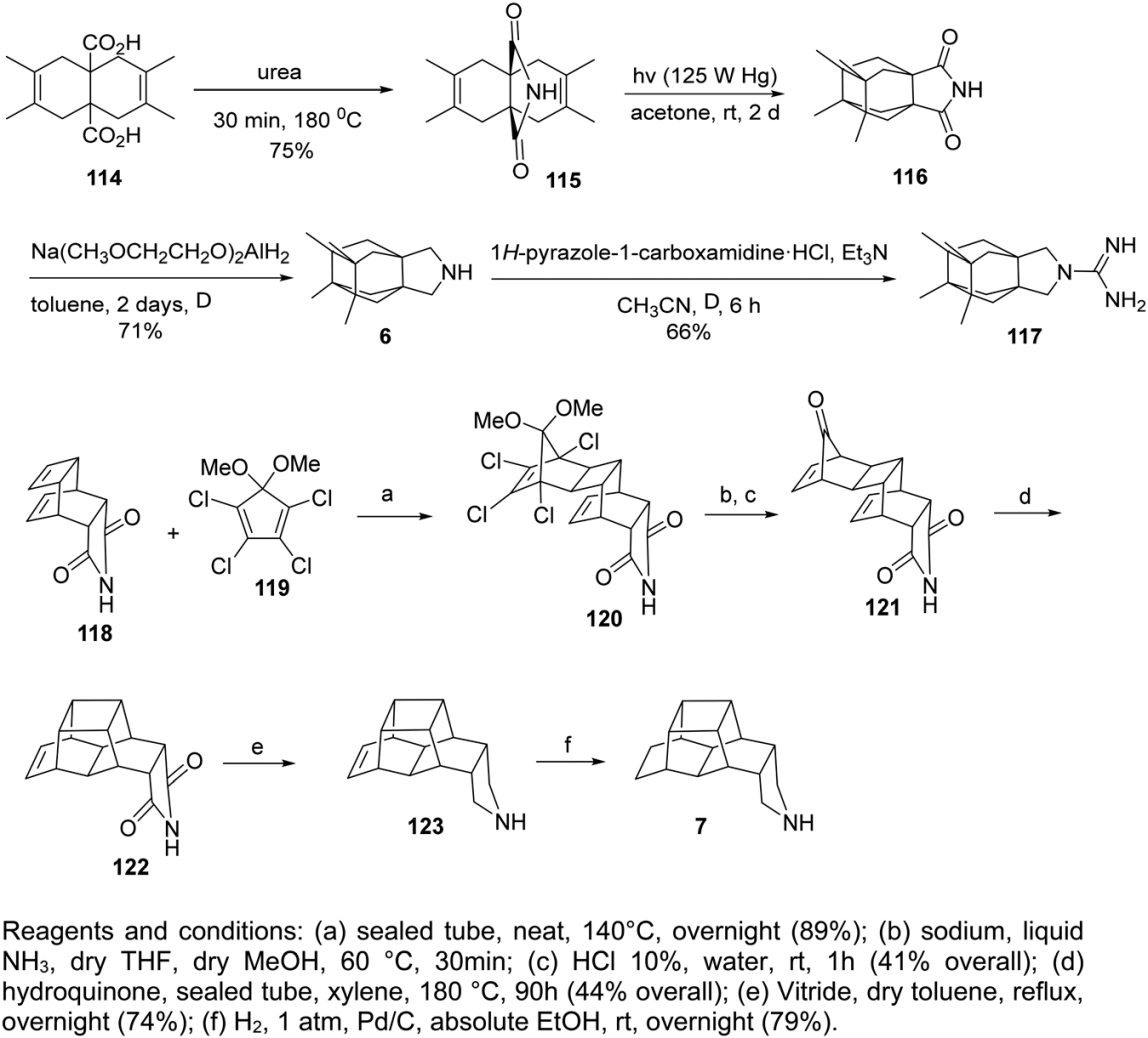
Synthetic procedure for the preparation of the polycyclic amines 6, 7.

The saturated polycyclic amines, *e.g.*, 7,8,9,10-tetramethyl-3-azapentacyclo[7.2.1.1^5,8^.0^1,5^.0^7,10^]tridecane (6)^158^ and heptacyclo[8.6.1.0^2,5^.0^3,11^.0^4,9^.0^6,17^.0^12,16^]heptadecane (7)^166^ (Table 2) were potent triple AM2 WT, L26F, V27A blockers and inhibitors *in vitro* of the corresponding viruses. For their synthesis, fascinating reaction sequences were applied (Scheme 7).

For the synthesis of the aesthetically appealing amines 6 that include one four-membered ring, three five-membered rings, and two six-membered rings, the known diacid 114 was used, easily available from the cycloaddition reaction between acetylene dicarboxylic acid and 2,3-dimethylbutadiene. The reaction of 114 with urea at 180 °C for 30 min yielded the corresponding imide, which was subjected to photolysis at rt in acetone with a 125 W Hg lamp for 2 days to furnish tetracyclic imide 116, which was reduced with sodium bis(2-methoxyethoxy)aluminum hydride to amine 6.^158^

The starting material for the synthesis of 7 was the imide 118, prepared from maleimide with cycloheptatriene. The Diels–Alder reaction between 118 and 5,5-dimethoxy-1,2,3,4-tetrachlorocyclopentadiene furnished 120. Then, a one-pot dechlorination/acetal deprotection reaction with metallic sodium in liquid ammonia/acidic medium, and a one-pot decarbonylation produced 121 from which 7 was furnished.^166^

**Scheme 8:**
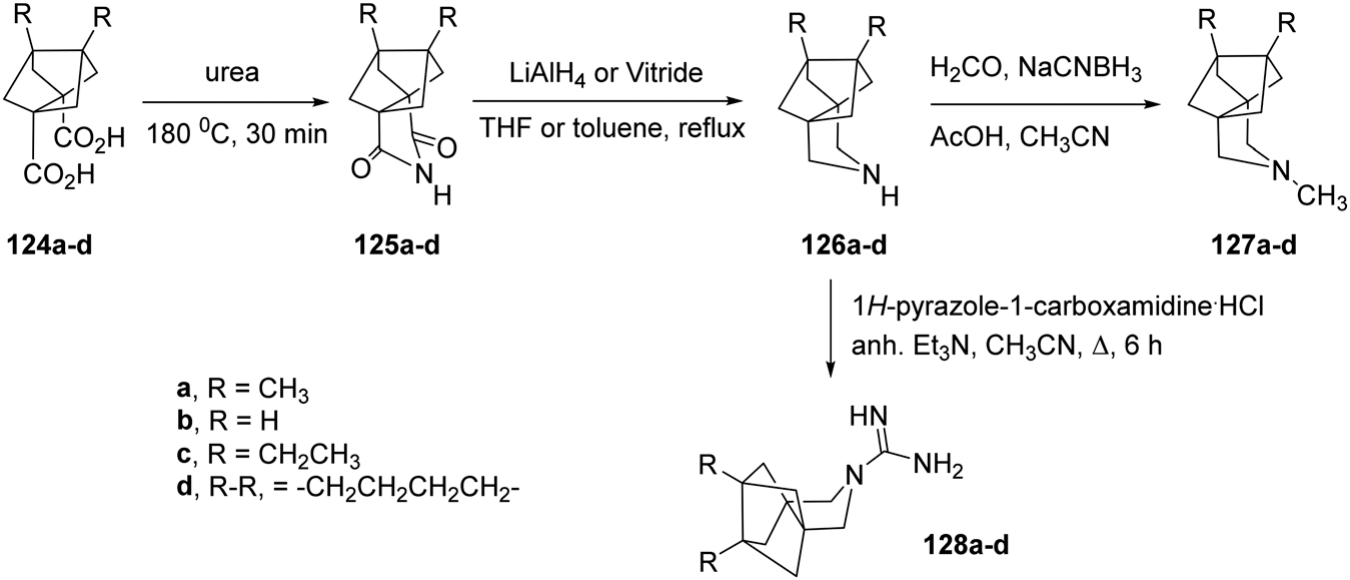
Synthetic procedure for the preparation of the polycyclic pyrrolidine and guanidine derivatives 124a–d–128a–d.^166^

Further synthetic studies aimed to develop polycyclic pyrrolidine scaffolds with potential inhibitory activity against the AM2 channel. The known diacids 124a,b were heated with urea at 180 °C for 30 min to give the corresponding imides 125a,b, which were subsequently reduced to the secondary amines 126a,b in good overall yields.^166^ Subsequent reductive alkylation of 126a,b with formaldehyde and NaCNBH_3_ produced the tertiary amines 127a,b. Because guanidines derived from similar amine precursors had shown AM2 inhibitory properties, compounds 128a,b were synthesized from 126a,b using 1*H*-pyrazole-1-carboxamidine hydrochloride (Scheme 8). Molecular and structural investigations have demonstrated that substituting V27 with a smaller residue, such as alanine (AM2 V27A mutant), disrupts the hydrophobic constriction created by this position and expands the pore near the N-terminal end by roughly 2 Å.^166^ Compounds 125a and 127a exhibited stronger inhibition of the M2-V27A mutant channel compared with their smaller analogues 125b and 127b. Molecular dynamics simulations predicted that bulkier, more hydrophobic ligands could occupy this enlarged pore more efficiently. Thus, two extended analogues of 127a, namely 127c and 127d, were designed and synthesized following the same route.^166^

**Scheme 9:**
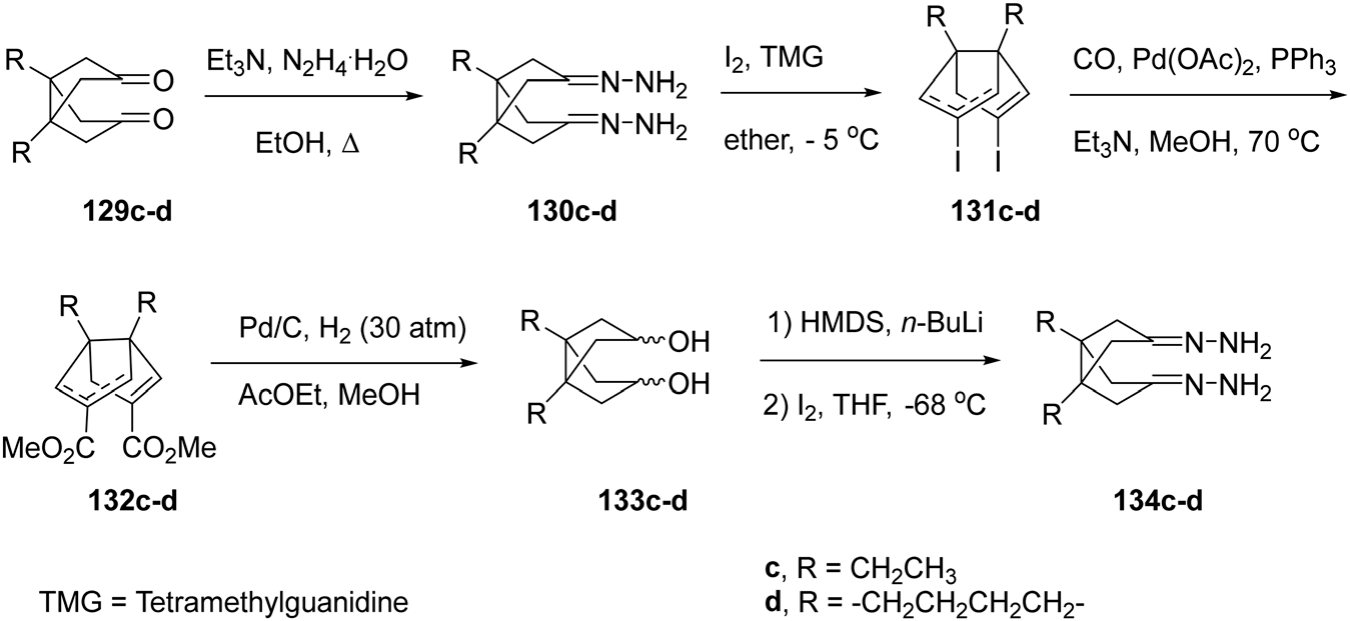
Synthetic procedure for the preparation of diacids 124c,d.^208^

To obtain the extended analogues 127c,d, novel diacids 124c,d were required. These were synthesized from readily available diketones 129c,d through the multi-step sequence shown in Scheme 9.^166^ The approach, previously optimized for bisnoradamantane scaffolds, involved conversion of 129c,d to the corresponding bis-hydrazones 130c,d under reflux in ethanol with triethylamine and hydrazine hydrate, followed by iodination with I_2_/TMG (tetramethylguanidine) to yield the bis-vinyl iodides 131c,d. Subsequent Pd(0)-catalyzed carbonylation (Pd(OAc)_2_/PPh_3_, CO, Et_3_N, MeOH, 70 °C) furnished the diesters 132c,d as mixtures of *syn*/*anti* isomers. Catalytic hydrogenation (Pd/C, H_2_ 30 atm) produced diols 133c,d, which, upon double deprotonation with HMDS/*n*-BuLi and treatment with I_2_ at −68 °C in THF, afforded 134c,d. Finally, hydrolysis of 134c,d provided the target diacids 124c,d in 63% and 80% yield, respectively.^166^

### Design and synthesis of adamantane-based drugs that lead to selective AM2 S31N inhibitors

**Scheme 10:**
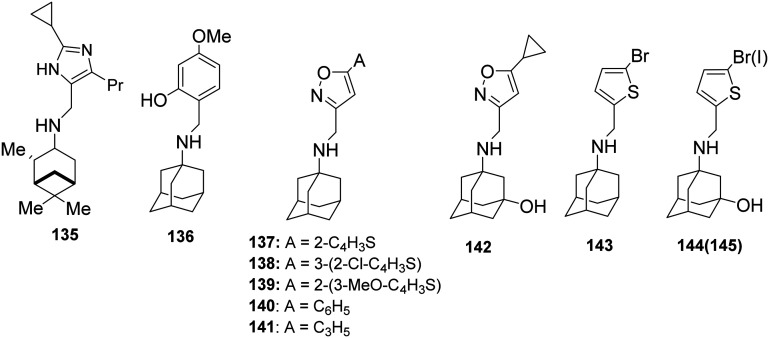
Chemical structure of pinanamine–aryl conjugate 135 (ref. 228) and amantadine–aryl conjugates 136–145,^76,81,140,229^ which block both M2 WT and M2 S31N channels according to EP and inhibit *in vitro* the M2 WT and M2 S31N viruses (in 137 A is 2-thiophenyl, in 138 A is 2-chloro-3-thiophenyl, in 139 A is 3-methoxy-2-thiophenyl, in 140 A is phenyl and in 141 R is cyclopropyl).

After the observation that pinanamine (4)^165^ blocks M2 WT-mediated proton current (Table 2), it was found^128,130^ that linking pinanamine with a heterocycle, *e.g.*, imidazole, through a CH= or CH_2_ linker provided pinanamine–aryl conjugates, *e.g.*, compound 135, which are very potent against influenza A M2 WT and M2 S31N viruses *in vitro* by blocking the corresponding M2 channels (Scheme 10). The %-blocking efficiency at 2 min of 5-methyl-imidazole was less than 30%, suggesting a slow entry blocking compound.^101,105,139^

It was then found that linking amantadine (1) with a heterocycle can produce potent compounds acting against AM2 S31N in both EP and antiviral assays. Using MD simulations and solution NMR in DPC micelles, it was shown that compound 137 (M2J332) in Scheme 10, with a 2-(2-thiophenyl)-isoxazol-3-yl group, blocks the AM2 S31N channel.^76^ The adamantyl group of the drug is bound in the pore between AM2 N31 and G34. In contrast, the aryl group projects towards the N-terminus through the V27 side chains (Fig. 2). This orientation on the AM2 S31N channel is opposite to the orientation of amantadine on the M2 WT channel, *i.e.*, with the amino group facing H37 towards the C-end (Fig. 1).^72,80,221^ Using ssNMR in DMPC lipid bilayers, this position and orientation were also suggested in AM2TM S31N for compound 140 (M2J352).^79^ It was also found that compounds 144, 145^140^ bearing one of the simpler aryl groups used, *i.e.*, correspondingly the 3-bromothiophenyl, 3-iodothiophenyl, block both AM2 WT and AM2 S31N channels. Using NMR in micelles and MD simulations, it was found that the dual inhibitors 144 and 145^140^ are oriented with the aryl head group toward the N-end in the AM2AH S31N pore and toward C-end in the AM2AH WT pore.^81^ For these amantadine–aryl conjugates, between 2013 and 2018, extensive SAR investigations on *in vitro* activity and EP-based blocking potency were conducted,^78,133,135,136,222–226^ and/or binding kinetics^78,136^ were performed by modifying the adamantyl group and the aryl head group.^76,81,139–141^ The kinetics of a ligand binding to its protein target are seen as increasingly important for *in vivo* efficacy in drug discovery^227^ and were critical for amantadine–aryl conjugates.^139,141^ Targeted optimization of binding kinetics was difficult to achieve and required systematic studies, as did those for AM2 channels, to increase understanding of the molecular interactions involved.^139^ It was shown^78,136^ that, similarly to amantadine analogs against AM2 WT^73,147^ or against V27A,^101^ the amantadine–aryl conjugates should have fast *k*_on_, slow *k*_off_ (*e.g.*, compound 141) or slow *k*_on_, slow *k*_off_ (*e.g.*, compound 142) that have low *K*_d_ (= *k*_off_/*k*_on_) values to achieve well *in vitro* antiviral potency.

**Scheme 11:**
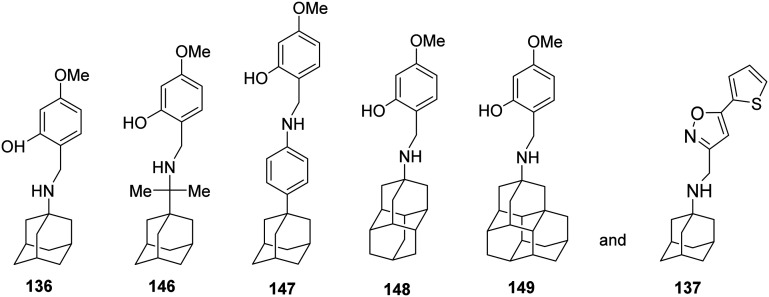
Structures of compounds 136, 146–149, and 137 were used as chemical probes to investigate binding to M2 WT and M2 S31N channels.

Then, in 2020,^106^ the effect of subtle modifications on the structure of an amantadine–aryl conjugate inhibitor on binding kinetics for AM2 WT and AM2 S31N channels was studied using synthetic amantadine variant-CH_2_-aryl derivatives 136, 143, 146–149 (Scheme 11) as sensitive chemical probes for blocking AM2 S31N and AM2 WT channels as well as virus replication in cell culture. Notably, only the binding kinetics for the AM2 S31N channel were very dependent on the length between the adamantane moiety and the first ring of the aryl head group, as observed in 146 and 147, and the girth and length of the adamantane adduct, as observed in 148 and 149. The study of 136, 137, 146–149 with MD simulations in AM2TM S31N and binding free energy calculations (MM-PBSA) showed that all compounds bind in to the AM2 S31N channel with the adamantyl group positioned between V27 and G34 the aryl group projecting out of the channel (toward the N-end) with phenyl (or isoxazole in 137) embedded in the V27 cluster. In this outward binding configuration, an elongation of the ligand by only one methylene in rimantadine 146 or using diamantane as well as triamantane instead of adamantane in 148 and 149, respectively, caused an incomplete entry and facilitated exit, abolishing effective block compared to the amantadine derivatives 136 and 137. In the active M2 S31N blockers 136 and 137, the phenyl and isoxazolyl head groups achieve a deeper binding position, corresponding to measured high *k*_on_/low *k*_off_ and high *k*_on_/high *k*_off_ rate constants, compared to inactive 146–149, which have much lower *k*_on_ and higher *k*_off_. The MD simulations also showed that compounds 136, 146–149 each can block the M2TM WT channel by binding in the longer area from V27–H37, in the inward orientation, with high *k*_on_ and low *k*_off_ rate constants leading to insufficient block. Infection of cell cultures by influenza virus containing AM2 WT or AM2 S31N is inhibited by 136, 146–149, and 137, respectively. While 136 and 137 block infection through the AM2 block mechanism in the AM2 S31N variant, 146–149 may block AM2 S31N virus replication in cell culture through the lysosomotropic effect.^106^

For the most potent conjugates, it was shown that 3-hydroxylation of the 1-adamantyl group, *e.g.*, in compound 142 compared to 141, improved the drug metabolism and pharmacokinetics (DMPK) profile, *e.g.*, the microsomal stability in rat and mouse microsomes against cytochromes, the membrane permeability in Caco-2 cells, and increased recovery. Two potent and promising lead candidates for further development as antiviral drugs are 144 and 145^141^ which have high mouse and human liver microsomal stability (*T*_1/2_ > 145 min) and membrane permeability (>200 nm s^−1^) also inhibit both currently circulating oseltamivir-sensitive and -resistant human influenza A viruses (H1N1 and H3N2) with EC_50_ values ranging from 0.4 to 2.8 μM and a selectivity index of >100.^141^

In a recent study,^230^ based on the structure of the amantadine–aryl conjugates 136, 137 that inhibit AM2 S31N, amantadine was replaced by 16 other adamantanamines. Thus, 36 new compounds were synthesized and tested against AM2 WT and the five amantadine-resistant viruses bearing the AM2 with the mutation L26F, or V27A, or A30T, S31N, or G34E, aiming at identifying inhibitors against multiple M2 mutant-amantadine-resistant viruses. From this study, 16 compounds were identified that inhibited *in vitro* influenza A viruses with AM2 WT or L26F channels. However, compounds 146–149, which are conjugates of a rimantadine analog or the diamantylamine or the 4-(1-adamantyl)benzenamine with the 2-hydroxy-4-methoxyphenyl group, were *in vitro* inhibitors against the three influenza A viruses M2 WT or L26F or S31N, while compound 147 also inhibited *in vitro* the M2 G34E virus, and compound 148 also inhibited *in vitro* the AM2 A30T virus. Using EP, it was shown that compound 147 was an efficient blocker of the AM2 WT and AM2 L26F channels, compound 148 blocked the AM2 WT channel, and compound 149 blocked the AM2 WT, L26F, and V27A channels. For these compounds, a preliminary drug metabolism and pharmacokinetics study was conducted.^230^

In another study^231^ amantadine–aryl conjugates were tested against four amantadine resistant M2 mutants among avian and human influenza A H5N1 strains circulating between 2002 and 2019: the single AM2 S31N and V27A mutants, and the S31N/L26I and S31N/V27A double mutants S31N/L26I and S31N/V27A double mutants. Utilizing TEVC assays, structurally diverse M2 inhibitors were screened against these single and double mutant channels. Compound 139^231^ (Scheme 10) was found to significantly block all three M2 mutant channels and *in vitro* replication: AM2 S31N, AM2 S31N/L26I, and AM2 S31N/V27A.

**Scheme 12:**
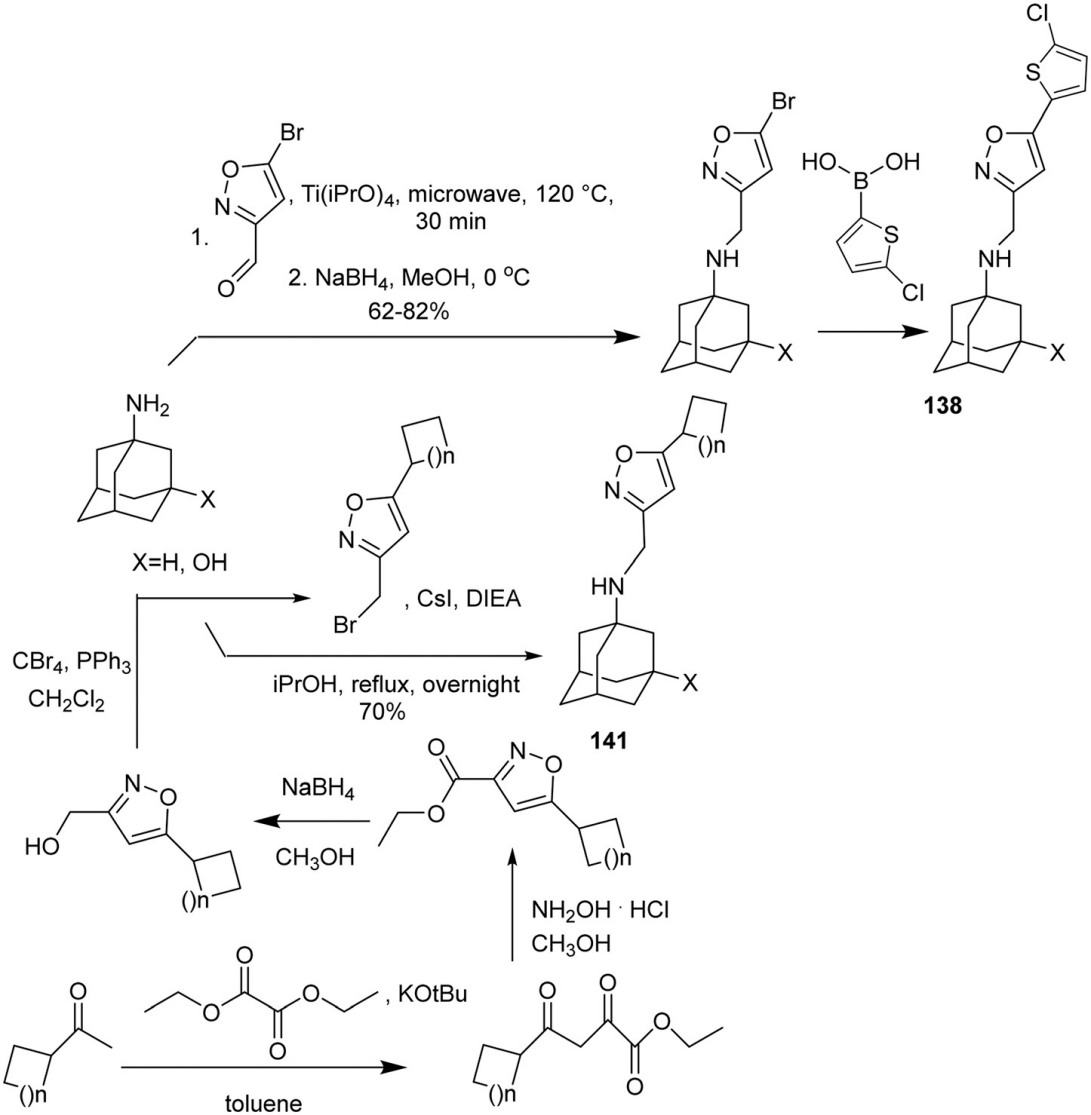
Synthetic procedures leading to amantadine–aryl conjugates, *e.g.*, 137 (M2J332),^76^140 (M2J352),^76^138,^81^141,^235^ as potent blockers of the M2 S31N channel and virus replication.

EP studies combined with MD simulations were applied to rationalize the resistance^149,232^ of AM2 S31N viruses to amantadine–aryl conjugates (*e.g.*, of compound 142) and intriguingly^149,232^ to mutation L46P outside the M2 S31N channel. A few examples of procedures applied for the synthesis of amantadine–aryl conjugates^76,81,139,141,233,234^ are shown in Scheme 12.

**Scheme 13:**
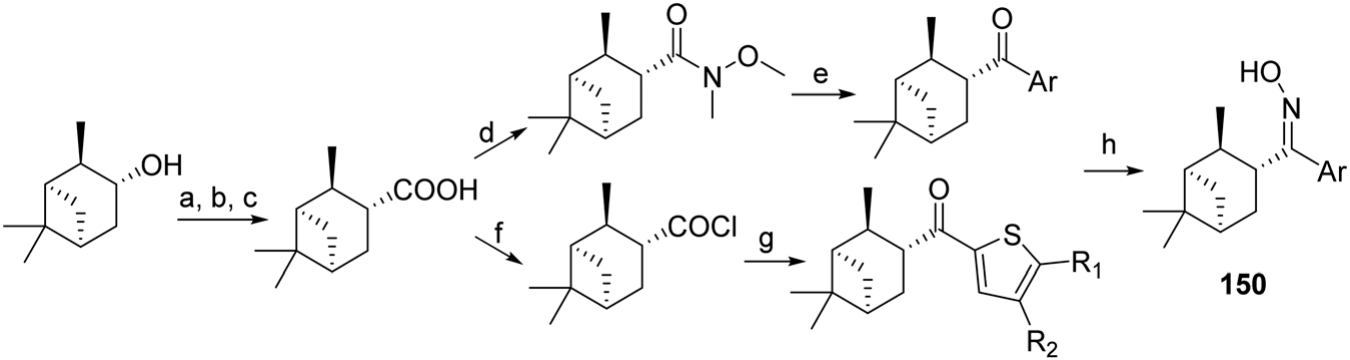
Synthetic procedures lead to pinanyl-thiophenyl oximes, which are potent blockers of AM2 S31N channel and virus replication.^132^ Reagents and conditions: (a) PDC, CH_2_Cl_2_, rt, overnight; (b) TosMIC, *t*-BuOK, DMSO, 60 °C, 48 h; (c) H_2_SO_4_, CH_3_COOH, reflux, overnight; (a) → (c) 36.4%; (d) *N*,*O*-dimethylhydroxylamine hydrochloride, TBTU, triethylamine, CH_3_CN, rt, 1 h; (e) ArLi, ether, 0 °C, 30 min; −78 °C, 3 h; (f) SOCl_2_, cat. DMF, reflux, 2 h; (g) substituted thiophene, SnCl_4_, dry CH_2_Cl_2_, 0 °C, 30 min; (h) pyridine, NH_2_OH·HCl, 80 °C, 24 h.

Interestingly, AM2 S31N blockers, such as pinanyl-thiophenyl oximes 150, have been synthesized as potent blockers of AM2 S31N, having a novel structure^132^ using (−)-isopinocampheol as raw material (Scheme 13).

### Propellanamine analogs of adamantanamines

**Scheme 14:**
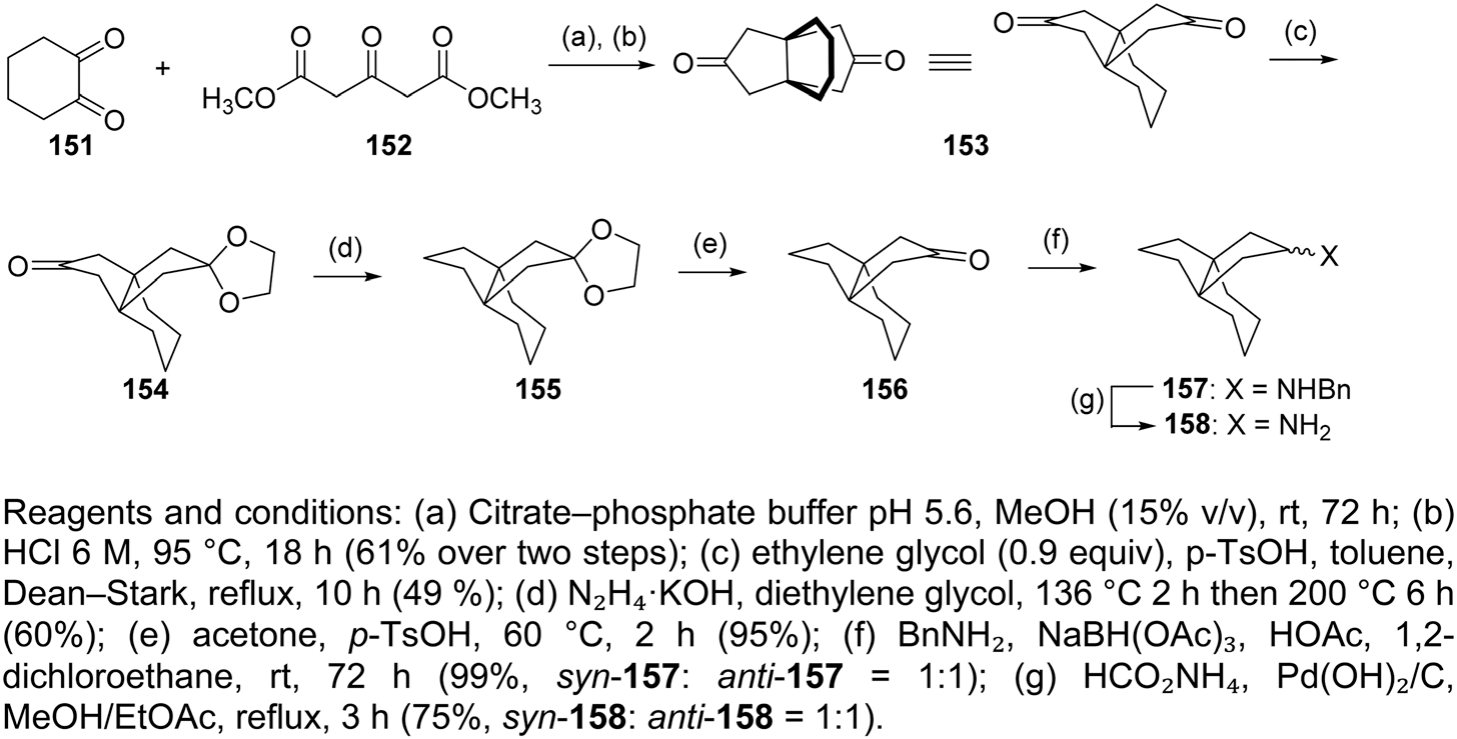
Synthetic route toward diastereomeric [4.3.3]propellan-8-amines.^166^

**Scheme 15:**
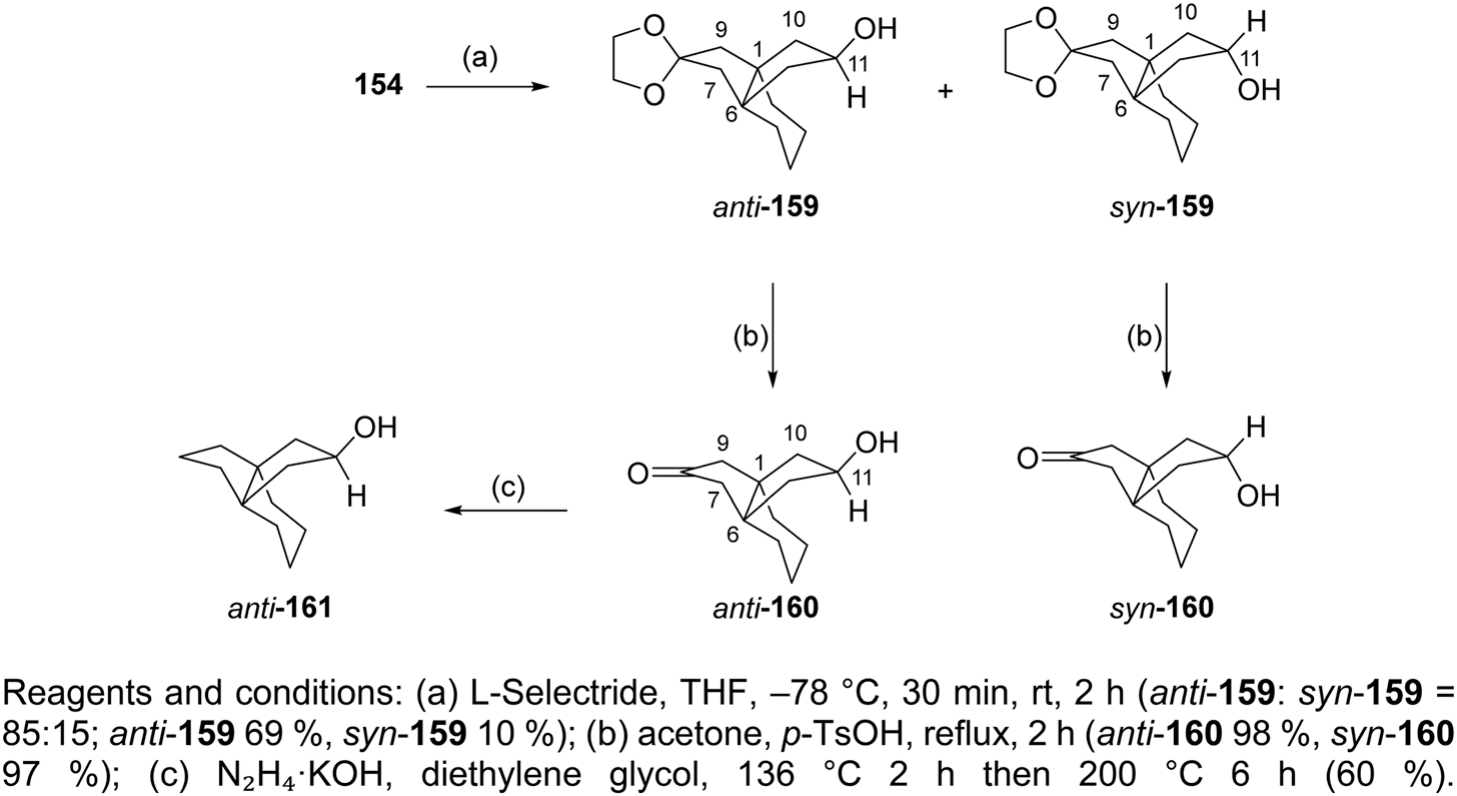
Diastereoselective synthesis of alcohol *anti*-161 from monoketal 154.^208^

The tricyclic [4.3.3]propellan-8-amines were synthesized^208^ as conformationally constrained analogues of amantadine. These rigid polycyclic systems were conceived to explore how subtle variations in cage topology affect channel blocking and NMDA receptor binding. The synthesis began with a modified Weiss–Cook reaction between cyclohexane-1,2-dione (151) and dimethyl 3-oxoglutarate (152) to afford the diketone 153, which was subsequently converted into the key propellanedione intermediate (Scheme 14).^208^ Diastereoselective reduction with L-selectride followed by Wolff–Kishner reduction and acetal manipulation furnished the diastereomerically pure alcohol *anti*-161 (Scheme 15).^208^ Further Mitsunobu inversion and S_N_2 azidation yielded the diastereomeric primary amines *syn*-158 and *anti*-158, which were evaluated for both NMDA receptor affinity and anti-influenza A activity. Interestingly, despite structural resemblance to amantadine and comparable binding to the 1-(1-phenylcyclohexyl)piperidine (PCP) site (*K*_i_ ≈ 11 μM), neither diastereomer inhibited influenza A replication, demonstrating that modifying the cage geometry separates antiviral from NMDA antagonistic activity.

## Conclusions

Combining structural studies with advancements in computational methods, can help design and synthesize adamantane-based blockers that target wild-type or/and mutant AM2 channels. The key structural design principles for saturated polycyclic amine-based blockers against AM2 WT, V27A, and L26F are grounded inadvancements the fundamental mechanism of AM2 proton blocking. These principles involve incorporating features that enable a part of the molecule specifically, the carbon framework connected to an amine group-to function as a proton mimic. This allows these inhibitors to occupy the primary proton-binding site within the channel. Second-generation adamantanamines are known to occlude the AM2 S31 pore and block proton transport. Interestingly many saturated adamantanamines inhibit S31N strains, although the mechanism behind this inhibition has not yet been identified.^199^ Examples of synthetic chemistry that have led to the development of complex adamantanamines, saturated polycyclic amines, and second-generation adamantane-based inhibitors are presented. An extensive and long-term study of the druggable M2 channel has provided the scientific community with essential tools for structure-based drug design, a synthetic chemistry toolkit, and a library of adamantane-based compounds that show promise as effective antivirals, especially given the frequent mutations of the AM2 protein in viruses (Table 5).

**Table 5:** Summary table of representative adamantane-based AM2 inhibitors categorized by structural and mechanistic features (color in the 4th column describes a different class of an inhibitor)

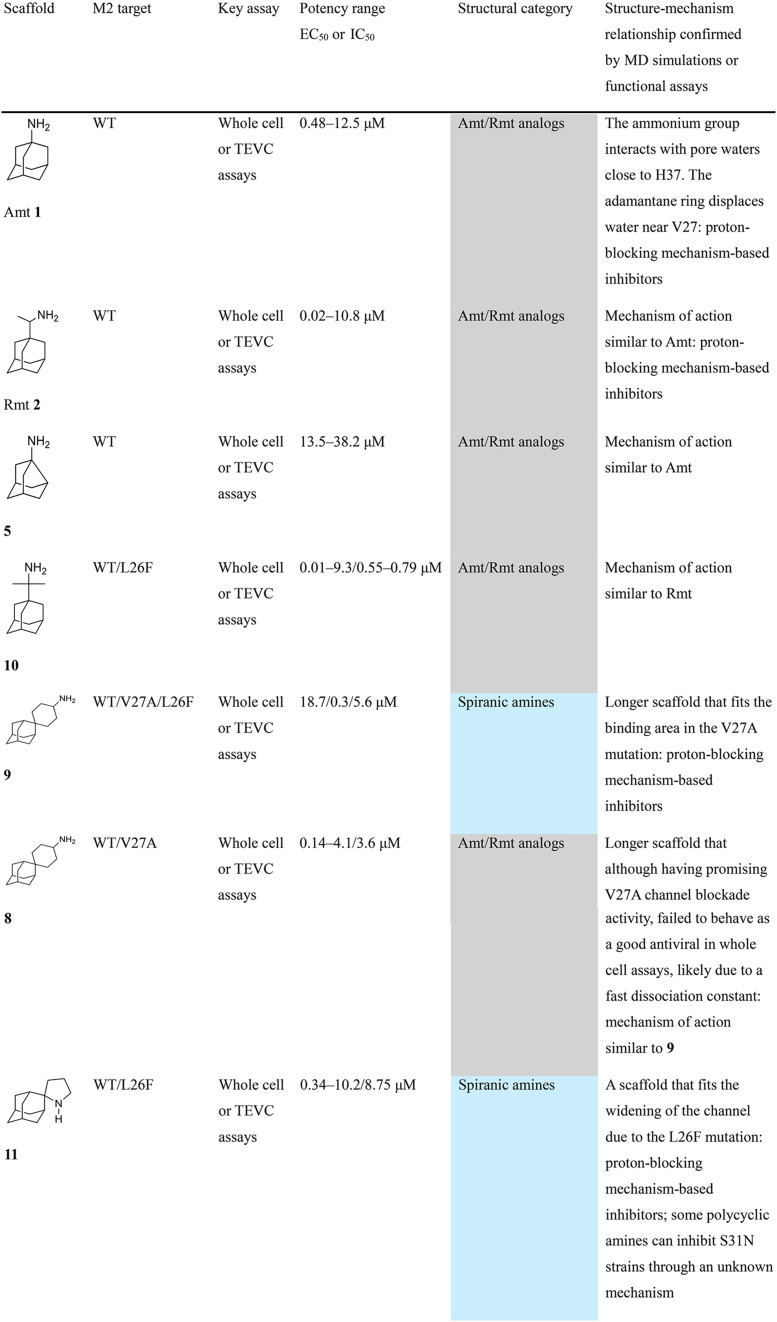 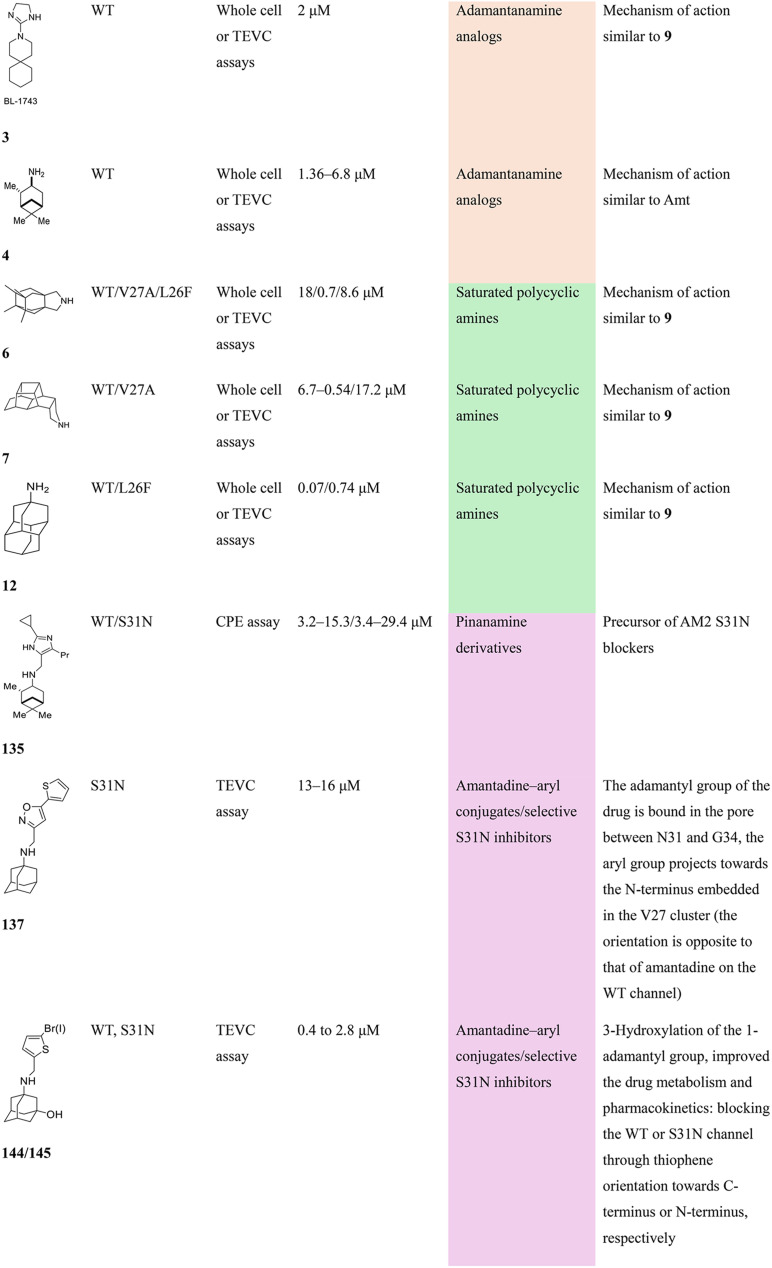

## Conflicts of interest

The authors declare no conflict of interest.

## Data Availability

Data are available upon request.

## References

[cit1] Hong M., DeGrado W. F. (2012). Structural Basis for Proton Conduction and Inhibition by the Influenza M2 Protein. Protein Sci..

[cit2] Zhou H. X., Cross T. A. (2013). Modeling the Membrane Environment Has Implications for Membrane Protein Structure and Function: Influenza A M2 Protein. Protein Sci..

[cit3] Pielak R. M., Chou J. J. (2011). Influenza M2 Proton Channels. Biochim. Biophys. Acta, Biomembr..

[cit4] Wang J., Qiu J. X., Soto C., Degrado W. F. (2011). Structural and Dynamic Mechanisms for the Function and Inhibition of the M2 Proton Channel from Influenza A Virus. Curr. Opin. Struct. Biol..

[cit5] Georgiou K., Kolocouris A. (2025). Conformational Heterogeneity and Structural Features for Function of the Prototype Viroporin Influenza AM2. Biochim. Biophys. Acta, Biomembr..

[cit6] Georgiou K., Kolokouris D., Kolocouris A. (2025). Molecular Biophysics and Inhibition Mechanism of Influenza Virus A M2 Viroporin by Adamantane-Based Drugs – Challenges in Designing Antiviral Agents. J. Struct. Biol.: X.

[cit7] Aledavood E., Selmi B., Estarellas C., Masetti M. (2022). From Acid Activation Mechanisms of Proton Conduction to Design of Inhibitors of the M2 Proton Channel of Influenza A Virus. Front. Mol. Biosci..

[cit8] Rossman J. S., Lamb R. A. (2011). Influenza Virus Assembly and Budding. Virology.

[cit9] Mtambo S. E., Amoako D. G., Somboro A. M., Agoni C., Lawal M. M., Gumede N. S., Khan R. B., Kumalo H. M. (2021). Influenza Viruses: Harnessing the Crucial Role of the M2 Ion-Channel and Neuraminidase toward Inhibitor Design. Molecules.

[cit10] Wang J. (2016). M2 As a Target to Combat Influenza Drug Resistance: What Does the Evidence Say?. Future Virol..

[cit11] Jalily P. H., Duncan M. C., Fedida D., Wang J., Tietjen I. (2020). Put a Cork in It: Plugging the M2 Viral Ion Channel to Sink Influenza. Antiviral Res..

[cit12] Duque M. D. , Torres E., Valverde E., Barniol M., Guardiola S., Rey M. and Vázquez S., Inhibitors of the M2 channel of influenza A virus, Recent advances in pharmaceutical sciences, ed. D. Muñoz-Torrero, Transworld research network, 2011, vol. 661

[cit13] Wang J., Li F., Ma C. (2015). Recent Progress in Designing Inhibitors That Target the Drug-Resistant M2 Proton Channels from the Influenza A Viruses. Biopolymers.

[cit14] Kumar G., Sakharam K. A. (2024). Tackling Influenza A Virus by M2 Ion Channel Blockers: Latest Progress and Limitations. Eur. J. Med. Chem..

[cit15] Jalily P. H., Duncan M. C., Fedida D., Wang J., Tietjen I. (2020). Put a Cork in It: Plugging the M2 Viral Ion Channel to Sink Influenza. Antiviral Res..

[cit16] Shiryaev V. A., Klimochkin Yu. N. (2025). Viroporins: Important Targets for the Development of New Inhibitors of Viral Replication. Russ. Chem. Bull..

[cit17] Shiryaev V. A., Klimochkin Y. N. (2020). Heterocyclic Inhibitors of Viroporins in the Design of Antiviral Compounds. Chem. Heterocycl. Compd..

[cit18] Pardali V., Giannakopoulou E., Konstantinidi A., Kolocouris A., Zoidis G. (2019). 1,2-Annulated Adamantane Heterocyclic Derivatives as Anti-Influenza α Virus Agents. Croat. Chem. Acta.

[cit19] Dane C., Montgomery A. P., Kassiou M. (2025). The Adamantane Scaffold: Beyond a Lipophilic Moiety. Eur. J. Med. Chem..

[cit20] Peng S., Wang Y., Gao Z., Xia B. (2025). Viroporins beyond Pathogenesis: Ion Channel Properties as the Key to Unlocking a Neglected Antiviral Target. Pharmacol. Res..

[cit21] Ma Y., Frutos-Beltrán E., Kang D., Pannecouque C., De Clercq E., Menéndez-Arias L., Liu X., Zhan P. (2021). Medicinal Chemistry Strategies for Discovering Antivirals Effective against Drug-Resistant Viruses. Chem. Soc. Rev..

[cit22] Shin W.-J., Seong B. L. (2019). Novel Antiviral Drug Discovery Strategies to Tackle Drug-Resistant Mutants of Influenza Virus Strains. Expert Opin. Drug Discovery.

[cit23] Martin K., Helenius A. (1991). Nuclear Transport of Influenza Virus Ribonucleoproteins: The Viral Matrix Protein (M1) Promotes Export and Inhibits Import. Cell.

[cit24] Pinto L. H., Holsinger L. J., Lamb R. A. (1992). Influenza Virus M2 Protein Has Ion Channel Activity. Cell.

[cit25] Ciampor F., Bayley P. M., Nermut M. V., Hirst E. M. A., Sugrue R. J., Hay A. J. (1992). Evidence That the Amantadine-Induced, M2-Mediated Conversion of Influenza A Virus Hemagglutinin to the Low PH Conformation Occurs in an Acidic Trans Golgi Compartment. Virology.

[cit26] Grambas S., Hay A. J. (1992). Maturation of Influenza a Virus Hemagglutinin-Estimates of the PH Encountered during Transport and Its Regulation by the M2 Protein. Virology.

[cit27] Sugrue R. J., Bahadur G., Zambon M. C., Hall-Smith M., Douglas A. R., Hay A. J. (1990). Specific Structural Alteration of the Influenza Haemagglutinin by Amantadine. EMBO J..

[cit28] Nayak D. P., Balogun R. A., Yamada H., Zhou Z. H., Barman S. (2009). Influenza Virus Morphogenesis and Budding. Virus Res..

[cit29] Rossman J. S., Lamb R. A. (2011). Influenza Virus Assembly and Budding. Virology.

[cit30] Sugrue R. J., Hay A. J. (1991). Structural Characteristics of the M2 Protein of Influenza a Viruses: Evidence That It Forms a Tetrameric Channel. Virology.

[cit31] Pinto L. H., Lamb R. A. (2007). Controlling Influenza Virus Replication by Inhibiting Its Proton Channel. Mol. BioSyst..

[cit32] Chizhmakov I. V., Geraghty F. M., Ogden D. C., Hayhurst A., Antoniou M., Hay A. J. (1996). Selective Proton Permeability and PH Regulation of the Influenza Virus M2 Channel Expressed in Mouse Erythroleukaemia Cells. J. Physiol..

[cit33] Wang C., Lamb R. A., Pinto L. H. (1994). Direct Measurement of the Influenza A Virus M2 Protein Ion Channel Activity in Mammalian Cells. Virology.

[cit34] Lamb R. A., Zebedee S. L., Richardson C. D. (1985). Influenza Virus M2 Protein Is an Integral Membrane Protein Expressed on the Infected-Cell Surface. Cell.

[cit35] Rossman J. S., Jing X., Leser G. P., Balannik V., Pinto L. H., Lamb R. A. (2010). Influenza Virus M2 Ion Channel Protein Is Necessary for Filamentous Virion Formation. J. Virol..

[cit36] Park E. K., Castrucci M. R., Portner A., Kawaoka Y. (1998). The M2 Ectodomain Is Important for Its Incorporation into Influenza A Virions. J. Virol..

[cit37] Pinto L. H., Lamb R. A. (2007). Controlling Influenza Virus Replication by Inhibiting Its Proton Channel. Mol. BioSyst..

[cit38] Chen B. J., Leser G. P., Jackson D., Lamb R. A. (2008). The Influenza Virus M2 Protein Cytoplasmic Tail Interacts with the M1 Protein and Influences Virus Assembly at the Site of Virus Budding. J. Virol..

[cit39] Ma C., Polishchuk A. L., Ohigashi Y., Stouffer A. L., Schon A., Magavern E., Jing X., Lear J. D., Freire E., Lamb R. A., DeGrado W. F., Pinto L. H. (2009). Identification of the Functional Core of the Influenza A Virus A/M2 Proton-Selective Ion Channel. Proc. Natl. Acad. Sci. U. S. A..

[cit40] Hay A. J., Wolstenholme A. J., Skehel J. J., Smith M. H. (1985). The Molecular Basis of the Specific Anti-Influenza Action of Amantadine. EMBO J..

[cit41] Cady S. D., Schmidt-Rohr K., Wang J., Soto C. S., DeGrado W. F., Hong M. (2010). Structure of the Amantadine Binding Site of Influenza M2 Proton Channels in Lipid Bilayers. Nature.

[cit42] Duff K. C., Gilchrist P. J., Saxena A. M., Bradshaw J. P. (1994). Neutron Diffraction Reveals the Site of Amantadine Blockade in the Influenza A M2 Ion Channel. Virology.

[cit43] Salom D., Hill B. R., Lear J. D., DeGrado W. F. (2000). PH-Dependent Tetramerization and Amantadine Binding of the Transmembrane Helix of M2 from the Influenza A Virus. Biochemistry.

[cit44] Stouffer A. L., Acharya R., Salom D., Levine A. S., Di Costanzo L., Soto C. S., Tereshko V., Nanda V., Stayrook S., DeGrado W. F. (2008). Structural Basis for the Function and Inhibition of an Influenza Virus Proton Channel. Nature.

[cit45] Duff K. C., Ashley R. H. (1992). The Transmembrane Domain of Influenza A M2 Protein Forms Amantadine-Sensitive Proton Channels in Planar Lipid Bilayers. Virology.

[cit46] Wang C., Takeuchi K., Pinto L. H., Lamb R. A. (1993). Ion Channel Activity of Influenza A Virus M2 Protein: Characterization of the Amantadine Block. J. Virol..

[cit47] Liang R., Swanson J. M. J., Madsen J. J., Hong M., De Grado W. F., Voth G. A. (2016). Acid Activation Mechanism of the Influenza A M2 Proton Channel. Proc. Natl. Acad. Sci. U. S. A..

[cit48] Hu J., Fu R., Nishimura K., Zhang L., Zhou H. X., Busath D. D., Vijayvergiya V., Cross T. A. (2006). Histidines, Heart of the Hydrogen Ion Channel from Influenza A Virus: Toward an Understanding of Conductance and Proton Selectivity. Proc. Natl. Acad. Sci. U. S. A..

[cit49] Wang C., Lamb R. A., Pinto L. H. (1995). Activation of the M2 Ion Channel of Influenza Virus: A Role for the Transmembrane Domain Histidine Residue. Biophys. J..

[cit50] Hu J., Fu R., Nishimura K., Zhang L., Zhou H. X., Busath D. D., Vijayvergiya V., Cross T. A. (2006). Histidines, Heart of the Hydrogen Ion Channel from Influenza A Virus: Toward an Understanding of Conductance and Proton Selectivity. Proc. Natl. Acad. Sci. U. S. A..

[cit51] Tang Y., Zaitseva F., Lamb R. A., Pinto L. H. (2002). The Gate of the Influenza Virus M2 Proton Channel Is Formed by a Single Tryptophan Residue. J. Biol. Chem..

[cit52] Yi M., Cross T. A., Zhou H. X. (2008). A Secondary Gate as a Mechanism for Inhibition of the M2 Proton Channel by Amantadine. J. Phys. Chem. B.

[cit53] Nishimura K., Kim S., Zhang L., Cross T. A. (2002). The Closed State of a H+ Channel Helical Bundle Combining Precise Orientational and Distance Restraints from Solid State NMR. Biochemistry.

[cit54] Thomaston J. L., Alfonso-Prieto M., Woldeyes R. A., Fraser J. S., Klein M. L., Fiorin G., DeGrado W. F. (2015). High-Resolution Structures of the M2 Channel from Influenza A Virus Reveal Dynamic Pathways for Proton Stabilization and Transduction. Proc. Natl. Acad. Sci. U. S. A..

[cit55] Thomaston J. L., Woldeyes R. A., Nakane T., Yamashita A., Tanaka T., Koiwai K., Brewster A. S., Barad B. A., Chen Y., Lemmin T., Uervirojnangkoorn M., Arima T., Kobayashi J., Masuda T., Suzuki M., Sugahara M., Sauter N. K., Tanaka R., Nureki O., Tono K., Joti Y., Nango E., Iwata S., Yumoto F., Fraser J. S., DeGrado W. F. (2017). XFEL Structures of the Influenza M2 Proton Channel: Room Temperature Water Networks and Insights into Proton Conduction. Proc. Natl. Acad. Sci. U. S. A..

[cit56] Holsinger L. J., Nichani D., Pinto L. H., Lamb R. A. (1994). Influenza A Virus M2 Ion Channel Protein: A Structure-Function Analysis. J. Virol..

[cit57] McCown M. F., Pekosz A. (2006). Distinct Domains of the Influenza a Virus M2 Protein Cytoplasmic Tail Mediate Binding to the M1 Protein and Facilitate Infectious Virus Production. J. Virol..

[cit58] Rossman J. S., Jing X., Leser G. P., Lamb R. A. (2010). Influenza Virus M2 Protein Mediates ESCRT-Independent Membrane Scission. Cell.

[cit59] Sharma M., Yi M., Dong H., Qin H., Peterson E., Busath D. D., Zhou H.-X., Cross T. A. (2010). Insight into the Mechanism of the Influenza A Proton Channel from a Structure in a Lipid Bilayer. Science.

[cit60] Hu J., Asbury T., Achuthan S., Li C., Bertram R., Quine J. R., Fu R., Cross T. A. (2007). Backbone Structure of the Amantadine-Blocked Trans-Membrane Domain M2 Proton Channel from Influenza A Virus. Biophys. J..

[cit61] Stouffer A. L., Acharya R., Salom D., Levine A. S., Di Costanzo L., Soto C. S., Tereshko V., Nanda V., Stayrook S., DeGrado W. F. (2008). Structural Basis for the Function and Inhibition of an Influenza Virus Proton Channel. Nature.

[cit62] Nishimura K., Kim S., Zhang L., Cross T. A. (2002). The Closed State of a H+ Channel Helical Bundle Combining Precise Orientational and Distance Restraints from Solid State NMR. Biochemistry.

[cit63] Acharya R., Carnevale V., Fiorin G., Levine B. G., Polishchuk A. L., Balannik V., Samish I., Lamb R. A., Pinto L. H., DeGrado W. F., Klein M. L. (2010). Structure and Mechanism of Proton Transport through the Transmembrane Tetrameric M2 Protein Bundle of the Influenza A Virus. Proc. Natl. Acad. Sci. U. S. A..

[cit64] Acharya R., Carnevale V., Fiorin G., Levine B. G., Polishchuk A. L., Balannik V., Samish I., Lamb R. A., Pinto L. H., DeGrado W. F., Klein M. L. (2010). Structure and Mechanism of Proton Transport through the Transmembrane Tetrameric M2 Protein Bundle of the Influenza A Virus. Proc. Natl. Acad. Sci. U. S. A..

[cit65] Thomaston J. L., Alfonso-Prieto M., Woldeyes R. A., Fraser J. S., Klein M. L., Fiorin G., DeGrado W. F. (2015). High-Resolution Structures of the M2 Channel from Influenza A Virus Reveal Dynamic Pathways for Proton Stabilization and Transduction. Proc. Natl. Acad. Sci. U. S. A..

[cit66] Schnell J. R., Chou J. J. (2008). Structure and Mechanism of the M2 Proton Channel of Influenza A Virus. Nature.

[cit67] Cady S. D., Schmidt-Rohr K., Wang J., Soto C. S., DeGrado W. F., Hong M. (2010). Structure of the Amantadine Binding Site of Influenza M2 Proton Channels in Lipid Bilayers. Nature.

[cit68] Cady S., Wang T., Hong M. (2011). Membrane-Dependent Effects of a Cytoplasmic Helix on the Structure and Drug Binding of the Influenza Virus M2 Protein. J. Am. Chem. Soc..

[cit69] Pielak R. M., Schnell J. R., Chou J. J. (2009). Mechanism of Drug Inhibition and Drug Resistance of Influenza A M2 Channel. Proc. Natl. Acad. Sci. U. S. A..

[cit70] Andreas L. B., Eddy M. T., Pielak R. M., Chou J., Griffin R. G. (2010). Magic Angle Spinning NMR Investigation of Influenza A M218−60: Support for an Allosteric Mechanism of Inhibition. J. Am. Chem. Soc..

[cit71] Pielak R. M., Oxenoid K., Chou J. J. (2011). Structural Investigation of Rimantadine Inhibition of the AM2-BM2 Chimera Channel of Influenza Viruses. Structure.

[cit72] Thomaston J. L., Polizzi N. F., Konstantinidi A., Wang J., Kolocouris A., Degrado W. F. (2018). Inhibitors of the M2 Proton Channel Engage and Disrupt Transmembrane Networks of Hydrogen-Bonded Waters. J. Am. Chem. Soc..

[cit73] Thomaston J. L., Samways M. L., Konstantinidi A., Ma C., Hu Y., Bruce Macdonald H. E., Wang J., Essex J. W., Degrado W. F., Kolocouris A. (2021). Rimantadine Binds to and Inhibits the Influenza A M2 Proton Channel without Enantiomeric Specificity. Biochemistry.

[cit74] Astrahan P., Arkin I. T. (2011). Resistance Characteristics of Influenza to Amino-Adamantyls. Biochim. Biophys. Acta, Biomembr..

[cit75] Leonov H., Astrahan P., Krugliak M., Arkin I. T. (2011). How Do Aminoadamantanes Block the Influenza M2 Channel, and How Does Resistance Develop?. J. Am. Chem. Soc..

[cit76] Wang J. J., Wu Y., Ma C., Fiorin G., Pinto L. H., Lamb R. A., Klein M. L., Degrado W. F. (2013). Structure and Inhibition of the Drug-Resistant S31N Mutant of the M2 Ion Channel of Influenza A Virus. Proc. Natl. Acad. Sci. U. S. A..

[cit77] Shrestha S. S., Swerdlow D. L., Borse R. H., Prabhu V. S., Finelli L., Atkins C. Y., Owusu-Edusei K., Bell B., Mead P. S., Biggerstaff M., Brammer L., Davidson H., Jernigan D., Jhung M. A., Kamimoto L. A., Merlin T. L., Nowell M., Redd S. C., Reed C., Schuchat A., Meltzer M. I. (2011). Estimating the Burden of 2009 Pandemic Influenza A (H1N1) in the United States (April 2009-April 2010). Clin. Infect. Dis..

[cit78] Wang Y., Hu Y., Xu S., Zhang Y., Musharrafieh R., Hau R. K., Ma C., Wang J. (2018). In Vitro Pharmacokinetic Optimizations of AM2-S31N Channel Blockers Led to the Discovery of Slow-Binding Inhibitors with Potent Antiviral Activity against Drug-Resistant Influenza A Viruses. J. Med. Chem..

[cit79] Williams J. K., Tietze D., Wang J., Wu Y., Degrado W. F., Hong M. (2013). Drug-Induced Conformational and Dynamical Changes of the S31N Mutant of the Influenza M2 Proton Channel Investigated by Solid-State NMR. J. Am. Chem. Soc..

[cit80] Cady S. D., Wang J., Wu Y., Degrado W. F., Hong M. (2011). Specific Binding of Adamantane Drugs and Direction of Their Polar Amines in the Pore of the Influenza M2 Transmembrane Domain in Lipid Bilayers and Dodecylphosphocholine Micelles Determined by NMR Spectroscopy. J. Am. Chem. Soc..

[cit81] Wu Y., Canturk B., Jo H., Ma C., Gianti E., Klein M. L., Pinto L. H., Lamb R. A., Fiorin G., Wang J., Degrado W. F. (2014). Flipping in the Pore: Discovery of Dual Inhibitors That Bind in Different Orientations to the Wild-Type versus the Amantadine-Resistant S31n Mutant of the Influenza a Virus M2 Proton Channel. J. Am. Chem. Soc..

[cit82] Thomaston J. L., Konstantinidi A., Liu L., Lambrinidis G., Tan J., Caffrey M., Wang J., Degrado W. F., Kolocouris A. (2020). X-Ray Crystal Structures of the Influenza M2 Proton Channel Drug-Resistant V27A Mutant Bound to a Spiro-Adamantyl Amine Inhibitor Reveal the Mechanism of Adamantane Resistance. Biochemistry.

[cit83] Cady S., Wang T., Hong M. (2011). Membrane-Dependent Effects of a Cytoplasmic Helix on the Structure and Drug Binding of the Influenza Virus M2 Protein. J. Am. Chem. Soc..

[cit84] Davies W. L., Grunert R. R., Haff R. F., Mcgahen J. W., Neumayer E. M., Paulshock M., Watts J. C., Wood T. R., Hermann E. C., Hoffmann C. E. (1964). Antiviral Activity of 1-Adamantanamine (Amantadine). Science.

[cit85] Wendel H. A., Snyder M. T., Pell S. (1966). Trial of Amantadine in Epidemic Influenza. Clin. Pharmacol. Ther..

[cit86] Schwab R. S. (1969). Amantadine in the Treatment of Parkinson's Disease. JAMA, J. Am. Med. Assoc..

[cit87] Blanpied T. A., Clarke R. J., Johnson J. W. (2005). Amantadine Inhibits NMDA Receptors by Accelerating Channel Closure during Channel Block. J. Neurosci..

[cit88] Stouffer A. L., Ma C., Cristian L., Ohigashi Y., Lamb R. A., Lear J. D., Pinto L. H., DeGrado W. F. (2008). The Interplay of Functional Tuning, Drug Resistance, and Thermodynamic Stability in the Evolution of the M2 Proton Channel from the Influenza A Virus. Structure.

[cit89] Furuse Y., Suzuki A., Kamigaki T., Oshitani H. (2009). Evolution of the M Gene of the Influenza A Virus in Different Host Species: Large-Scale Sequence Analysis. Virol. J..

[cit90] Bright R. A., Medina M. J., Xu X., Perez-Oronoz G., Wallis T. R., Davis X. M., Povinelli L., Cox N. J., Klimov A. I. (2005). Incidence of Adamantane Resistance among Influenza A (H3N2) Viruses Isolated Worldwide from 1994 to 2005: A Cause for Concern. Lancet.

[cit91] Lan Y., Zhang Y., Dong L., Wang D., Huang W., Xin L., Yang L., Zhao X., Li Z., Wang W., Li X., Xu C., Yang L., Guo J., Wang M., Peng Y., Gao Y., Guo Y., Wen L., Jiang T., Shu Y. (2010). A Comprehensive Surveillance of Adamantane Resistance among Human Influenza A Virus Isolated from Mainland China between 1956 and 2009. Antiviral Ther..

[cit92] Abed Y., Goyette N., Boivin G. (2005). Generation and Characterization of Recombinant Influenza A (H1N1) Viruses Harboring Amantadine Resistance Mutations. Antimicrob. Agents Chemother..

[cit93] Brown A. N., McSharry J. J., Weng Q., Driebe E. M., Engelthaler D. M., Sheff K., Keim P. S., Nguyen J., Drusano G. L. (2010). In Vitro System for Modeling Influenza A Virus Resistance under Drug Pressure. Antimicrob. Agents Chemother..

[cit94] Li D., Saito R., Suzuki Y., Sato I., Zaraket H., Dapat C., Caperig-Dapat I. M., Suzuki H. (2009). In Vivo and in Vitro Alterations in Influenza A/H3N2 Virus M2 and Hemagglutinin Genes: Effect of Passage in MDCK-SIAT1 Cells and Conventional MDCK Cells. J. Clin. Microbiol..

[cit95] Dong G., Peng C., Luo J., Wang C., Han L., Wu B., Ji G., He H. (2015). Adamantane-Resistant Influenza a Viruses in the World (1902-2013): Frequency and Distribution of M2 Gene Mutations. PLoS One.

[cit96] Garcia V., Aris-Brosou S. (2014). Comparative Dynamics and Distribution of Influenza Drug Resistance Acquisition to Protein M2 and Neuraminidase Inhibitors. Mol. Biol. Evol..

[cit97] Zhang L., Shao Y., Zou X., Cui Y., Ding S., Chen Y., Wu Q., Mu S., Li X., Yang Q., Cao B., Deng T. (2025). A Primary Oseltamivir-Resistant Mutation in Influenza Hemagglutinin and Its Implications for Antiviral Resistance Surveillance. Nat. Commun..

[cit98] Musharrafieh R., Ma C., Wang J. (2018). Profiling the in Vitro Drug-Resistance Mechanism of Influenza A Viruses towards the AM2-S31N Proton Channel Blockers. Antiviral Res..

[cit99] Musharrafieh R., Lagarias P. I., Ma C., Tan G. S., Kolocouris A., Wang J. (2019). The L46P Mutant
Confers a Novel Allosteric Mechanism of Resistance Toward the Influenza A Virus M2 S31N Proton Channel Blockers. Mol. Pharmacol..

[cit100] Musharrafieh R., Lagarias P., Ma C., Hau R., Romano A., Lambrinidis G., Kolocouris A., Wang J. (2020). Investigation of the Drug Resistance Mechanism of M2-S31N Channel Blockers through Biomolecular Simulations and Viral Passage Experiments. ACS Pharmacol. Transl. Sci..

[cit101] Barniol-Xicota M., Gazzarrini S., Torres E., Hu Y., Wang J., Naesens L., Moroni A., Vázquez S. (2017). Slow but Steady Wins the Race: Dissimilarities among New Dual Inhibitors of the Wild-Type and the V27A Mutant M2 Channels of Influenza A Virus. J. Med. Chem..

[cit102] Kolocouris N., Kolocouris A., Foscolos G. B., Fytas G., Neyts J., Padalko E., Balzarini J., Snoeck R., Andrei G., De Clercq E. (1996). Synthesis and Antiviral Activity Evaluation of Some New Aminoadamantane Derivatives. 2. J. Med. Chem..

[cit103] Scott C., Kankanala J., Foster T. L., Goldhill D. H., Bao P., Simmons K., Pingen M., Bentham M., Atkins E., Loundras E., Elderfield R., Claridge J. K., Thompson J., Stilwell P. R., Tathineni R., McKimmie C. S., Targett-Adams P., Schnell J. R., Cook G. P., Evans S., Barclay W. S., Foster R., Griffin S. (2020). Site-Directed M2 Proton Channel Inhibitors Enable Synergistic Combination Therapy for Rimantadine-Resistant Pandemic Influenza. PLoS Pathog..

[cit104] Aldrich P. E., Hermann E. C., Meier W. E., Paulshock M., Prichard W. W., Synder J. A., Watts J. C. (1971). Antiviral Agents. 2. Structure-Activity Relations of Compounds Related to 1-Adamantanamine. J. Med. Chem..

[cit105] Drakopoulos A., Tzitzoglaki C., McGuire K., Hoffmann A., Ma C., Freudenberger K., Konstantinidi A., Kolocouris D., Hutterer J., Gauglitz G., Wang J., Schmidtke M., Busath D. D., Kolocouris A., Kolokouris D., Ma C., Freudenberger K., Hutterer J., Gauglitz G., Wang J., Schmidtke M., Busath D. D., Kolocouris A. (2018). Unraveling the Binding, Proton Blockage, and Inhibition of Influenza M2 WT and S31N by Rimantadine Variants. ACS Med. Chem. Lett..

[cit106] Tzitzoglaki C., McGuire K., Lagarias P., Konstantinidi A., Hoffmann A., Fokina N. A., Ma C. C., Papanastasiou I. P., Schreiner P. R., Vázquez S., Schmidtke M., Wang J., Busath D. D., Kolocouris A., Vazquez S., Schmidtke M., Wang J., Busath D. D., Kolocouris A. (2020). Chemical Probes for Blocking of Influenza A M2 Wild-Type and S31N Channels. ACS Chem. Biol..

[cit107] Astrahan P., Flitman-Tene R., Bennett E. R., Krugliak M., Gilon C., Arkin I. T. (2011). Quantitative Analysis of Influenza M2 Channel Blockers. Biochim. Biophys. Acta, Biomembr..

[cit108] Santner P., Martins J. M. D. S., Laursen J. S., Behrendt L., Riber L., Olsen C. A., Arkin I. T., Winther J. R., Willemoës M., Lindorff-Larsen K. (2018). A Robust Proton Flux (PHlux) Assay for Studying the Function and Inhibition of the Influenza A M2 Proton Channel. Biochemistry.

[cit109] Santner P., Martins J. M. D. S., Kampmeyer C., Hartmann-Petersen R., Laursen J. S., Stein A., Olsen C. A., Arkin I. T., Winther J. R., Willemoës M., Lindorff-Larsen K. (2018). Random Mutagenesis Analysis of the Influenza A M2 Proton Channel Reveals Novel Resistance Mutants. Biochemistry.

[cit110] Salom D., Hill B. R., Lear J. D., DeGrado W. F. (2000). PH-Dependent Tetramerization and Amantadine Binding of the Transmembrane Helix of M2 from the Influenza A Virus. Biochemistry.

[cit111] Balannik V., Wang J., Ohigashi Y., Jing X., Magavern E., Lamb R. A., DeGrado W. F., Pinto L. H. (2009). Design and Pharmacological Characterization of Inhibitors of Amantadine-Resistant Mutants of the M2 Ion Channel of Influenza A Virus. Biochemistry.

[cit112] Czabotar P. E., Martin S. R., Hay A. J. (2004). Studies of Structural Changes in the M2 Proton Channel of Influenza a Virus by Tryptophan Fluorescence. Virus Res..

[cit113] Stylianakis I., Kolocouris A., Kolocouris N., Fytas G., Foscolos G. B., Padalko E., Neyts J., De Clercq E. (2003). Spiro[Pyrrolidine-2,2′-Adamantanes]: Synthesis, Anti-Influenza Virus Activity and Conformational Properties. Bioorg. Med. Chem. Lett..

[cit114] Cristian L., Lear J. D., DeGrado W. F. (2003). Use of Thiol-Disulfide Equilibria to Measure the Energetics of Assembly of Transmembrane Helices in Phospholipid Bilayers. Proc. Natl. Acad. Sci. U. S. A..

[cit115] Rosenberg M. R., Casarotto M. G. (2010). Coexistence of Two Adamantane Binding Sites in the Influenza A M2 Ion Channel. Proc. Natl. Acad. Sci. U. S. A..

[cit116] Pielak R. M., Schnell J. R., Chou J. J. (2009). Mechanism of Drug Inhibition and Drug Resistance of Influenza A M2 Channel. Proc. Natl. Acad. Sci. U. S. A..

[cit117] Tu Q., Pinto L. H., Luo G., Shaughnessy M. A., Mullaney D., Kurtz S., Krystal M., Lamb R. A. (1996). Characterization of Inhibition of M2 Ion Channel Activity by BL-1743, an Inhibitor of Influenza A Virus. J. Virol..

[cit118] Pinto L. H., Dieckmann G. R., Gandhi C. S., Papworth C. G., Braman J., Shaughnessy M. A., Lear J. D., Lamb R. A., DeGrado W. F. (1997). A Functionally Defined Model for the M2 Proton Channel of Influenza A Virus Suggests a Mechanism for Its Ion Selectivity. Proc. Natl. Acad. Sci. U. S. A..

[cit119] Wang C., Lamb R. A., Pinto L. H. (1994). Direct Measurement of the Influenza A Virus M2 Protein Ion Channel Activity in Mammalian Cells. Virology.

[cit120] Shimbo K., Brassard D. L., Lamb R. A., Pinto L. H. (1996). Ion Selectivity and Activation of the M2 Ion Channel of Influenza Virus. Biophys. J..

[cit121] Duque M. D., Ma C., Torres E., Wang J., Naesens L., Juárez-Jiménez J., Camps P., Luque F. J., DeGrado W. F., Lamb R. A., Pinto L. H., Vázquez S. (2011). Exploring the Size Limit of Templates for Inhibitors of the M2 Ion Channel of Influenza A Virus. J. Med. Chem..

[cit122] Torres E., Fernández R., Miquet S., Font-Bardia M., Vanderlinden E., Naesens L., Vázquez S. (2012). Synthesis and Anti-Influenza a Virus Activity of 2,2-Dialkylamantadines and Related Compounds. ACS Med. Chem. Lett..

[cit123] Torres E., Leiva R., Gazzarrini S., Rey-Carrizo M., Frigolé-Vivas M., Moroni A., Naesens L., Vázquez S. (2014). Azapropellanes with Anti-Influenza a Virus Activity. ACS Med. Chem. Lett..

[cit124] Rey-Carrizo M., Gazzarrini S., Llabrés S., Frigolé-Vivas M., Juárez-Jiménez J., Font-Bardia M., Naesens L., Moroni A., Luque F. J., Vázquez S. (2015). New Polycyclic Dual Inhibitors of the Wild Type and the V27A Mutant M2 Channel of the Influenza A Virus with Unexpected Binding Mode. Eur. J. Med. Chem..

[cit125] Rey-carrizo M., Torres E., Ma C., Barniol-xicota M., Wang J., Wu Y., Naesens L., Degrado W. F., Lamb R. A., Pinto L. H., Vázquez S., Va S. (2013). 3-Azatetracyclo[5.2.1.15,8.01,5]Undecane Derivatives: From Wild-Type Inhibitors of the M2 Ion Channel of Influenza A Virus to Derivatives with Potent Activity against the V27A Mutant. J. Med. Chem..

[cit126] McGuire K. L., Wallentine S., Gordon N. A., Mohl G., Jensen M. D., Harrison R., Busath D. D. (2016). Divalent Copper Complexes as Influenza a M2 S31N Blockers. Biophys. J..

[cit127] Gordon N. A., McGuire K. L., Wallentine S. K., Mohl G. A., Lynch J. D., Harrison R. G., Busath D. D. (2017). Divalent Copper Complexes as Influenza A M2 Inhibitors. Antiviral Res..

[cit128] Zhao X., Jie Y., Rosenberg M. R., Wan J., Zeng S., Cui W., Xiao Y., Li Z., Tu Z., Casarotto M. G., Hu W. (2012). Design and Synthesis of Pinanamine Derivatives as Anti-Influenza A M2 Ion Channel Inhibitors. Antiviral Res..

[cit129] Zhao X., Zhang Z. W., Cui W., Chen S., Zhou Y., Dong J., Jie Y., Wan J., Xu Y., Hu W. (2015). Identification of Camphor Derivatives as Novel M2 Ion Channel Inhibitors of Influenza A Virus. MedChemComm.

[cit130] Dong J., Chen S., Li R., Cui W., Jiang H., Ling Y., Yang Z., Hu W. (2016). Imidazole-Based Pinanamine Derivatives: Discovery of Dual Inhibitors of the Wild-Type and Drug-Resistant Mutant of the Influenza A Virus. Eur. J. Med. Chem..

[cit131] Zhao X., Li R., Zhou Y., Xiao M., Ma C., Yang Z., Zeng S., Du Q., Yang C., Jiang H., Hu Y., Wang K., Mok C. K. P., Sun P., Dong J., Cui W., Wang J., Tu Y., Yang Z., Hu W. (2018). Discovery of Highly Potent Pinanamine-Based Inhibitors against Amantadine- and Oseltamivir-Resistant Influenza A Viruses. J. Med. Chem..

[cit132] Dong J., Xiao M., Ma Q., Zhang G., Zhao W., Kong M., Zhang Y., Qiu L., Hu W. (2020). Design and Synthesis of Pinane Oxime Derivatives as Novel Anti-Influenza Agents. Bioorg. Chem..

[cit133] Tzitzoglaki C., McGuire K., Lagarias P., Konstantinidi A., Hoffmann A., Fokina N. A., Ma C. C., Papanastasiou I. P., Schreiner P. R., Vázquez S., Schmidtke M., Wang J., Busath D. D., Kolocouris A., Vazquez S., Schmidtke M., Wang J., Busath D. D., Kolocouris A. (2020). Chemical Probes for Blocking of Influenza A M2 Wild-Type and S31N Channels. ACS Chem. Biol..

[cit134] Drakopoulos A., Tzitzoglaki C., McGuire K., Hoffmann A., Ma C., Freudenberger K., Konstantinidi A., Kolocouris D., Hutterer J., Gauglitz G., Wang J., Schmidtke M., Busath D. D., Kolocouris A., Kolokouris D., Ma C., Freudenberger K., Hutterer J., Gauglitz G., Wang J., Schmidtke M., Busath D. D., Kolocouris A. (2018). Unraveling the Binding, Proton Blockage, and Inhibition of Influenza M2 WT and S31N by Rimantadine Variants. ACS Med. Chem. Lett..

[cit135] Wang J., Ma C., Wang J., Jo H., Canturk B., Fiorin G., Pinto L. H., Lamb R. A., Klein M. L., DeGrado W. F. (2013). Discovery of Novel Dual Inhibitors of the Wild-Type and the Most Prevalent Drug-Resistant Mutant, S31N, of the M2 Proton Channel from Influenza A Virus. J. Med. Chem..

[cit136] Hu Y., Hau R. K., Wang Y., Tuohy P., Zhang Y., Xu S., Ma C., Wang J. (2018). Structure-Property Relationship Studies of Influenza A Virus AM2-S31N Proton Channel Blockers. ACS Med. Chem. Lett..

[cit137] Drakopoulos A., Tzitzoglaki C., Ma C. C., Freudenberger K., Hoffmann A., Hu Y., Gauglitz G. G., Schmidtke M., Wang J., Kolocouris A. (2017). Affinity of Rimantadine Enantiomers against Influenza A/M2 Protein Revisited. ACS Med. Chem. Lett..

[cit138] Hu W., Zeng S., Li C., Jie Y., Li Z., Chen L. (2010). Identification of Hits as Matrix-2 Protein Inhibitors through the Focused Screening of a Small Primary Amine Library. J. Med. Chem..

[cit139] Wang Y., Hu Y., Xu S., Zhang Y., Musharrafieh R., Hau R. K., Ma C., Wang J. (2018). In Vitro Pharmacokinetic Optimizations of AM2-S31N Channel Blockers Led to the Discovery of Slow-Binding Inhibitors with Potent Antiviral Activity against Drug-Resistant Influenza A Viruses. J. Med. Chem..

[cit140] Wang J., Ma C., Wang J., Jo H., Canturk B., Fiorin G., Pinto L. H., Lamb R. A., Klein M. L., DeGrado W. F. (2013). Discovery of Novel
Dual Inhibitors of the Wild-Type and the Most Prevalent Drug-Resistant Mutant, S31N, of the M2 Proton Channel from Influenza A Virus. J. Med. Chem..

[cit141] Hu Y., Hau R. K., Wang Y., Tuohy P., Zhang Y., Xu S., Ma C., Wang J. (2018). Structure-Property Relationship Studies of Influenza A Virus AM2-S31N Proton Channel Blockers. ACS Med. Chem. Lett..

[cit142] Stampolaki M., Hoffmann A., Tekwani K., Georgiou K., Tzitzoglaki C., Ma C., Becker S., Schmerer P., Döring K., Stylianakis I., Turcu A. L., Wang J., Vázquez S., Andreas L. B., Schmidtke M., Kolocouris A. (2023). A Study of the Activity of Adamantyl Amines against Mutant Influenza A M2 Channels Identified a Polycyclic Cage Amine Triple Blocker, Explored by Molecular Dynamics Simulations and Solid-State NMR**. ChemMedChem.

[cit143] Wang J., Cady S. D., Balannik V., Pinto L. H., DeGrado W. F., Hong M. (2009). Discovery of Spiro-Piperidine Inhibitors and Their Modulation of the Dynamics of the M2 Proton Channel from Influenza A Virus. J. Am. Chem. Soc..

[cit144] Movellan K. T., Wegstroth M., Overkamp K., Leonov A., Becker S., Andreas L. B. (2020). Imidazole-Imidazole Hydrogen Bonding in the PH-Sensing Histidine Side Chains of Influenza A M2. J. Am. Chem. Soc..

[cit145] Kolocouris A., Tzitzoglaki C., Johnson F. B., Zell R., Wright A. K., Cross T. A., Tietjen I., Fedida D., Busath D. D. (2014). Aminoadamantanes with Persistent in Vitro Efficacy against H1N1 (2009) Influenza A. J. Med. Chem..

[cit146] Tzitzoglaki C., Wright A., Freudenberger K., Hoffmann A., Tietjen I., Stylianakis I., Kolarov F., Fedida D., Schmidtke M., Gauglitz G., Cross T. A., Kolocouris A. (2017). Binding and Proton Blockage by Amantadine Variants of the Influenza M2WT and M2S31N Explained. J. Med. Chem..

[cit147] Drakopoulos A., Tzitzoglaki C., Ma C. C., Freudenberger K., Hoffmann A., Hu Y., Gauglitz G. G., Schmidtke M., Wang J., Kolocouris A. (2017). Affinity of Rimantadine Enantiomers against Influenza A/M2 Protein Revisited. ACS Med. Chem. Lett..

[cit148] Wright A. K., Batsomboon P., Dai J., Hung I., Zhou H. X., Dudley G. B., Cross T. A. (2016). Differential Binding of Rimantadine Enantiomers to Influenza A M2 Proton Channel. J. Am. Chem. Soc..

[cit149] Musharrafieh R., Lagarias P., Ma C., Hau R., Romano A., Lambrinidis G., Kolocouris A., Wang J. (2020). Investigation of the Drug Resistance Mechanism of M2-S31N Channel Blockers through Biomolecular Simulations and Viral Passage Experiments. ACS Pharmacol. Transl. Sci..

[cit150] Tekwani Movellan K., Wegstroth M., Overkamp K., Leonov A., Becker S., Andreas L. B. (2023). Real-Time Tracking of Drug Binding to Influenza A M2 Reveals a High Energy Barrier. J. Struct. Biol.: X.

[cit151] Ma C., Polishchuk A. L., Ohigashi Y., Stouffer A. L., Schön A., Magavern E., Jing X., Lear J. D., Freire E., Lamb R. A., DeGrado W. F., Pinto L. H. (2009). Identification of the Functional Core of the Influenza A Virus A/M2 Proton-Selective Ion Channel. Proc. Natl. Acad. Sci. U. S. A..

[cit152] Kellokumpu S. (2019). Golgi PH, Ion and Redox Homeostasis: How Much Do They Really Matter?. Front. Cell Dev. Biol..

[cit153] Rey-Carrizo M., Barniol-Xicota M., Ma C., Frigolé-Vivas M., Torres E., Naesens L., Llabrés S., Juárez-Jiménez J., Luque F. J., Degrado W. F., Lamb R. A., Pinto L. H., Vázquez S. (2014). Easily Accessible Polycyclic Amines That Inhibit the Wild-Type and Amantadine-Resistant Mutants of the M2 Channel of Influenza A Virus. J. Med. Chem..

[cit154] Huang J., Rauscher S., Nawrocki G., Ran T., Feig M., De Groot B. L., Grubmüller H., MacKerell A. D. (2016). CHARMM36m: An Improved Force Field for Folded and Intrinsically Disordered Proteins. Nat. Methods.

[cit155] Thomaston J. L., Polizzi N. F., Konstantinidi A., Wang J., Kolocouris A., Degrado W. F. (2018). Inhibitors of the M2 Proton Channel Engage and Disrupt Transmembrane Networks of Hydrogen-Bonded Waters. J. Am. Chem. Soc..

[cit156] Kurtz S., Luo G., Hahnenberger K. M., Brooks C., Gecha O., Ingalls K., Numata K. I., Krystal M. (1995). Growth Impairment Resulting from Expression of Influenza Virus M2 Protein in Saccharomyces Cerevisiae: Identification of a Novel Inhibitor of Influenza Virus. Antimicrob. Agents Chemother..

[cit157] Zoidis G., Fytas C., Papanastasiou I., Foscolos G. B., Fytas G., Padalko E., De Clercq E., Naesens L., Neyts J., Kolocouris N. (2006). Heterocyclic Rimantadine Analogues with Antiviral Activity. Bioorg. Med. Chem..

[cit158] Rey-Carrizo M., Barniol-Xicota M., Ma C., Frigolé-Vivas M., Torres E., Naesens L., Llabrés S., Juárez-Jiménez J., Luque F. J., Degrado W. F., Lamb R. A., Pinto L. H., Vázquez S. (2014). Easily Accessible Polycyclic Amines That Inhibit the Wild-Type and Amantadine-Resistant Mutants of the M2 Channel of Influenza A Virus. J. Med. Chem..

[cit159] Wang J., Ma C., Fiorin G., Carnevale V., Wang T., Hu F., Lamb R. A., Pinto L. H., Hong M., Klein M. L., Degrado W. F. (2011). Molecular Dynamics Simulation Directed Rational Design of Inhibitors Targeting Drug-Resistant Mutants of Influenza A Virus M2. J. Am. Chem. Soc..

[cit160] Camps P., Duque M. D., Vázquez S., Naesens L., De Clercq E., Sureda F. X., López-Querol M., Camins A., Pallàs M., Prathalingam S. R., Kelly J. M., Romero V., Ivorra D., Cortés D. (2008). Synthesis and Pharmacological Evaluation of Several Ring-Contracted Amantadine Analogs. Bioorg. Med. Chem..

[cit161] Duque M. D., Camps P., Profire L., Montaner S., Vázquez S., Sureda F. X., Mallol J., López-Querol M., Naesens L., De Clercq E., Radhika Prathalingam S., Kelly J. M. (2009). Synthesis and Pharmacological Evaluation of (2-Oxaadamant-1-Yl)Amines. Bioorg. Med. Chem..

[cit162] Barniol-Xicota M., Gazzarrini S., Torres E., Hu Y., Wang J., Naesens L., Moroni A., Vázquez S. (2017). Slow but Steady Wins the Race: Dissimilarities among New Dual Inhibitors of the Wild-Type and the V27A Mutant M2 Channels of Influenza A Virus. J. Med. Chem..

[cit163] Torres E., Fernández R., Miquet S., Font-Bardia M., Vanderlinden E., Naesens L., Vázquez S. (2012). Synthesis and Anti-Influenza a Virus Activity of 2,2-Dialkylamantadines and Related Compounds. ACS Med. Chem. Lett..

[cit164] Valverde E., Sureda F. X., Vázquez S. (2014). Novel Benzopolycyclic Amines with NMDA Receptor Antagonist Activity. Bioorg. Med. Chem..

[cit165] Hu W., Zeng S., Li C., Jie Y., Li Z., Chen L. (2010). Identification of Hits as Matrix-2 Protein Inhibitors through the Focused Screening of a Small Primary Amine Library. J. Med. Chem..

[cit166] Rey-Carrizo M., Gazzarrini S., Llabrés S., Frigolé-Vivas M., Juárez-Jiménez J., Font-Bardia M., Naesens L., Moroni A., Luque F. J., Vázquez S. (2015). New Polycyclic Dual Inhibitors of the Wild Type and the V27A Mutant M2 Channel of the Influenza A Virus with Unexpected Binding Mode. Eur. J. Med. Chem..

[cit167] Tzitzoglaki C., Wright A., Freudenberger K., Hoffmann A., Tietjen I., Stylianakis I., Kolarov F., Fedida D., Schmidtke M., Gauglitz G., Cross T. A., Kolocouris A. (2017). Binding and Proton Blockage by Amantadine Variants of the Influenza M2WT and M2S31N Explained. J. Med. Chem..

[cit168] Spyrakis F., Ahmed M. H., Bayden A. S., Cozzini P., Mozzarelli A., Kellogg G. E. (2017). The Roles of Water in the Protein Matrix: A Largely Untapped Resource for Drug Discovery. J. Med. Chem..

[cit169] Kollman P. A., Massova I., Reyes C., Kuhn B., Huo S., Chong L., Lee M., Lee T., Duan Y., Wang W., Donini O., Cieplak P., Srinivasan J., Case D. A., Cheatham T. E. (2000). Calculating Structures and Free Energies of Complex Molecules: Combining Molecular Mechanics and Continuum Models. Acc. Chem. Res..

[cit170] Wang E., Sun H., Wang J., Wang Z., Liu H., Zhang J. Z. H., Hou T. (2019). End-Point Binding Free Energy Calculation with MM/PBSA and MM/GBSA: Strategies and Applications in Drug Design. Chem. Rev..

[cit171] Bennett C. H. (1976). Efficient Estimation of Free Energy Differences from Monte Carlo Data. J. Comput. Phys..

[cit172] Shirts M. R., Chodera J. D. (2008). Statistically Optimal Analysis of Samples from Multiple Equilibrium States. J. Chem. Phys..

[cit173] Homeyer N., Ioannidis H., Kolarov F., Gauglitz G. G., Zikos C., Kolocouris A., Gohlke H. (2016). Interpreting Thermodynamic Profiles of Aminoadamantane Compounds Inhibiting the M2 Proton Channel of Influenza A by Free Energy Calculations. J. Chem. Inf. Model..

[cit174] Gkeka P., Eleftheratos A.-S., Kolocouris A., Cournia Z., Eleftheratos S., Kolocouris A., Cournia Z. (2013). Free Energy Calculations Reveal the Origin of Binding Preference for Aminoadamantane Blockers of Influenza A/M2TM Pore. J. Chem. Theory Comput..

[cit175] Ioannidis H., Drakopoulos A., Tzitzoglaki C., Homeyer N., Kolarov F., Gkeka P., Freudenberger K., Liolios C., Gauglitz G. G., Cournia Z., Gohlke H., Kolocouris A. (2016). Alchemical Free Energy Calculations and Isothermal Titration Calorimetry Measurements of Aminoadamantanes Bound to the Closed State of Influenza A/M2TM. J. Chem. Inf. Model..

[cit176] Tian C., Kasavajhala K., Belfon K. A. A., Raguette L., Huang H., Migues A. N., Bickel J., Wang Y., Pincay J., Wu Q., Simmerling C. (2020). Ff19SB: Amino-Acid-Specific Protein Backbone Parameters Trained against Quantum Mechanics Energy Surfaces in Solution. J. Chem. Theory Comput..

[cit177] Shirts M. R., Chodera J. D. (2008). Statistically Optimal Analysis of Samples from Multiple Equilibrium States. J. Chem. Phys..

[cit178] Wanka L., Iqbal K., Schreiner P. R. (2013). The Lipophilic Bullet Hits the Targets: Medicinal Chemistry of Adamantane Derivatives. Chem. Rev..

[cit179] Lamoureux G., Artavia G. (2010). Use of the Adamantane Structure in Medicinal Chemistry. Curr. Med. Chem..

[cit180] Štimac A., Šekutor M., Mlinarić-Majerski K., Frkanec L., Frkanec R. (2017). Adamantane in Drug Delivery Systems and Surface Recognition. Molecules.

[cit181] Liu J., Obando D., Liao V., Lifa T., Codd R. (2011). The Many Faces of the Adamantyl Group in Drug Design. Eur. J. Med. Chem..

[cit182] Davies W. L., Grunert R. R., Haff R. F., Mcgahen J. W., Neumayer E. M., Paulshock M., Watts J. C., Wood T. R., Hermann E. C., Hoffmann C. E. (1964). Antiviral Activity of 1-Adamantanamine (Amantadine). Science.

[cit183] Whitney J. G., Gregory W. A., Kauer J. C., Roland J. R., Snyder J. A., Benson R. E., Hermann E. C. (1970). Antiviral Agents. I. Bicyclo[2.2.2]Octan- and -Oct-2-Enamines. J. Med. Chem..

[cit184] Aldrich P. E., Hermann E. C., Meier W. E., Paulshock M., Prichard W. W., Snyder J. A., Watts J. C. (1971). Antiviral Agents. 2. Structure-Activity Relationships of Compounds Related to 1-Adamantanamine. J. Med. Chem..

[cit185] Lundahl K., Schut J., Schlatmann J. L. M. A., Paerels G. B., Peters A., Philips-Duphar N. V. (1972). Synthesis and Antiviral Activities of Adamantane Spiro Compounds. 1. Adamantane and Analogous Spiro-3′-Pyrrolidines. J. Med. Chem..

[cit186] Van Hes R., Smit A., Kralt T., Peters A. (1972). Synthesis and Antiviral Activities of Adamantane Spiro Compounds. 21. J. Med. Chem..

[cit187] Kolocouris A., Dimas K., Pannecouque C., Witvrouw M., Foscolos G. B., Stamatiou G., Fytas G., Zoidis G., Kolocouris N., Andrei G., Snoeck R., De Clercq E. (2002). New 2-(1-Adamantylcarbonyl)Pyridine and 1-Acetyladamantane Thiosemicarbazones-Thiocarbonohydrazones: Cell Growth Inhibitory, Antiviral and Antimicrobial Activity Evaluation. Bioorg. Med. Chem. Lett..

[cit188] Kolocouris N., Kolocouris A., Foscolos G. B., Fytas G., Neyts J., Padalko E., Balzarini J., Snoeck R., Andrei G., De Clercq E. (1996). Synthesis and Antiviral Activity Evaluation of Some New Aminoadamantane Derivatives. 2. J. Med. Chem..

[cit189] Zoidis G., Fytas C., Papanastasiou I., Foscolos G. B., Fytas G., Padalko E., De Clercq E., Naesens L., Neyts J., Kolocouris N. (2006). Heterocyclic Rimantadine Analogues with Antiviral Activity. Bioorg. Med. Chem..

[cit190] Tataridis D., Fytas G., Kolocouris A., Fytas C., Kolocouris N., Foscolos G. B., Padalko E., Neyts J., De Clercq E. (2007). Influence of an Additional 2-Amino Substituent of the 1-Aminoethyl Pharmacophore Group on the Potency of Rimantadine against Influenza Virus A. Bioorg. Med. Chem. Lett..

[cit191] Setaki D., Tataridis D., Stamatiou G., Kolocouris A., Foscolos G. B., Fytas G., Kolocouris N., Padalko E., Neyts J., Clercq E. D. (2006). Synthesis, Conformational Characteristics and Anti-Influenza Virus A Activity of Some 2-Adamantylsubstituted Azacycles. Bioorg. Chem..

[cit192] Stamatiou G., Foscolos G. B., Fytas G., Kolocouris A., Kolocouris N., Pannecouque C., Witvrouw M., Padalko E., Neyts J., Clercq E. D. (2003). Heterocyclic Rimantadine Analogues with Antiviral Activity. Bioorg. Med. Chem..

[cit193] Zoidis G., Kolocouris N., Foscolos G. B., Kolocouris A., Fytas G., Karayannis P., Padalko E., Neyts J., De Clercq E. (2003). Are the 2-Isomers of the Drug Rimantadine Active Anti-Influenza A Agents?. Antiviral Chem. Chemother..

[cit194] Kolocouris N., Foscolos G. B., Kolocouris A., Marakos P., Pouli N., Fytas G., Ikeda S., De Clercq E. (1994). Synthesis and Antiviral Activity Evaluation of Some Aminoadamantane Derivatives. J. Med. Chem..

[cit195] Kolocouris N., Zoidis G., Foscolos G. B., Fytas G., Prathalingham S. R., Kelly J. M., Naesens L., De Clercq E. (2007). Design and Synthesis of Bioactive Adamantane Spiro Heterocycles. Bioorg. Med. Chem. Lett..

[cit196] Kolocouris A., Tataridis D., Fytas G., Mavromoustakos T., Foscolos G. B., Kolocouris N., De Clercq E. (1999). Synthesis of 2-(2-Adamantyl)Piperidines and Structure Anti-Influenza Virus A Activity Relationship Study Using a Combination of NMR Spectroscopy and Molecular Modeling. Bioorg. Med. Chem. Lett..

[cit197] Stamatiou G., Kolocouris A., Kolocouris N., Fytas G., Foscolos G. B., Neyts J., De Clercq E. (2001). Novel 3-(2-Adamantyl)Pyrrolidines with Potent Activity against Influenza A Virus - Identification
of Aminoadamantane Derivatives Bearing Two Pharmacophoric Amine Groups. Bioorg. Med. Chem. Lett..

[cit198] Fytas C., Kolocouris A., Fytas G., Zoidis G., Valmas C., Basler C. F. (2010). Influence of an Additional Amino Group on the Potency of Aminoadamantanes against Influenza Virus A. II - Synthesis of Spiropiperazines and in Vitro Activity against Influenza A H3N2 Virus. Bioorg. Chem..

[cit199] Tietjen I., Kwan D. C., Petrich A., Zell R., Antoniadou I. T., Gavriilidou A., Tzitzoglaki C., Rallis M., Fedida D., Sureda F. X., Mestdagh C., Naesens L., Chiantia S., Johnson F. B., Kolocouris A. (2025). Antiviral Mechanisms and Preclinical Evaluation of Amantadine Analogs That Continue to Inhibit Influenza A Viruses with M2S31N-Based Drug Resistance. Antiviral Res..

[cit200] Wang J., Cady S. D., Balannik V., Pinto L. H., DeGrado W. F., Hong M. (2009). Discovery of Spiro-Piperidine Inhibitors and Their Modulation of the Dynamics of the M2 Proton Channel from Influenza A Virus. J. Am. Chem. Soc..

[cit201] Duque M. D., Ma C., Torres E., Wang J., Naesens L., Juárez-Jiménez J., Camps P., Luque F. J., DeGrado W. F., Lamb R. A., Pinto L. H., Vázquez S. (2011). Exploring the Size Limit of Templates for Inhibitors of the M2 Ion Channel of Influenza A Virus. J. Med. Chem..

[cit202] Torres E., Leiva R., Gazzarrini S., Rey-Carrizo M., Frigolé-Vivas M., Moroni A., Naesens L., Vázquez S. (2014). Azapropellanes with Anti-Influenza a Virus Activity. ACS Med. Chem. Lett..

[cit203] Rey-Carrizo M., Gazzarrini S., Llabrés S., Frigolé-Vivas M., Juárez-Jiménez J., Font-Bardia M., Naesens L., Moroni A., Luque F. J., Vázquez S. (2015). New Polycyclic Dual Inhibitors of the Wild Type and the V27A Mutant M2 Channel of the Influenza A Virus with Unexpected Binding Mode. Eur. J. Med. Chem..

[cit204] Rey-carrizo M., Torres E., Ma C., Barniol-xicota M., Wang J., Wu Y., Naesens L., Degrado W. F., Lamb R. A., Pinto L. H., Vázquez S., Va S. (2013). 3-Azatetracyclo[5.2.1.15,8.01,5]Undecane Derivatives: From Wild-Type Inhibitors of the M2 Ion Channel of Influenza A Virus to Derivatives with Potent Activity against the V27A Mutant. J. Med. Chem..

[cit205] Zhao X., Jie Y., Rosenberg M. R., Wan J., Zeng S., Cui W., Xiao Y., Li Z., Tu Z., Casarotto M. G., Hu W. (2012). Design and Synthesis of Pinanamine Derivatives as Anti-Influenza A M2 Ion Channel Inhibitors. Antiviral Res..

[cit206] Zhao X., Zhang Z. W., Cui W., Chen S., Zhou Y., Dong J., Jie Y., Wan J., Xu Y., Hu W. (2015). Identification of Camphor Derivatives as Novel M2 Ion Channel Inhibitors of Influenza A Virus. MedChemComm.

[cit207] Wang J., Ma C., Balannik V., Pinto L. H., Lamb R. A., Degrado W. F. (2011). Exploring the Requirements for the Hydrophobic Scaffold and Polar Amine in Inhibitors of M2 from Influenza A Virus. ACS Med. Chem. Lett..

[cit208] Torres-Gómez H., Lehmkuhl K., Frehland B., Daniliuc C., Schepmann D., Ehrhardt C., Wünsch B. (2015). Stereoselective Synthesis and Pharmacological Evaluation of [4.3.3]Propellan-8-Amines as Analogs of Adamantanamines. Bioorg. Med. Chem..

[cit209] Weigel W. K., Dang H. T., Feceu A., Martin D. B. C. (2022). Direct Radical Functionalization Methods to Access Substituted Adamantanes and Diamondoids. Org. Biomol. Chem..

[cit210] Hrdina R. (2019). Directed C–H Functionalization of the Adamantane Framework. Synthesis.

[cit211] Camps P., Duque M. D., Vázquez S., Naesens L., De Clercq E., Sureda F. X., López-Querol M., Camins A., Pallàs M., Prathalingam S. R., Kelly J. M., Romero V., Ivorra D., Cortés D. (2008). Synthesis and Pharmacological Evaluation of Several Ring-Contracted Amantadine Analogs. Bioorg. Med. Chem..

[cit212] Zoidis G., Kolocouris N., Foscolos G. B., Kolocouris A., Fytas G., Karayannis P., Padalko E., Neyts J., De Clercq E. (2003). Are the 2-Isomers of the Drug Rimantadine Active Anti-Influenza A Agents?. Antiviral Chem. Chemother..

[cit213] Fytas G., Kolocouris N., Foscolos G., Vamvakidès A. (1991). Synthesis and Pharmacological Study of Some Adamantylcyclopentanamines. Eur. J. Med. Chem..

[cit214] Tzitzoglaki C., Drakopoulos A., Konstantinidi A., Stylianakis I., Stampolaki M., Kolocouris A. (2019). Approaches to Primary Tert-Alkyl Amines as Building Blocks. Tetrahedron.

[cit215] Stamatiou G., Foscolos G. B., Fytas G., Kolocouris A., Kolocouris N., Pannecouque C., Witvrouw M., Padalko E., Neyts J., Clercq E. D. (2003). Heterocyclic Rimantadine Analogues with Antiviral Activity. Bioorg. Med. Chem..

[cit216] Kolocouris A., Tataridis D., Fytas G., Mavromoustakos T., Foscolos G. B., Kolocouris N., De Clercq E. (1999). Synthesis of 2-(2-Adamantyl)Piperidines and Structure Anti-Influenza Virus A Activity Relationship Study Using a Combination of NMR Spectroscopy and Molecular Modeling. Bioorg. Med. Chem. Lett..

[cit217] Kolocouris N., Foscolos G. B., Kolocouris A., Marakos P., Pouli N., Fytas G., Ikeda S., De Clercq E. (1994). Synthesis and Antiviral Activity Evaluation of Some Aminoadamantane Derivatives. J. Med. Chem..

[cit218] Kolocouris A. , Synthesis of Heterocyclic Aminoadamantanes of Pharmacological Interest, Ph.D. Dissertation, National and Kapodistrian University of Athens, Athens, Greece, 1995

[cit219] Todd M., Hrdina R. (2023). Synthesis of 1,2-Disubstituted Adamantane Derivatives by Construction of the Adamantane Framework. Molecules.

[cit220] Pardali V., Giannakopoulou E., Konstantinidi A., Kolocouris A., Zoidis G. (2019). 1,2-Annulated Adamantane Heterocyclic Derivatives as Anti-Influenza α Virus Agents. Croat. Chem. Acta.

[cit221] Tzitzoglaki C., Wright A., Freudenberger K., Hoffmann A., Tietjen I., Stylianakis I., Kolarov F., Fedida D., Schmidtke M., Gauglitz G., Cross T. A., Kolocouris A. (2017). Binding and Proton Blockage by Amantadine Variants of the Influenza M2 _WT_ and M2 _S31N_ Explained. J. Med. Chem..

[cit222] Li F., Ma C., Degrado W. F., Wang J. (2016). Discovery of Highly Potent Inhibitors Targeting the Predominant Drug-Resistant S31N Mutant of the Influenza A Virus M2 Proton Channel. J. Med. Chem..

[cit223] Li F., Ma C., Hu Y., Wang Y., Wang J. (2016). Discovery of Potent Antivirals against Amantadine-Resistant Influenza A Viruses by Targeting the M2-S31N Proton Channel. ACS Infect. Dis..

[cit224] Wang J. J., Wu Y., Ma C., Fiorin G., Pinto L. H., Lamb R. A., Klein M. L., Degrado W. F. (2013). Structure and Inhibition of the Drug-Resistant S31N Mutant of the M2 Ion Channel of Influenza A Virus. Proc. Natl. Acad. Sci. U. S. A..

[cit225] Tzitzoglaki C., Hoffmann A., Turcu A. L., Schmerer P., Ma C., Laros G., Liolios C., José B., Wang J., Vázquez S., Schmidtke M., Kolocouris A. (2022). Amantadine Variant – Aryl Conjugates That Inhibit Multiple M2 Mutant – Amantadine Resistant Influenza a Viruses. Eur. J. Med. Chem. Rep..

[cit226] Wu Y., Canturk B., Jo H., Ma C., Gianti E., Klein M. L., Pinto L. H., Lamb R. A., Fiorin G., Wang J., Degrado W. F. (2014). Flipping in the Pore: Discovery of Dual Inhibitors That Bind in Different Orientations to the Wild-Type versus the Amantadine-Resistant S31n Mutant of the Influenza a Virus M2 Proton Channel. J. Am. Chem. Soc..

[cit227] Vauquelin G. (2016). Effects of Target Binding Kinetics on in Vivo Drug Efficacy: Koff, Kon and Rebinding. Br. J. Pharmacol..

[cit228] Zhao X., Li C., Zeng S., Hu W. (2011). Discovery of Highly Potent Agents against Influenza A Virus. Eur. J. Med. Chem..

[cit229] Li F., Hu Y., Wang Y., Ma C., Wang J. (2017). Expeditious Lead Optimization of Isoxazole-Containing Influenza A Virus M2-S31N Inhibitors Using the Suzuki − Miyaura Cross-Coupling Reaction. J. Med. Chem..

[cit230] Tzitzoglaki C., Hoffmann A., Turcu A. L., Schmerer P., Ma C., Laros G., Liolios C., José B., Wang J., Vázquez S., Schmidtke M., Kolocouris A. (2022). Amantadine Variant – Aryl Conjugates That Inhibit Multiple M2 Mutant – Amantadine Resistant Influenza a Viruses. Eur. J. Med. Chem. Rep..

[cit231] Musharrafieh R., Ma C., Wang J. (2020). Discovery of M2 Channel Blockers Targeting the Drug-Resistant Double Mutants M2-S31N/L26I and M2-S31N/V27A from the Influenza A Viruses. Eur. J. Pharm. Sci..

[cit232] Musharrafieh R., Lagarias P. I., Ma C., Tan G. S., Kolocouris A., Wang J. (2019). The L46P Mutant Confers a Novel Allosteric Mechanism of Resistance Toward the Influenza A Virus M2 S31N Proton Channel Blockers. Mol. Pharmacol..

[cit233] Li F., Ma C., Degrado W. F., Wang J. (2016). Discovery of Highly Potent Inhibitors Targeting the Predominant Drug-Resistant S31N Mutant of the Influenza A Virus M2 Proton Channel. J. Med. Chem..

[cit234] Li F., Ma C., Hu Y., Wang Y., Wang J. (2016). Discovery of Potent Antivirals against Amantadine-Resistant Influenza A Viruses by Targeting the M2-S31N Proton Channel. ACS Infect. Dis..

[cit235] Li F., Hu Y., Wang Y., Ma C., Wang J. (2017). Expeditious Lead Optimization of Isoxazole-Containing Influenza A Virus M2-S31N Inhibitors Using the Suzuki − Miyaura Cross-Coupling Reaction. J. Med. Chem..

